# Listeners’ perceptions of the certainty and honesty of a speaker are associated with a common prosodic signature

**DOI:** 10.1038/s41467-020-20649-4

**Published:** 2021-02-08

**Authors:** Louise Goupil, Emmanuel Ponsot, Daniel Richardson, Gabriel Reyes, Jean-Julien Aucouturier

**Affiliations:** 1 STMS UMR 9912 (CNRS/IRCAM/SU), Paris, France; 2 University of East London, London, UK; 3 Laboratoire des Systèmes Perceptifs, Département d’Études Cognitives, École Normale Supérieure, PSL University, CNRS, Paris, France; 4 Hearing Technology - WAVES, Department of Information Technology, Ghent University, Ghent, Belgium; 5 University College London, London, UK; 6 Universidad del Desarrollo, Santiago, Chile; 7 FEMTO-ST (FEMTO-ST UMR 6174, CNRS/UBFC/ENSMM/UTBM, Besançon, France

**Keywords:** Cognitive neuroscience, Human behaviour

## Abstract

The success of human cooperation crucially depends on mechanisms enabling individuals to detect unreliability in their conspecifics. Yet, how such epistemic vigilance is achieved from naturalistic sensory inputs remains unclear. Here we show that listeners’ perceptions of the certainty and honesty of other speakers from their speech are based on a common prosodic signature. Using a data-driven method, we separately decode the prosodic features driving listeners’ perceptions of a speaker’s certainty and honesty across pitch, duration and loudness. We find that these two kinds of judgments rely on a common prosodic signature that is perceived independently from individuals’ conceptual knowledge and native language. Finally, we show that listeners extract this prosodic signature automatically, and that this impacts the way they memorize spoken words. These findings shed light on a unique auditory adaptation that enables human listeners to quickly detect and react to unreliability during linguistic interactions.

## Abstract (web summary)

It remains unclear whether and how listeners can infer speakers’ reliability from the sound of their voice. Here, the authors show that listeners from several languages rely on a common prosodic signature to infer whether speakers are lying or doubting.

## Introduction

Peers are not always reliable, either because they possess erroneous beliefs, are not willing to share their knowledge, or even intentionally try to deceive others^1–3^. When making collective decisions, exchanging information, or learning from others, it is therefore crucial to evaluate both the certainty that social partners have in the information they are providing (i.e., how much they believe the information they possess to be true) and how honest they are (i.e., whether they are actually communicating the information they believe to be true)^1,4,5^. It has been argued that humans possess dedicated mechanisms of epistemic vigilance, allowing them to detect when a person should not be trusted^1^. Such mechanisms would enable cumulative culture to materialize and persist in humans, because they ensure that unreliable information does not spread easily^1,6^, and enable groups to cooperate efficiently by weighting individuals’ contributions depending on their degree of certainty^4^. Yet, there is still much to explore about the perceptual, cognitive, and metacognitive mechanisms that support the detection of certainty and honesty in social partners.

Natural languages typically possess specific markers allowing speakers to explicitly and deliberately communicate their level of certainty^7–9^. These linguistic markers may consist in dedicated expressions such as “I don’t know”^8^ or rely on more indirect systems of evidentials whereby speakers point towards the source of their knowledge^7^, or on socio-pragmatic means^7,9^. Optimally sharing certainty in such explicit ways is costly however, since partners need to calibrate the way they communicate their confidence to one another^10^, converge on shared linguistic expressions through effortful processes involving conversational alignment and cultural learning^8,11^, and rely on analytical (or system 2) thinking^1,6,12^. Yet, the highly adaptive function of epistemic vigilance^1,6^ and the fact that even young children filter information from unreliable informants^5^ suggest that simpler, low-level mechanisms may have evolved to enable the fast and automatic detection of unreliability in social partners, across languages and cultures.

Consequently, other streams of research have focused on paralinguistic markers and provided some evidence that listeners can infer speakers’ levels of certainty from the insertion of pauses (i.e., hesitations) or fillers (e.g., “huum”), dedicated gestures (i.e., flipping palms or shrugging), and specific prosodic signatures^9,13–15^. Research in this field typically involves elicitation procedures comprising two phases^9,13,14,16^. First, encoders (trained actors^14,17^ or speakers in a semi-naturalistic setting^13,16^) are recorded while expressing utterances with various levels of certainty. In a second phase, acoustic analysis of these recordings is performed, and listeners are asked to recover the degree of certainty expressed by the speakers. Acoustic analyses of these recordings typically reveal that speakers’ uncertainty is associated with decreased volume and rising intonation^9,13,14,18^, and to a lesser extent higher^14^ (yet also sometimes lower^16^) mean pitch as well as slower^13,14,16,19^ (yet also sometimes faster^18^) speech rate.

While these studies show that listeners are able to infer speakers’ uncertainty from the sound of their voice^13,14,16,19,20^, the precise perceptual representations used by listeners to perform these judgments remain unclear. First, because these prosodic signatures are typically examined in procedures where speakers deliberately produce them, it is unknown whether, at a fundamental level, they are inherently communicative (i.e., natural or conventional signals)^21,22^ as opposed to constituting natural signs^22^, e.g., of cognitive effort^18,19,23^ (throughout the paper, we use “natural” by opposition with “conventional” to refer to meaning that relies on intrinsic and recurrent associations, rather than arbitrary, culturally learned conventions^24,25^). Second, because they critically depend on how speakers encode the target attitude in the first place, these studies offer no guarantee that what is encoded by the speaker actually corresponds to genuine prosodic signatures of certainty: speakers asked to display certainty may also convey social traits such as dominance (associated with lower pitch) or trustworthiness (associated with higher pitch)^26,27^; these social traits may mediate subsequent ratings of certainty, but the corresponding vocal signatures (e.g., mean pitch) cannot be presumed to be inherently related to certainty. Investigating separately the perception and production of these prosodic signatures is crucial in this regard: although of course they are intimately linked, they do not always rely on the same underlying mechanisms. For instance, mean pitch is not strictly tied to body size in speech production (formant dispersion is), still, listeners use this information to perceive body size and related social traits such as dominance because of a general perceptual bias linking low pitch with largeness^27^. Third, there are important differences between portrayed and spontaneous prosodic displays^28^: actors’ productions may reflect stereotypical rather than veridical expressions, and in addition, they may not be aware of all the prosodic signatures that are naturally produced and used by listeners to perceive honesty and certainty. Finally, because acoustic features typically co-vary in speech production^29^, such paradigms do not allow examining how speech rate, pitch, and loudness statically and dynamically impact listeners’ perception independently from one other. In short, because these procedures are correlational in nature, they do not inform us about the underlying perceptual, cognitive, and metacognitive mechanisms that drive these judgments.

Interestingly, the markers of uncertainty identified in these studies closely resemble the acoustic signatures that have been associated with mental and articulatory effort: the tension and frequency of vocal fold vibrations—and thus pitch and pitch variability—increase with cognitive load^30^ and psychological stress^31,32^. Higher effort is also associated with slower and more variable articulation rate^30,33^, and with a disruption of the “default” pattern associating higher pitch and volume to the beginning of an utterance, and lower pitch and volume to the end of the utterance, a natural consequence of the decrease in subglottal air pressure during the exhalation phase of breathing^23,34^. Relatedly, prosodies intended to be neutral can actually be judged to reflect certainty^14^. Given the link between uncertainty and cognitive (dis)fluency^35,36^, it is probable that the prosodic signatures typically associated with states of uncertainty essentially constitute natural signs of cognitive effort, stemming from physiological constrains on speech production^23^. If such was the case, we might expect that the same core prosodic signature may be used for other social evaluations related to speaker reliability that are also thought to involve increased cognitive effort, such as lying^2,37^.

Genuine communication of certainty occurs when senders are actually willing to share their true commitment to the proposition they express^38^. Yet, it would also be adaptive to have means to detect speakers’ commitment to a proposition when they are not willing to share this information (e.g., when they are trying to deceive). Even though lying speakers—by definition—do not deliberately signal their unreliability, dishonesty may also be detected from prosody if speakers involuntarily manifest signs of cognitive disfluency. Yet, while lying is thought to be associated with increased cognitive effort^2,37^, research examining whether this has stable behavioral consequences has produced mixed results. Thus, whether humans are actually able to exploit behavioral cues to spot liars remains mysterious despite intense scrutiny^2,3^. If anything, speech prosody is thought to carry more reliable markers of deception than other behaviors, such as gaze aversion^2,39–41^. Similarly to doubt, pitch tends to increase^39,41^ during lies, and speech rate to decrease, although this latter relationship is less reliable^2,42,43^.

Taken together, these two separate strands of literature suggest that a common, core prosodic signature of cognitive effort may in fact support both social perceptions of certainty and honesty^3,23^. Here we directly test this hypothesis in 4 studies involving 115 listeners. In a first experiment, we use psychophysical reverse correlation to identify the perceptual representations used by listeners to infer the honesty and certainty of a speaker in a decontextualized, forced-choice task, and find that both types or judgments are supported by strikingly similar perceptual representations across three acoustic features: intonation, loudness, and speech rate. In three additional experiments, we then acoustically manipulate speech stimuli to display this common prosodic signature. This allows us to: (1) provide mechanistic evidence that this prosodic signature is indeed implicated in both types of judgments in a contextualized situation (Study 2A), and to show that (2) it is processed independently from participants’ concepts about epistemic prosody (Study 2B), (3) it is perceived cross-linguistically (Study 3), and (4) it automatically impacts verbal working memory (Study 4), as would be expected of a core prosodic signature originating from physiological reactions associated with cognitive effort (i.e., of a natural sign) as opposed to a culturally learned convention.

Finally, if social perceptions of a speaker’s certainty and honesty rely on similar perceptual inputs, they may differ at higher levels of processing to allow listeners to differentially interpret these signatures for one or the other judgment. For social perceptions of certainty, speakers’ intentions should make little difference in interpreting the prosodic displays, since, whether they are deliberately produced^14,16^ or automatically shown^18,19^, similar displays are observed. Crucially however, there is an important asymmetry in the case of dishonesty: on the one hand, signatures of cognitive effort may involuntarily be disclosed by a deceitful speaker, but on the other hand, a display suggestive of little cognitive effort may be deliberately shown to simulate certainty^2,16^. Thus judgments about dishonesty can hardly reduce to perceptual decisions and would necessarily engage additional inferences (e.g., is the situation cooperative or competitive? what is the social cost of signaling dishonesty? etc.) in order to infer speakers’ true intentions and interpret the display^2,3,6^. Given this, we hypothesized that, while judgments about honesty and certainty may rely on similar perceptual inputs, the type of inferences made upon these inputs would differ and lead to potentially different outcomes for contextualized judgments. By comparing judgments about certainty and dishonesty made in different contexts, but on the basis of the same stimuli (Study 2A), we show that, indeed, providing listeners with additional information regarding speakers’ incentives (e.g., that they are potentially trying to deceive) has no impact on participants’ interpretation of the prosodic signature for judgments of certainty, but introduces important inter-individual variability for judgments of dishonesty.

Taken together, these results provide a comprehensive account of the perceptive, cognitive, and metacognitive mechanisms that allow human listeners to quickly detect and react to unreliability during linguistic interactions.

## Results

In the first study, instead of relying on actors to produce stereotypical expressions of certainty and honesty^16,17^, we took inspiration from a recent series of data-driven studies in visual cognition, in which facial prototypes of social traits were derived from human judgments of thousands of computer-generated visual stimuli^44,45^. Using a similar psychophysical technique—reverse correlation^22^—we manipulated the pitch, duration, and loudness of spoken pseudo-words with acoustic signal-processing algorithms^21^ in order to create random prosodies, thereby sampling a large space corresponding to the range of naturally produced speech (see “Methods”). We asked 20 (11 females) native speakers of French to evaluate the certainty and honesty of a speaker in 2 distinct testing sessions separated by 1 week. Each participant heard 880 pairs of these randomly manipulated stimuli, each pair being matched in terms of pseudo-word and speaker identity (thus canceling out their contribution). Participants were asked to indicate which of the two exemplars sounded more dishonest (in one session) or certain (in the other session, the order of the sessions was counterbalanced across participants), before stating how confident they were in their judgment. For each participant and each task (certainty/honesty), we computed perceptual representations in the form of normalized temporal kernels. We subtracted the pitch, loudness, and duration of the voices classified as reliable (i.e., honest or certain) from the pitch, loudness, and duration of the voices classified as unreliable (i.e., dishonest or doubtful). This was done for 12 temporal points in a word for pitch and loudness and 5 for duration (see “Methods” for details).

Thus, instead of acoustically analyzing naturalistic speech, here we use listeners’ classifications of randomly manipulated pseudo-words to reconstruct the perceptual representations that underlie their judgments in an agnostic, data-driven manner. This procedure has three main advantages over the elicitation paradigm. First, it allows decorrelating perception from production by sampling agnostically from a large feature space rather than focusing on a smaller space sampled by experimenters^46^ or actors^14^ and thus constrained by their own perception. Second, it allows an unconstrained and unbiased test of the hypothesis that listeners’ perception of the certainty and honesty of a speaker rely on a common prosodic signature at the perceptual level, by probing listeners’ representations for the two attitudes separately before comparing them within the same frame of reference. Third, rather than correlating acoustical features with judgments, this reverse correlation procedure amounts to building a computational model in which observers make perceptual decisions by comparing exemplars to a fixed internal template (or, in the language of Volterra/Wiener analysis, a kernel), which the above procedure learns from decision data^47^. This model can then be tested in a causal manner, by acoustically manipulating novel speech stimuli to match participants’ kernels, and test their consequence on judgments made by other participants, as we do in Study 2, 3, and 4. Depending on the strength and precision of listeners’ internal representations, classification judgments would be more or less precise, and the kernels recovered through this procedure would deviate from baseline accordingly (e.g., a listener who does not have any internal representation concerning honest prosodies would show a flat kernel that would not deviate from chance level).

A last important aspect of the first study is that we relied on a two-alternative forced-choice procedure, which not only allows bypassing individual decisional biases (e.g., a truth-bias where observers tend to assume that speakers are honest^3,40^, or conversely, a lie-bias^2^) but also assessing the specific contribution of prosody to social perceptions (the effects of word and speaker identity being discarded because stimuli of a given pair were similar for these aspects). This allows us to specifically uncover perceptual representations of certainty/honesty in a context-free and unbiased manner, contrary to absolute, continuous judgments (e.g., on a Likert scale) typically used in past studies^14,46^, that reflected a mixture of perceptual and decisional processes.

### A common prosodic signature supports listeners’ perceptions of honesty and certainty (Study 1)

**Fig. 1:**
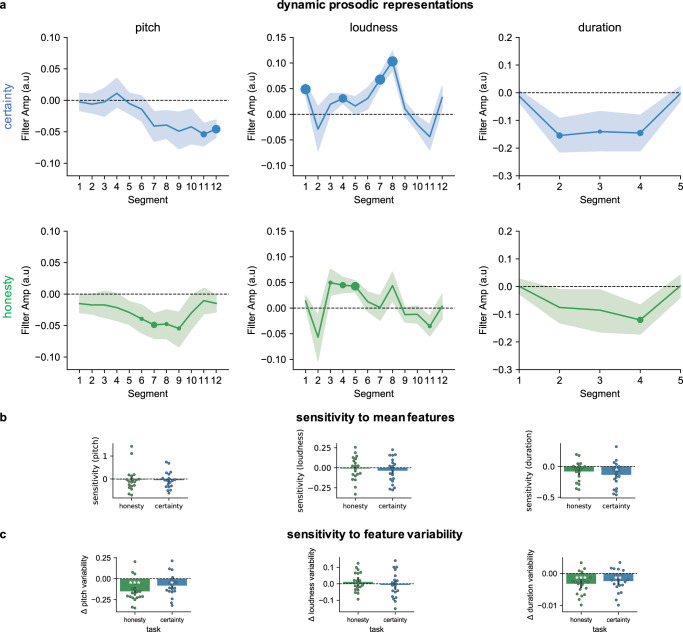
Reverse correlation results (study 1). **a** Dynamic prosodic representations. Normalized kernels derived from the reverse correlation analyses in both tasks (top: certainty, blue; bottom: honesty, green) across the three acoustical dimensions (pitch, loudness, and duration). Filter amplitudes (a.u., arbitrary units) correspond to the values obtained for each participant, task, acoustic dimension, and segment by subtracting the average (pitch, loudness, and duration) values obtained for stimuli judged as certain/honest from the values averaged for the unchosen stimuli and normalizing these values for each participant by dividing them by the sum of their absolute values. Data show group averages, with shaded areas showing the SEMs. Significant deviations from zero (one-sample two-sided *t* tests) are indicated at the corresponding segment positions by circles, with increasing sizes corresponding to *p* < 0.1; *p* < 0.05; *p* < 0.01, and *p* < 0.001; certainty task (*p* values per segment for pitch: 0.86, 0.69, 0.91, 0.64, 0.77, 0.49, 0.11, 0.11, 0.13, 0.14, 0.01, 0.004; loudness: 0.0005, 0.51, 0.37, 0.007, 0.38, 0.18, 0.0001, 0.0001, 0.44, 0.22, 0.12, 0.16; duration: 0.6, 0.03, 0.07, 0.04, 0.94); honesty task (pitch: 0.33, 0.29, 0.44, 0.34, 0.14, 0.09, 0.03, 0.06, 0.08, 0.30, 0.62, 0.30; loudness: 0.29, 0.24, 0.07, 0.01, 0.002, 0.53, 0.96, 0.17, 0.42, 0.5, 0.098, 0.88; duration: 0.98, 0.24, 0.30, 0.048, 0.94). Kernels were computed for 5 time points for duration (corresponding to the initial values of the audio transformations) and in 12 time points for pitch and duration (corresponding to post-transformation acoustic analysis of the stimuli, see “Methods”). Individual raw (i.e., non-normalized) kernels are shown in Fig. SII.a.**b** Sensitivity to mean features. To assess the extent to which mean pitch, loudness, and duration affected participants’ judgments at a static level, we constructed for each participant and task psychometric functions relating sensory evidence (computed for each trial as the area under the curve corresponding to the difference between the dynamic profiles of the first minus second stimuli) to participant’s choices (i.e., the probability to choose the first stimulus). Bar plots show the slopes averaged over the group separately in each task, with error bars showing the SEM. Dots show individual data. The white asterisk shows the result of one-sample Wilcoxon signed-rank test with *p* < 0.05; pitch (0.33/0.19), loudness (0.4/0.84), duration (0.012/0.053). **c** Sensitivity to feature variability. For each trial, the standard deviation of the pitch, loudness, and duration for the stimuli judged as more reliable (honest, certain) were subtracted from the stimuli judged as less reliable (lying, doubtful; Δ: difference). Bar plots show the slopes averaged over the group separately in each task, with error bars showing the SEM. Dots show individual data. White asterisks show the result of one-sample *t* test against chance with *p* < 0.05; **p < 0.01; ***p < 0.001; pitch (certainty *p* = 0.017/honesty *p* = 0.0002); loudness variability (0.7/0.4); duration variability (0.009/0.0007). Source data are provided as a Source data file.

Despite the fact that both tasks (certainty/honesty) were separated by an interval of 1 week, and the large variety of random tokens presented to the listeners, we found that the perceptual representations obtained for honesty and certainty were strikingly similar for all three acoustic dimensions (Fig. 1a). Below, we detail the results for each acoustic dimension, first reporting how sensory evidence dynamically impacted participants’ judgments (Fig. 1a), before describing global effects, assessing how mean pitch, loudness, and duration (Fig. 1b), and the variability of each acoustic feature (Fig. 1c), impacted participants’ judgments.

Regarding pitch, a linear mixed regression, including participant as a random factor and segment, task (certainty/honesty), and their interaction as fixed factors, revealed a significant linear effect of segment (X2 = 22.76, *p* = 0.02, *t* = −3.65, beta = −0.04 +/− 0.01 sem), no main effect of task (X2 = 0.25, *p* = 0.61, *t* = 0.5, beta = 0.003 +/− 0.004 sem), and no interaction between segment and task (X2 = 12.24, *p* = 0.35, *t* = −1.95, beta = −0.02 +/− 0.01 sem). The impact of segment reflected the fact that, as can be seen on Fig. 1a, falling intonations were perceived as more certain/honest. This is consistent with the fact that, in production, speakers’ certainty is specifically related to lower pitch toward the end of the word^14^, over and beyond other aspects such as sensory evidence or accuracy^18^. Importantly, there was no significant effect of task, and no interaction between segment and task, suggesting that the shape of the kernels was equivalent for both certainty and honesty.

**Fig. 2:**
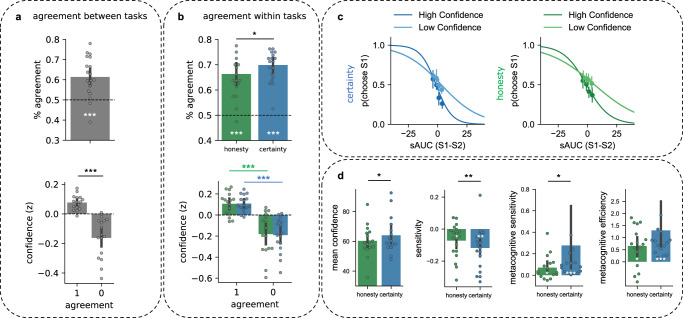
Stability and precision of the perceptual decisions made in the two tasks. **a** Top: percentage of agreement across the two tasks (computed as the percentage of trials in which stimuli were classified similarly: voices classified as certain and honest versus doubting and lying correspond to an agreement). White asterisks show the significance of the result of the two-sided *t* test comparing the percentage of agreement between tasks with chance level (50%) and reported in the main text, with *** corresponding to *p* < 0.001. Bottom: normalized (*z*-scored) confidence ratings averaged separately for agreements and disagreements. Black asterisk shows the result of the two-sided *t* test comparing confidence for agreements versus disagreements reported in the main text, with *** corresponding to *p* < 0.001. Data are presented as mean values with error bars showing the 95% confidence interval. Dots show individual data. **b** Top: percentage of agreement within each task, computed as the percentage of double-pass trials in which stimuli were classified similarly. White asterisks show the significance of the result of the two-sided *t* test comparing the percentage of agreement within each task with chance level (50%) reported in the main text, with *** corresponding to *p* < 0.001. The black asterisk shows the results of the two-sided *t* test comparing the two tasks reported in the main text; *p = 0.02. Bottom: confidence ratings depending on agreement in the honesty (green) and certainty (blue) tasks. Green (honesty task) and blue (certainty task) asterisks show the result of the two-sided *t* test comparing confidence for agreements versus disagreements within each task, with *** corresponding to *p* < 0.001. Data are presented as mean values with error bars showing the 95% confidence interval. Dots show individual data. **c** Probability of responding that the first voice (*p*(choose S1)) sounds more certain (left, blue) or honest (right, green) as a function of the area under the curve computed by subtracting sensory evidence for the first minus the second stimuli, summed for the three acoustic dimensions. Darker lines correspond to high confidence trials (above the median) and lighter lines to low confidence trials (below the median). Circles show mean values and error bars the 95% confidence interval. **d** Average confidence, sensitivity, metacognitive sensitivity, and efficiency in the honesty and certainty tasks. Data represent mean values with error bars showing the 95% confidence interval, and dots show the individual data; black asterisks show the result of the two-sided tests comparing the two tasks, and white asterisks show the results of two-sided tests against chance level; *t* tests were used for confidence (normally distributed data), and Wilcoxon signed-rank tests for sensitivity, metacognitive sensitivity, and efficiency (non-normal data); *p < 0.05; **p < 0.01; ***p < 0.001; confidence: *p* values for the comparison between tasks *p* = 0.037; sensitivity: *p* values testing the difference with chance level, for certainty *p* = 0.0011/honesty, *p* = 0.012; comparison between tasks, *p* = 0.01; metacognitive sensitivity (0.0004/0.034/0.026); metacognitive efficiency (0.0004/0.01/0.72). Source data are provided as a Source data file.

To analyze whether mean pitch also impacted participants’ decisions, we constructed for each participant and each task the psychometric curves relating the difference in pitch between the two stimuli (approximated via the area in between the two dynamic profiles, see “Methods”) to choice probability (see Fig. 1b, and Fig. 2c for slopes computed over the group and all acoustic dimensions). This analysis revealed that mean pitch was not a good predictor of participants’ judgments: slopes did not significantly differ from chance level in the two tasks (certainty: *M* = −0.05 +/− 0.35 SD, *Z*(18) = 71, *p* = 0.33, *d* = 0.14; honesty: *M* = −0.02 +/− 0.5 SD, *Z*(18) = 63, *p* > 0.19, *d* = 0.04; Wilcoxon signed-rank tests were used because slopes were not normally distributed). Thus mean pitch is not a stable feature used by listeners to perceive certainty/honesty, contrary to intonation. This contrasts with previous findings examining prosodic signatures of certainty using elicitation procedures^14,16^, and with a previous study using a similar methodology and sample size, where lower pitch was found to strongly impact judgments about social dominance^26^. By contrast, this is consistent with recent evidence suggesting that speakers’ mean pitch is not necessarily impacted by their certainty in the absence of an audience, while intonation is^18^. Taken together, these findings suggest that mean pitch (i.e., a frequency code) is predominantly relevant for judgments concerned with the personal level (e.g., social traits of dominance), while dynamic pitch variations (i.e., intonation) are more relevant at the attitudinal level (e.g., of certainty, relating to effort or production codes)^23,26^. By contrast, and consistently with the hypothesis that these perceptual representations are related to cognitive effort, pitch variability was a good predictor of participants’ judgments (Fig. 1c): stimuli that were judged to be reliable had less variable pitch (mean standard deviation difference in the honesty task: −0.15 +/− 0.13 SD, one-sample *t* test against zero: *t*(18) = −4.74, *p* < 0.001, *d* = 1.11; certainty task: −0.08 +/− 0.13 SD, *t*(18) = −2.64, *p* = 0.017, *d* = 0.62; the difference between the two tasks was marginal: *t*(18) = 2, *p* = 0.058, *d* = 0.53).

Regarding loudness, there was a significant quadratic effect of segment (X2 = 60.18, *p* < 0.001, *t* = 3.24, beta = 0.05 +/− 0.014 sem), no main effect of task (X2 = 1.68, *p* > 0.19, *t* = 1.3, beta = 0.007 +/− 0.005 sem), and no interaction between segment and task (X2 = 14.34, *p* = 0.21, *t* = −0.37, beta = −0.0005 +/− 0.014 sem). Voices perceived as certain/honest were louder, especially at the beginning of the word. Again, task did not significantly impact kernels, or interact with segment, suggesting relatively preserved shapes across the two types of judgments. At the static level, the slopes of the psychometric functions did not significantly differ from chance level in the two tasks (certainty: *M* = −0.04 +/− 0.15 SD, *Z*(18) = 74, *p* > 0.3, *d* = 0.23; honesty: *M* = −0.01 +/− 0.15 SD, *Z*(18) = 90, *p* > 0.8, *d* = 0.07 (Fig. 1b). Thus, as was the case for pitch, our results highlight the importance of examining dynamic profiles rather than average features: precise patterns of accentuation allow listeners to discriminate certainty and honesty, rather than global increases in volume. Contrary to pitch, variability in loudness did not impact judgments (mean standard deviation difference in the honesty task: −0.012 +/− 0.06 SD, *t*(18) = 0.9, *p* > 0.3, *d* = 0.2; certainty task: −0.007 +/− 0.08 SD, *t*(18) = −0.38, *p* = 0.7, *d* = 0.09; no difference between tasks: *t*(18) = −1.25, *p* > 0.2, *d* = 0.28).

Finally, a similar pattern of results was found for the dynamic analysis of duration: there was a significant quadratic effect of segment (X2 = 14.55, *p* = 0.006, *t* = −3.73, beta = −0.12 +/− 0.03 sem), no main effect of task (X2 = 1.37, *p* > 0.24, *t* = −1.19, beta = −0.02 +/− 0.015 sem), and no interaction between segment and task (X2 = 0.87, *p* > 0.9, *t* = 0.72, beta = 0.02 +/− 0.03 sem). Regardless of the segment, there was also a global effect such that faster voices were more likely to be perceived as certain (mean slope = −0.14 +/− 0.2 SD, *Z*(18) = 33, *p* = 0.012, *d* = 0.67), and marginally so in the case of honesty (mean slope = −0.08 +/− 0.16 SD, *Z*(18) = 47, *p* = 0.053, *d* = 0.5), with a marginal difference in sensitivity between the two tasks (*Z*(18) = 49, *p* = 0.064, *d* = 0.3). Thus, unlike pitch and loudness, duration impacted judgments in a more global fashion. Similar to pitch, variability in speech rate impacted judgments, with more variability associated with less reliability (mean standard deviation difference in the honesty task: −0.003 +/− 0.003 SD, *t*(18) = −4.06, *p* < 0.001, *d* = 0.96; certainty task: −0.002 +/− 0.003 SD, *t*(18) = −2.94, *p* = 0.008, *d* = 0.69; no difference between tasks: *t*(18) = 1.3, *p* > 0.2, *d* = 0.25).

Overall, there was very little difference between the two tasks, which suggests that a common prosodic signature subtends both types of judgments (also see Fig. SI for the global kernels collapsed across the two tasks). Importantly, there was no impact of task order on the kernels (i.e., whether the certainty or the honesty task was performed first; all *p* values >0.4), no interaction between task order and segment (all *p* values >0.5), nor task order, segment, and task (all *p* values >0.4) for any of the three acoustic dimensions. Thus the kernels were not affected by whether participants performed one or the other task before, which rules out an interpretation in terms of carry-over effects (i.e., participants keeping a strategy developed during the first task to perform the second task).

Because we observed inter-individual differences between these kernels among the different listeners tested (see Fig. SIIA), we conducted further analyses to confirm the similarity of the kernels between the two tasks at an individual level, complementing our above group-level conclusions. We found that the correlations between the kernels of the same individuals across the two tasks were significantly higher than the correlations between the kernels of different individuals within the same task or across the two tasks (see Fig. SIIB). Additional analyses also revealed that there were differences in how female and male listeners used specific prosodic dimensions to categorize stimuli, with male participants being more sensitive to loudness and duration than females, but strikingly, these gender differences were reflected similarly in both tasks (see Fig. SIII). Thus, despite some idiosyncrasies regarding how acoustic dimensions are weighted against one another, and substantial inter-individual differences regarding the exact shape of the kernels, each participant represented certain and honest prosodies similarly at the perceptual level.

In summary, voices were perceived to be unreliable (i.e., doubtful or lying) if they had rising intonation, less intensity at the beginning of each syllable, and slower speech rate. These results—obtained through a data-driven method, and thus not subject to biases stemming from experimenters’ expectations and perception of the stimuli—are in line with previous observations examining honest and certain prosodies separately with actor-produced expressions concerning intonation^14,39,42^. By contrast, they suggest that other aspects, in particular mean pitch, are not specifically discriminative when prosodic dimensions are also dynamically manipulated, which might explain previous discrepancies in the literature^14,16^.

### Overlap between the two types of judgments evidenced through choice consistency across the two tasks (Study 1)

To further examine the proximity of the judgments given for the two social attitudes, we examined the percentage of agreement between responses given across the two tasks (by analogy with the double-pass consistency technique, see below), since they were based on the exact same pairs of stimuli (for each pair, agreement = 1 if the same stimulus was classified both as certain and honest, agreement = 0 otherwise). Agreement (*M* = 61.4% +/− 9 SD) was highly significantly above chance (*t*(18) = 5, *p* < 0.001, *d* = 1.18) and remarkably high considering the large number of exemplars heard by the participants, and the fact that they performed the two tasks with 1 week apart (see Fig. 2a). In addition, participants were more confident in their choices when they provided converging (0.08 +/− 0.05) as compared to diverging judgments (−0.16 +/− 0.13, *t*(15) = 5.2, *p* < 0.001, *d* = 2.48; 3 participants were excluded from analysis regarding confidence because they did not use the scale appropriately, see “Methods”). This indicates that the exemplars that were judged consistently in the two tasks were easier to classify, probably because they better matched mental representations of unreliable or reliable prosodies.

### Judgments about honesty are less stable and less precise that judgments about certainty (Study 1)

To evaluate the stability of listeners’ judgments within each task, we employed a double-pass consistency technique^48,49^: without the participants’ knowing, 10% of the trials were presented twice in each task. This allowed us to compute the percentage of times that participants provided the same judgments for the same pair of stimuli within each task. As can be seen in Fig. 2b, the percentage of agreement was high within the same task. Participants provided the same judgments 69.9% +/− 6.4 SD of the time in the certainty task and 66.3% +/− 9.3 SD of the time in the honesty task, which was highly significantly above chance (certainty: *t*(18) = 13, *p* < 0.001, *d* = 3.1; honesty: *t*(18) = 7.4, *p* < 0.001, *d* = 1.75). Within task, consistency was significantly lower when judging honesty than when judging certainty (*t*(18) = 2.5, *p* = 0.02, *d* = 0.45). In addition, the percentage of agreement was correlated across tasks: participants who were the most consistent in the certainty task were also the most consistent in the honesty task (Spearman’s rho = 0.69, *p* = 0.001, see Fig. SIV.A). Finally, participants were more confident in their choices when they provided converging (certainty: 0.12 +/− 0.1 SD; honesty: 0.11 +/− 0.1 SD) as compared to diverging judgments (certainty: −0.17 +/− 0.16 SD; *t*(18) = 6.44, *p* < 0.001, *d* = 2.3; honesty: −0.17 +/− 0.19 SD; *t*(18) = 4.6, *p* < 0.001, *d* = 1.89, see Fig. 2b).

We further verified the robustness of these findings with respect to the response strategy of each individual by converting the values of percentage of agreement into values of internal noise using a signal-detection theory (SDT) model^48,49^ accounting for response bias across intervals (see “Methods” and Fig. SIV.B). Internal noise values for both tasks (*M* ± SD: honesty: *M* = 1.22 +/− 0.76 SD, certainty: *M* = 1.05 +/− 0.66 SD; expressed in units of external noise standard deviation) were consistent with both low-level psychophysical tasks^50^ and high-level cognitive auditory tasks^51^. We reached similar conclusions as with the percentage of agreement: (i) internal noise was lower for certainty compared to honesty (*t*(17) = 2.23, *p* = 0.04, *d* = 0.14, and (ii) these values were correlated between both tasks (Spearman rho = 0.55, *p* = 0.02, Fig. SIV.C), supporting the view that participants who were the most consistent in the certainty task were also the most consistent in the honesty task. It is unlikely that the observed effects stem from differences in task engagement or instructions between tasks. Rather, the present results point toward differences in terms of the precision of the perceptual representations used by listeners to perform each task, or alternatively, differential consistency in using perceptual representations to make the judgments (see Study 2).

Overall, the findings show that the perceptual representations guiding judgments in the two tasks are stable (e.g., internal noise was equivalent to what can be observed in low-level perceptual tasks) and that they largely overlap for the two tasks. Yet, they also show that judgments about the certainty of a speaker are tied to sensory evidence more than judgments about honesty. This difference was further quantified by computing for each participant and task a psychometric function relating their decisions to the quantity of evidence available in each trial, cumulated over the three acoustic dimensions (see Fig. 2c and “Methods”). Sensitivity, corresponding to the slope of individuals’ psychometric functions, significantly differed from chance level in both tasks with this cumulative measure (certainty: *M* = −0.12 +/− 0.13 SD, *Z*(18) = 14, *p* = 0.0011, *d* = 0.93; honesty: *M* = −0.07 +/− 0.1 SD, *Z*(18) = 33, *p* = 0.012, *d* = 0.7). Congruent with the analyses of choice agreement and internal noise presented above, sensitivity was lower in the honesty as compared to the certainty task (*Z*(18) = 31, *p* = 0.01, *d* = 0.41).

### Confidence in social perceptions of honesty and certainty (Study 1)

Consistent with this decreased precision, participants were also less confident overall in their judgments about honesty (*M* = 64.1 +/− 12) as compared to certainty (*M* = 60.3 +/− 10.3; *t*(15) = 2.29, *p* = 0.037, *d* = 0.35, see Fig. 2d). This would be expected if evaluating certainty can indeed reduce to identifying a display (i.e., making a perceptual decision), while evaluating honesty cannot.

To examine whether this decreased precision impacted metacognitive sensitivity (i.e., the ability of participants to track the reliability of their decisions through confidence judgments), we computed an index of metacognitive sensitivity for each participant in each task, by subtracting the slope of the psychometric functions built for high-confidence judgments from the slope obtained for low-confidence judgments^52^ (see “Methods”). Metacognitive sensitivity was significantly above chance level in both tasks (certainty: *M* = 0.28 +/− 0.7, *Z*(15) = 0, *p* < 0.001, *d* = 0.55; honesty: *M* = 0.07 +/− 0.11, *Z*(15) = 27, *p* = 0.034, *d* = 0.7, see Fig. 2d and Fig. SV.A for details per acoustic dimensions), but it was higher in the certainty as compared to the honesty task (*Z*(15) = 25, *p* = 0.026, *d* = 0.43). As mentioned above, sensitivity was also lower in the honesty task, which could underlie this difference at the metacognitive level. To assess participants’ ability to evaluate their decisions while considering their underlying sensitivity, we thus computed a last index of metacognitive efficiency (see Fig. 2d and “Methods”). Metacognitive efficiency was above chance level in both tasks (certainty: *M* = 1.3 +/− 2.2, *Z*(15) = 0, *p* < 0.001, *d* = 0.58; honesty: *M* = 0.66 +/− 0.8, *Z*(15) = 19, *p* = 0.011, *d* = 0.82), and there was no significant difference in between the two tasks (*Z*(15) = 61, *p* > 0.7, *d* = 0.4). Thus participants were capable of evaluating the reliability of their decisions when judging which of two voices was more certain and when judging which of two voices was more dishonest. Although we observed decreased sensitivity in the honesty task, results at the level of metacognitive efficiency show that listeners could still evaluate the adequacy of their decisions with respect to sensory evidence. In addition, as was the case for the percentage of agreement and internal noise, participants’ metacognitive efficiency was correlated across the two tasks (Spearman’s rho = 0.53, *p* = 0.034, see Fig. SIV.D). Thus participants who showed higher metacognitive efficiency in one of the tasks also tended to show higher metacognitive efficiency in the other task, in line with previous reports showing that individuals’ levels of metacognitive efficiency correlate across different tasks^53^.

In summary, Study 1 reveals that a common perceptual representation supports both social perceptions of certainty and honesty from speech: despite idiosyncratic strategies, there is a close similarity between the reverse correlation kernels obtained along three acoustic dimensions for social perceptions of honesty and certainty (Fig. 1), and a high degree of agreement in between the two tasks (Fig. 2). In addition, our results show that judgments about the certainty of a speaker are more stable and precise than judgments about the honesty of a speaker in a task that forces participants to rely on speech prosody only. This suggests that, while judgments about the certainty of a speaker from speech prosody can essentially reduce to perceptual decisions, judgments about honesty can hardly be reduced to perceptual decisions and, as suggested by research on deception detection^2,3^, may critically depend on additional contextual information.

### Impact of individual decisional biases and situational context on the interpretation of the common prosodic signature (Study 2A)

To examine the hypothesis that judgments about certainty and honesty rely on a common prosodic signature at a perceptual level, but differ in terms of their reliance on additional, contextual information, we ran a second study. Two separate groups of French-speaking participants heard spoken pseudo-words that were acoustically manipulated to reproduce the prosodic signatures discovered in the first study. They had to rate how much they thought that a speaker was lying (*N* = 20, 9 females) or was certain (*N* = 20, 12 females) on a Likert scale ranging from 1 to 7 in two tasks where contextual information allowing to infer the speakers’ incentives were now accessible. Note that—unlike the forced choice procedure used in Study 1—such absolute ratings reflect a mixture of listeners’ perceptual sensitivity (how attuned to sensory evidence their choices are), the contribution of other acoustical variables (e.g., voice timbre) and cognitive inferences based on these cues (e.g., speaker identity or gender), as well as individual decisional biases (listeners general tendency to report that someone is certain or honest). To construct the stimuli, we applied the average three-dimensional (pitch, loudness, duration) prosodic contours of certainty, honesty, doubt, and lie (i.e., ± the dynamic kernels) inferred from Study 1, each with three different strengths (i.e., three different gain values; see “Methods”), to eight pseudo-words pronounced by two different speakers (same original recordings as in Study 1).

Crucially, a context now provided information about speakers’ potential incentives: for certainty, participants were told that the spoken words were responses recorded from other participants who previously performed a task with various levels of difficulty, and that they were to judge whether these participants were confident in their response or not. For the honesty task, they were told that their task was to judge whether previous participants engaging in a deceitful poker game were lying (i.e., bluffing) or not. Thus participants rated the same stimuli in both tasks, but in one task the framing enforced an interpretation in terms of genuine expressions of certainty, while in the other task the framing suggested that the speakers would sometimes be deceitful and sometimes not. This procedure allowed us 1) to examine the impact of individual decisional biases and contextual factors on the interpretation of the prosodic signatures derived through our reverse correlation method in the first study, and 2) to test the hypothesis that providing listeners with a context can sway their interpretations of these displays when the speaker is likely to be deceitful, while it does not in the case of certainty.

A repeated-measures analysis of variance (rmANOVA) including *z*-scored ratings as a dependent variable revealed a main effect of prosody (*F*(3,114) = 12.2, *p* < 0.001, *η*p2 = 0.2) and an interaction between task and prosody (*F*(3,114) = 24.8, *p* < 0.001, *η*p2 = 0.34), showing that introducing different contexts impacted participants interpretation of the displays. There was also a triple interaction between strength, prosody, and task (*F*(3,114) = 18, *p* < 0.001, *η*p2 = 0.07), reflecting the fact that participants’ ratings varied linearly with the strength of the archetype in the certainty task but not in the honesty task (see Fig. SVI.A).

**Fig. 3:**
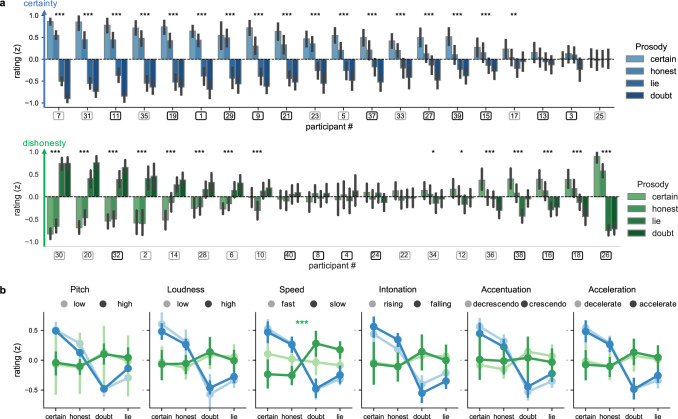
Relationship between perceptual and conceptual knowledge (study 2B). **a** Normalized (*z*-scored) ratings in the certainty (top, blue; *N* = 20) and honesty (bottom, green; *N* = 20) tasks for each participant and prosody type (shown by different hues). Bar plots represent individual participants’ mean normalized ratings for each prosodic archetype, with error bars showing the 95% confidence interval. Data were sorted by effect magnitude. Squared markers below the plot show the listener’s gender (black: female; gray: male). Asterisks show the results of paired two-sided sample *t* tests comparing reliable versus unreliable prosodies for each individual listener, with *p < 0.05; **p < 0.01; ***p < 0.001 (individual *p* values are reported in the Source data file). At the level of the group, in the certainty task, both honest and certain prosodies were judged as more certain than doubtful (honest: *p* < 0.001 Bonferroni corrected post hoc Tukey HSD, *d* = 3.72; certain: *p* < 0.001, *d* = 4.14) and lying (honest: *p* < 0.001, *d* = 3.23; certain: *p* < 0.001, *d* = 3.72) prosodies. In the honesty task, greater inter-individual differences were observed (see detailed report in the main text). **b** Normalized ratings were split depending on participants’ responses at the explicit questions assessing their conceptual knowledge about epistemic prosody, which revealed that the relationship between prosody type and ratings did not vary with participants’ conceptual knowledge about certainty and honesty in general, with the exception of concepts about speed in the honesty task (shown by the green asterisk that represent the significant interaction between concepts about speed and prosody type on ratings of honesty). Data are presented as mean values with error bars showing the 95% confidence interval. Triple asterisks (***) show the significant results of the rmANOVA testing the interaction between concepts about speed and prosody in the honesty task, with normalized ratings as a dependent variable, *p* = 0.0007 (all other interactions were not significant). Source data and exact individual *p* values for **a** are provided as a Source data file.

In the certainty task, where the context enforced a canonical interpretation of the prosodic displays, we confirmed the hypothesis of a common perceptual representation in a distinct sample of participants, in that the archetypes derived from judgments of certainty and judgments of honesty (in the first study) were judged similarly (Figs. 3a and SVIA): certain and honest prosodies were perceived as more certain than lying and doubtful prosodies for every level of strength (all *p* values <0.001, *z*-values >5, post hoc Tukey honestly significant difference (HSD) with Bonferroni correction, *d* ranging from 1.8 for gain = 1, to 2.96 for gain = 3, see Fig. SVIA for the detail for each levels of gain). This is consistent with the hypothesis that listeners interpret this common prosodic signature as a reflection of speaker’s reliability.

The findings also confirmed that judgments about honesty entail more complex inferences and larger inter-individual variability than judgments about certainty. In the honesty task, the magnitude of the effect was much reduced at the group level, and the strength of the transformation had a non-linear effect on judgments. Doubtful prosodies were perceived as more dishonest than honest prosodies for intermediate (*p* < 0.01, *z* > 4, *d* = 0.83) and high levels of strength (*p* < 0.01, *z* > 4, *d* = 0.54), and more dishonest than certain prosodies for intermediate levels of strength (*p* < 0.01, *z* = 4.4, *d* = 0.65). By contrast, judgments were not significantly different between lying prosodies and honest or certain prosodies at the group level (*p* values >0.2, *z*-values <4, all *d* < 0.52, other comparisons were non-significant after Bonferroni correction).

As can be seen in Fig. 3a, this lack of significance at the level of the group was due to increased inter-individual differences in the honesty task. In the certainty task, there was little inter-individual variability: 17 (10 females) out of 20 participants gave significantly higher ratings for honest and certain archetypes as compared to lying and doubtful archetypes (according to paired *t* test on individual data with a significant threshold of *p* = 0.05), no participant provided significantly lower ratings for honest/certain prosodies, and 3 participants did not significantly discriminate between the stimuli. By contrast, 8 (1 female) out of the 20 participants who had to judge whether speakers were lying provided significantly higher ratings for lying/doubtful archetypes than for honest/certain archetypes (i.e., the canonical interpretation), while 7 (4 females) participants presented the opposite pattern, reporting that the speaker was lying upon perceiving canonical displays of reliability (certain/honest prosodies), which suggests that they expected deceitful speakers to fake reliability in this context where bluffing was expected to occur (5 participants did not significantly discriminate between the stimuli, 4 females).

Thus, judgments about certainty were consistently tied to sensory evidence, but judgments about dishonesty were not. Comparing the absolute difference between ratings given for reliable (honest/certain) versus unreliable (lying/doubtful) archetypes between the two tasks revealed no significant difference in the magnitude of the effect however (*t*(38) = 1.7, *p* = 0.1, *d* = 0.57), showing that listeners also largely rely on prosody to make their judgments in the honesty task. Yet, the direction in which individuals interpreted the prosodic signature varied, which reveals that providing listeners with details about speakers’ probable incentives (i.e., to naturally express their confidence or potentially fake it) sways listeners interpretation of the display. Below, we investigate several factors that may explain this large inter-individual variability in the interpretation of the prosodic signature in the honesty task (also see Fig. SVII.A).

### Relationship between perception and conceptual knowledge (Study 2B)

Next, we wanted to determine how listeners’ perception of epistemic prosody relates to their conceptual knowledge. To this end, after they completed the rating task (Study 2A), we asked participants to explicitly report their conceptual knowledge about epistemic prosody, for instance, by stating whether they thought that someone who is certain would speak loudly or quietly. There were six questions in total, targeting the 3 acoustic dimensions of pitch, loudness, and duration, each at a static (e.g., high pitch or low pitch) or dynamic (rising or falling intonation) level.

In sharp contrast with what was observed at the perceptual level, there was no clear consensus among participants at the conceptual level regarding most aspects under study (see Fig. SVI.B). The only aspects for which the distribution of participant’s responses differed significantly from chance associated lower mean pitch to certainty (X2 = 22.5, *p* < 0.001), higher mean pitch to lies (X2 = 19.6, *p* < 0.001), and higher loudness to certainty (X2 = 10, *p* < 0.004; no effect for lies (X2 = 0.9, *p* = 0.44). Thus, although participants rely on canonical prosodic signatures integrating intonation, loudness, and duration to judge whether a voice is lying or doubting, most of the knowledge upon which these judgments rest is not explicitly available to them.

Strikingly, as with the results obtained in the listening tests, there was a strong association between responses given for certainty and honesty at the explicit level (see Fig. SVII.B). Agreement between responses was significantly above chance for five of the six dimensions (mean agreement for loudness: *M* = 72.5% +/− 44, *t*(39) = 3.15, *p* < 0.004, *d* = 0.5; duration: *M* = 75% +/− 43, *t*(39) = 3.6, *p* < 0.001, *d* = 0.57; pitch: *M* = 87.5% +/− 33, *t*(39) = 7, *p* < 0.0001, *d* = 1.13; loudness variations: *M* = 82.5% +/− 38, *t*(39) = 5.34, *p* < 0.0001, *d* = 0.85; duration variations: *M* = 85% +/− 36, *t*(39) = 6.12, *p* < 0.0001, *d* = 0.98; agreement was not significantly different from chance level for intonation: *M* = 62.5% +/− 48, *t*(39) = 1.6, *p* = 0.11, *d* = 0.26). Thus, participants also had common representations regarding lying and doubtful prosodies at the conceptual level, although the content of these representations differed from one individual to the next.

To examine whether this knowledge related to their perception of epistemic prosodies, we split the ratings depending on participants’ responses to the six questions. As can be seen on Fig. 3b, participants’ conceptual knowledge was only weakly associated with their perceptual judgments overall. In the certainty task, none of the aspects assessed at the conceptual level interacted with the effect of prosody type on ratings in an rmANOVA (all *p* values >0.18, *F* < 1.7, *η*p2 < 0.02). In the honesty task, conceptual knowledge about speed interacted with the effect of prosody type on ratings (*F*(3,39) = 7.08, *p* = 0.0007, *η*p2 = 0.29), but none of the other aspects did (all *p* values >0.3, *F* > 1.1; *η*p2 < 0.05). This impact of concepts about speed on ratings also interacted with listeners’ gender as we report in Fig. SVII.A, suggesting two potential mediators of the different interpretations of the display observed in the second study: concepts and identities.

Finally, there were no relationships between participants’ convergence in the two tasks at the conceptual and perceptual levels (see Fig. SVII.C): the degree to which participants’ ratings converged in the two rating tasks did not relate to the degree to which their concepts about the two attitudes converged. Overall, the results show that social perceptions of certainty and honesty based on speech prosody largely rely on procedural rather than declarative knowledge.

### Language specificity (Study 3)

If this prosodic signature of unreliability stems from physiological reactions associated with cognitive effort, rather than from culturally learned conventions, we should expect that it should be perceived cross-linguistically^23,29^. Contrary to cross-cultural perception of vocal emotions, which has received a large amount of attention over the past decades^54^, past studies on prosodic signatures of certainty and honesty examined only one language and attitude at once^13,14,43,46^ or compared the expression or perception of certainty or honesty across languages without actually testing whether these attitudes can be recognized cross-linguistically^43,55^. Only one study indirectly tested whether a composite attitude of doubt/incredulity (coarsely defined as a “feeling of being uncertain or of not believing something”) can be perceived across languages in Japanese, French, and English speakers from speech prosody alone, by relying on an elicitation procedure involving real utterances recorded in the three languages separately by trained speakers^56^. Findings suggested an in-group advantage in recognizing doubt-incredulity, but no acoustic analysis of the stimuli was provided, which prevented the identification of the common prosodic signature underlying these judgments. Moreover, as detailed above, elicitation procedures are not optimal to investigate perception per se, because of a number of confounds that are particularly problematic when investigating cross-cultural issues^57^ (e.g., it is difficult to know what is actually being encoded by the speaker). Thus it remains unknown whether prosodic signatures of reliability can be perceived across languages.

**Fig. 4:**
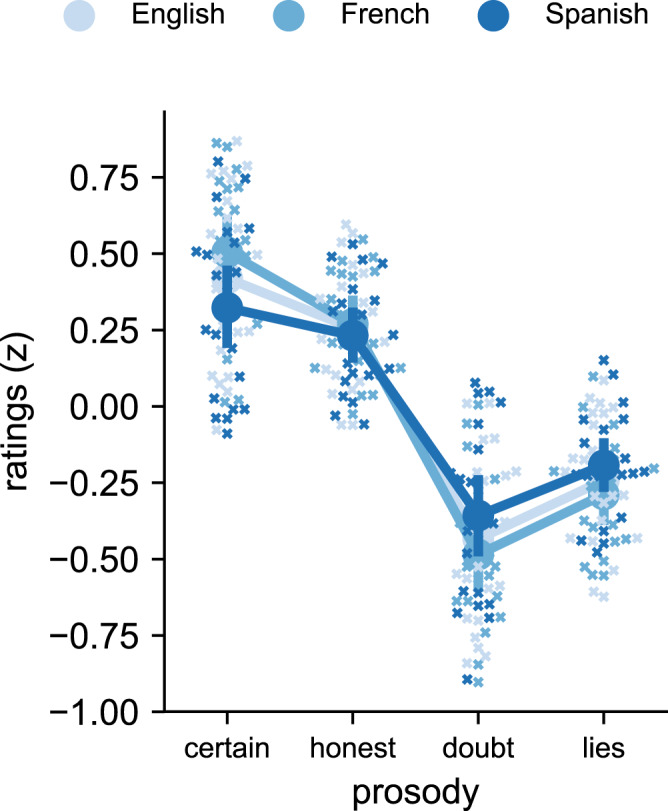
Cross-linguistic validation for the certainty task including the group of French speakers (*N* = 20), a group of native English speakers (*N* = 22), and a group of Spanish speakers (*N* = 21). Normalized ratings (*z*-scored) were averaged separately for each prosodic archetype and language group. Data are presented as mean values with error bars showing the 95% confidence interval. Crosses represent individual data for each prosodic archetype and native language. As was the case in the group of French speakers, Spanish and English speakers perceived certain/honest archetypes to be more certain than doubt/lies archetypes (see main text for details). They also judged certain prosody to be more certain than honest prosodies (*p* < 0.005, *N* = 21 Spanish speakers: *d* = 0.4; *N* = 22 English speakers: *d* = 0.7) and lying prosodies to be more certain than doubtful prosodies (*p* < 0.001, Spanish: *d* = 0.7; English: *d* = 0.8), showing the same sensitivity to small variations in the gain of the archetypes. Source data are provided as a Source data file.

In order to examine this issue, we tested two additional groups of English (*N* = 22) and Spanish (*N* = 21) native speakers who had no exposure to French on the same certainty task as Study 2. As can be seen on Fig. 4, English, Spanish, and French listeners rated the stimuli in the same way. An rmANOVA including the three language groups (French, English, and Spanish) revealed a main effect of prosody on ratings (*F*(3,180) = 135.5, *p* < 0.001, *η*p2 = 0.69), no effect of native language (*F*(2, 60) = 1, *p* > 0.37, *η*p2 < 0.001), and no interaction (*F*(6,180) = 1.4, *p* > 0.2, *η*p2 = 0.045). Post hoc test revealed that participants judged honest and certain prosody to be more certain than lying and doubtful prosodies in every language group (all *p* values <0.001 Bonferroni corrected Tukey HSD and all *d* > 2.2).

In addition, to examine whether language exposure impacts the perception of the prosodic signatures, we tested an additional group of 12 speakers from various languages (German, Dutch, Russian, Marathi, Polish, Japanese, Mandarin Chinese, Swedish) who had various levels of exposure to French. These participants rated the stimuli similarly as the previous groups (see Fig. SVIII.A), and their level of spoken French comprehension was not correlated with their evaluations of the prosodic signatures (Pearson rho = −0.27, *p* > 0.39, see Fig. SVIII.B and “Methods”).

We also reached the same conclusions in the group of English, Spanish, and multi-language speakers regarding the relationship between percepts and concepts (see Fig. SVIII.C): concepts and percepts were largely decoupled, with the only exception that concepts about speed somewhat determined judgments about honesty. As was the case for the group of French participants, there was little consensus at the conceptual level for most aspects: participants did not significantly favor one or the other option for pitch, speed, and intonation (all *p* values > 0.1). The only significant association concerned loudness: participants were more likely to report that a certain voice is louder than a doubtful one.

Overall, these results demonstrate the language independence of a core prosodic signature that underlies both judgments of certainty and honesty. Research has shown that many vocal emotions can be perceived across languages but that there are substantial in-group advantages^54^. Here we find no in-group advantage regarding the perception of reliability in speech, which is consistent with the hypothesis that this prosodic signature reflects natural associations, and is tied to physiological reactions of cognitive effort, rather than culturally learned communicative conventions. Notably, in-group advantages in recognizing vocal emotions increase as cultural and linguistic distance increases^54^, so future research should aim to test remotely related groups rather than Indo-European native speakers. Of particular interest for future research is whether this pattern of results would hold for native speakers of tonal languages^58^ and for native speakers of the few languages that do not conform to the default mode of speech production whereby most utterances present falling intonation and volume (e.g., in languages or dialects where rising intonations are frequently used in statements)^23^. Finally, it may be that in-group advantages are linked to a better familiarity with the utterances carrying the prosodic signatures, as studies finding in-group advantages typically use real words/utterances. An open question is thus whether in-group advantages would emerge in a similar study involving real words/utterances rather than pseudo-words.

### Impact of the common prosodic signature on verbal working memory (Study 3)

**Fig. 5:**
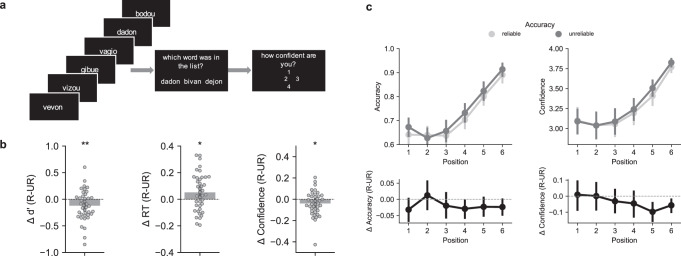
Automatic impact of the common prosodic signature on verbal working memory (study 3). **a** Design of the memorization task. Participants heard six spoken pseudo-words before having to recognize a target pseudo-word presented along with two distractors. Unbeknown to the participant, the spoken targets were pronounced with the archetypes of prosodies derived from Study 1 and were either reliable (certain or honest) or unreliable (lie or doubt), while the prosody of the five spoken distractors was randomly picked from the same pool of stimuli, ensuring equal saliency of the target and distractors. **b** Main results of the memory task. Differences (Δ) between *d*’ (left), response times (middle) and confidence (right) for reliable minus unreliable prosodic archetypes. Data are presented as mean values with error bars showing the 95% confidence interval. Dots show individual data. Unreliable prosodies were memorized better and faster than reliable prosodies and were associated with more confident ratings. Black asterisks show the results of the two-sided paired *t* tests comparing reliable and unreliable prosodies, with **p = 0.01; *p < 0.05; *d*’: *p* = 0.01; response times: *p* = 0.026; confidence: *p* = 0.035. **c** Recency effect. Top: accuracy (top left) and confidence (top right) for reliable (light gray) and unreliable (dark gray) prosodic archetypes as a function of the position of the target within the audio stream. Bottom: differences between reliable minus reliable prosodic archetypes (black). There was no main interaction between position and prosody for accuracy, but the impact of prosody on confidence judgments interacted with target position such that recent unreliable targets lead to increased confidence. Data are presented as mean values with error bars showing the 95% confidence interval; *p < 0.05; **p = 0.01. Source data are provided as a Source data file.

To clarify the automaticity and cognitive depth at which this prosodic signature is processed, we ran a last study involving an implicit memorization paradigm. Forty participants (the same group of French speakers as Study 2) had to memorize pseudo-words that—unbeknown to them—had varying prosodies corresponding to the archetypes of doubtful, certain, lying, and honest prosodies that were found through reverse correlation in Study 1. Trials consisted of a sequence of six spoken words, followed by three pseudo-words presented on the screen. Participants had to recognize which of these three written pseudo-words had been presented in the preceding auditory sequence. As in Study 2, the prosody of each spoken pseudo-word was precisely manipulated in order to reproduce the signatures of honesty and certainty found in the first study (see “Methods” and Fig. 5a). Note that in order to measure implicit effects, Study 3 was actually conducted before Study 2, at a point where participants were not told about speech prosody or social attitudes at all. This procedure allowed us to test whether the prosodic signatures would be extracted by listeners, and automatically impact their verbal working memory despite the fact that they were task-irrelevant.

Two opposite predictions can be made as to how epistemic prosody may impact verbal working memory. On the one hand, it may be that listeners automatically filter out information if it is pronounced with an unreliable prosody: in this case, unreliable prosodies may impair memorization. On the other hand, it may be that unreliable prosodies function as attentional attractors ensuring the detection of deception: in this case, unreliable prosodies may boost memorization. Regardless of the direction of the effect, any found impact of the prosody of spoken words on memory would constitute evidence that an automatic extraction of the prosodic signatures has occurred.

To discriminate between these alternatives, a mixed hierarchical logistic regression was carried out to assess the impact of target position (1–6), prosody type (certain/doubt or honesty/lie), and prosody reliability (reliable or unreliable) on the accuracy (correct or incorrect response) of participants’ recall. There was a main effect of reliability on performances (X2 = 7.5, *b* = −0.05 +/− 0.02 sem, *z* = −2.7, *p* < 0.01), but, consistent with Study 1 and 2, no effect of type (X2 = 0.03, *p* > 0.8) and no interaction between type and reliability (X2 = 0.63, *p* > 0.4). This is consistent with our finding that a common prosodic signature supports social perceptions of both certainty and honesty. We therefore collated the data for certain/honest and lie/doubt for the rest of this analysis.

Overall, words pronounced with unreliable prosodies were memorized better than reliable ones (reliable: mean d’ = 1.68 +/− 0.46 SD, unreliable: *d*’ 1.8 +/− 0.46, *t*(39) = −2.7, *p* = 0.01, *d* = 0.26), and participants responded faster in this condition (reliable: mean RTs = 3.72 +/− 0.55 SD, unreliable: 3.67 +/− 0.54, *t*(39) = 2.31, *p* = 0.026, *d* = 0.1, see Fig. 5b). There was also a main effect of position on accuracy reflecting a typical recency effect (X2 = 653, *b* = 0.29 +/− 0.02 sem, *z* = 14.3, *p* < 0.0001), but this effect did not interact with reliability (i.e., reliability remained better for unreliable prosodies at every position, X2 = 1.2, *p* > 0.2, see Fig. 5c). Participants were also more confident in their memories when the target was pronounced with an unreliable as compared to a reliable prosody (reliable: mean confidence = 3.3 +/− 0.37 SD, unreliable: 3.34 +/− 0.34, *t*(39) = −2.18, *p* = 0.035, *d* = 0.11; there were no significant differences in metacognitive efficiency in between the two tasks, see supplementary information). Contrary to what was observed for accuracy, the effect of prosody on confidence interacted with the position of the target: a mixed hierarchical linear regression revealed both a main effect of reliability (X2 = 6.32, *b* = −0.02 +/− 0.009 sem, *t* = −2.1, *p* = 0.04) and position on confidence (X2 = 1090, *b* = 0.14 +/− 0.014 sem, *t* = 10.1, *p* < 0.0001) and an interaction between position and reliability (X2 = 4.55, *b* = 0.009 +/− 0.004 sem, *t* = 2.2, *p* = 0.033). Thus, when the target was present earlier in the stream, prosody boosted memorization but this beneficial effect remained inaccessible to metacognition (i.e., no change in confidence ratings). Given previous reports showing that performance but not confidence is impacted by unconsciously accumulated information^59^, this result suggests that the effect of prosody on recall in these trials resulted from unconscious influences.

Note that it is unlikely that this effect entails a social evaluation of the honesty or certainty of the speaker per se, since social attitudes were totally irrelevant in this task. Rather, the results suggest that the prosodic signature of unreliability automatically impacts verbal working memory, consistent with the idea that it reflects a rare regime of speech production that “pops-out” against typical (effortless, or neutral) prosody^14^, thereby attracting attention in an exogenous fashion. Further research testing whether this effect extends to non-speech sounds could establish the level of processing at which this acoustic signature is prioritized, and whether it occupies a dedicated niche in the acoustic landscape, and triggers increased alertness regardless of the type of sound that carries it, as recently shown for roughness^60^.

## Discussion

In four studies, we find converging evidence that a common, “core” signature supports social evaluations about the honesty and certainty of a speaker from speech prosody. The perceptual representations uncovered through reverse correlation for these two types of judgments are strikingly similar (Study 1): speakers are perceived to be unreliable (i.e., uncertain or dishonest) if they pronounce words with a rising intonation, less intensity at the beginning of the word, a slower speech rate, and more variable pitch and speech rate.

Crucially, listeners judged the same pairs of spoken pseudo-words similarly for both attitudes (Study 1), separate groups of listeners from various languages rated certain/honest and lying/doubtful archetypes similarly, these judgments were largely independent from listeners concepts about honest and certain prosodies (Study 2 and 3), and the core prosodic signature automatically impacted verbal working memory (Study 4). By testing the sharedness of these representations over social attitudes and languages, as well as their independence on conceptual knowledge and automaticity, we provide a direct empirical test of the hypothesis according to which this prosodic signature carries “natural” rather than culturally learned, language dependent “conventional” meaning^22,23^. As such, the results also question the assumption that social attitudes such as certainty should be contrasted with emotions in that—contrary to the latter—they primarily rely on controlled and culture dependent processes^29^.

An open question concerning this common prosodic signature is whether it is intrinsically communicative (i.e., a genuine signal that has evolved to affect receivers in particular ways, with receivers having evolved mechanisms to be affected by this very signal), or rather, constitute a natural sign or cue (i.e., a characteristic of the senders receivers can draw inferences from)^21,22^. The perceptual representations that we uncovered here with a data-driven method are strikingly similar to the actual consequences of cognitive effort on speech production^23,30^. Research has also shown that speakers produce this prosodic signature constitutively as a function of their certainty and competence, even in the absence of an audience^18,19^, which questions the view that it is fundamentally communicative^18,21^. Taken together, these findings suggest that, at a fundamental level, this prosodic signature constitutes a natural sign (or a cue) rather than a signal^22,61^: it can be interpreted by listeners, but its primary function is not to communicate. These displays may thus work like shivers, that are expressed naturally when one is cold, but can also be deliberately shown to ostensibly communicate that one is cold^22^.

In Study 4, we find that perceiving these displays automatically impacts working memory. Combined with the results obtained in Study 1, this shows that human listeners are equipped with mental representations allowing them to automatically detect prosodic signatures of unreliability that would be naturally produced when a speaker experiences more effort, thereby revealing a particularly adaptive mechanism on the side of receivers. An important open question for future research will be to understand the origin of this mechanism. It is possible that listeners learn what this prosodic signature naturally means by observing social partners, and registering associations between specific prosodic manifestations and speakers’ reliability, attested independently via reasoning, or by observing other signs of cognitive effort (e.g., facial expressions, gestures…). Alternatively, this signature may be part of a more ancient system shaped through evolutionary pressures. Future research involving pre-verbal infants and non-human primates could shed light on this issue. For instance, recent research suggests that toddlers already have biases to focus on similar prosodic markers in child-directed-speech and that this may be associated with better learning^62^.

Importantly, speakers can also manipulate these natural signs deliberately during communication. A prosodic display suggestive of reliability (e.g., falling pitch and volume, faster speech rate) can be observed when a cooperative speaker naturally displays it, or deliberately shows it to signal certainty or to persuade^14,16^, but it can also be faked by a deceitful speaker in a coercive way^2,21^. An important consequence of this flexibility is that there should be—in practice—no mandatory prosodic marker of dishonesty, as research analyzing liars’ speech indeed suggests^2,41^, and thus that perceiving a prosodic display suggestive of cognitive effort is not strictly conclusive in itself, as it is also crucial to determine whether the display was naturally or deliberately shown by the speaker^22^.

Our findings are also compatible with inferential (or pragmatic) views according to which evaluating the mental attitudes of others not only depends on perceptual representations, but also crucially, on additional inferential (mindreading) processes^22,63^, in particular when it is crucial to rely on contextual information and prior knowledge to infer speakers’ hidden intentions (i.e., when other information about the situation or the speaker suggest that dishonesty is probable)^2^. Although we find converging evidence for common representations at the perceptual level (similar reverse correlation kernels obtained in a forced choice procedure in Study 1, cross-validation in Study 2, similar implicit bias on memory for archetypes of doubtful and dishonesty prosodies in Study 3), we also find that judgments about (dis)honesty are less tied to sensory evidence than judgments about certainty. Listeners were less confident and precise in their decisions about (dis)honesty when no context allowed them to infer the speaker’s intention (Study 1), and important inter-individual differences were observed for (dis)honesty in a rating task that allowed participants to express individual decisional biases, and involved a context specifying that speakers’ were likely to be deceitful (Study 2A). Relatedly, a recent study found that listeners’ motivation to understand a message does not impact their perception of prosodic signatures of reliability per se, but influences how they exploit them to evaluate the speaker’s message^46^. Thus, like other types of vocal or facial expressions (e.g., smiles that can be interpreted as affiliatory or ironic)^63^, at a pragmatic level listeners interpret these prosodic signatures of reliability differently depending on the context, and individual factors. This is compatible with inferential models of non-verbal communication^22^, where it could be said that non-arbitrary (natural) signs (here of cognitive effort) have to be interpreted in a context dependent manner to infer speakers’ meanings. Such a framework emphasizes the continuity between non-verbal ostensive communication and linguistic communication, the crucial difference between them being that the latter relies on conventional symbols rather than natural signs^61^.

Research on deception detection has shown that observers are relatively poor at detecting liars explicitly in laboratory settings^2,40^. As mentioned above, this is partly explained by the fact that liars have a relative control over their displays, with the consequence that no behavioral cue to deceit is mandatory. Yet, it has also been suggested that there is a dissociation between explicit and implicit (or intuitive) abilities to detect lies^2,3^. While observers may be quite good at picking up relevant cues unconsciously (or intuitively), they may not always use them to overtly and explicitly report dishonesty, in particular if they assess that “the costs of failing to detect deception (are inferior to) those of signaling distrust”^3^, or if they have been taught to rely on cues that turn out to be unreliable such as gaze aversion^2^.

Consistent with these ideas, here we find that the prosodic signature that is relevant to detect the dishonesty of a speaker is extracted automatically (Study 4), and that percepts and concepts about dishonest speech prosody largely dissociate (Study 2B). Notably, in the first study where contextual and decisional factors were neutralized in a forced-choice procedure, listeners’ sensitivity to prosodic displays was highly significantly above chance in the honesty task, with a large effect size. Although not directly comparable, this contrasts with the medium to low effect sizes found in a meta-analysis compiling average levels of accuracy in explicit deception detection tasks (i.e., that reflect both observers’ sensitivity and their biases^40^). Finally, Study 2A shows that the ability to express individual decisional biases in a context where salient information about speakers’ intentions is available (e.g., that they are likely to be bluffing) sways listeners’ interpretation of the prosodic signature in one way or the other, while judgments about the certainty of the speaker remain strictly tied to sensory evidence. Thus, although listeners possess automatic mechanisms for detecting the prosodic signature of unreliability, they interpret this display differently depending on the context, individual factors, and, to a lesser extent, their concepts about epistemic prosody. A promising venue for future research will be to combine our data-driven psychophysical method with specific manipulations of the context known to modulate the cost of exposing deceit^3^, or the exploitation of prosodic information to evaluate a speaker’s message^46^, to examine whether—as suggested by Study 2—these factors impacts decisional biases and contextualized inferences rather than perceptual sensitivity to prosodic features. In addition, given that our method allows a very fine description of the representations used by listeners to detect unreliability, it will be interesting in the future to directly compare these representations with speech produced in various naturalistic conditions, to precisely examine the extent to which the perception and production of unreliable speech overlap, and assess how accurate and sensitive to the context listeners are.

Overall, we found weak links between perceptual and conceptual knowledge in our participants. Strikingly, the only acoustic dimension for which perceptual and conceptual levels seemed to relate was speech rate. Response times are known to be flexibly associated with certainty depending on the speed accuracy tradeoff^64^, and observers rely on their own experience with a task to map the latency of other agents’ responses to subjective metacognitive states^65^. Thus it may be that only those acoustic features that do not consistently vary with reliability, and therefore require more flexible assessments, are represented at the conceptual level. We also note that the loose association between percepts and concepts found here is in sharp contrast with recent studies in the visual domain suggesting a match between individuals’ conceptual knowledge about emotions, and their perceptual representations of facial expressions exposed with a similar reverse correlation approach^66^. This discrepancy could stem from the fact that we investigated attitudinal rather than emotional displays here, or from slight differences in the procedures used to evaluate participants’ conceptual knowledge; alternatively, it may reveal a genuine difference between auditory and visual modalities, a possibility worth investigating in further research.

In line with a bulk of data showing differences in how males and females process emotional and attitudinal prosody^20^ and differ regarding their levels of empathy^67^, and tendencies to lie^68^ and detect lies^69^, we found substantial gender differences in this study (see Figs. SIII and SVII.A). Interestingly, gender differences were limited to explicit judgments (Study 1 and 2) while at the implicit level there were no differences between males and females (Study 3). In the second study, females were more likely to interpret certain/honest prosodies as faked displays when the context suggested that speakers could be deceitful (i.e., bluffing). This is consistent with research showing that females engage further neural processing upon hearing conflicting signatures to speakers’ certainty^17^ and reports documenting gender differences in deception detection^69^. Whether the differences we observed here are linked to gender per se, rather than other related factors such as empathic or anxiety traits, remains unclear given recent results showing that empathy and anxiety mediate gender differences in the neural processing of prosodic signatures of certainty^20^. Studies involving bigger sample sizes and specifically designed to address this issue are needed to further understand how gender, other personal traits, as well as socialization and personal experiences, relate to the interpretation of (un)reliable prosodic displays. Relatedly, recent research involving twins showed that face impressions of trustworthiness are mostly determined by personal experiences rather than genes or shared environments^70^.

Here we could not examine how phrasal prosody and word meaning interact with the prosodic signature that we observed: because we were interested in uncovering generic and language independent effects, our procedure involved isolated pseudo-words. Yet, previous research based on naturalistic speech has shown that certainty is mostly reflected in the prosody used to pronounce target words (i.e., words concerned by the epistemic marking, generally at the end of the utterance^14^), and that social evaluations made on isolated words and sentences strongly correlate, and do not depend upon semantic content^71^, which suggests that our findings would generalize to real spoken words embedded in discourse. This being said, it would be interesting to use our method, which allows a very fine-grained description of listeners’ representations, to investigate how exactly phrasal and linguistic aspects of prosody interact with markers of reliability in speech. Another potential extension of this study would be to examine other acoustic features that have previously been associated with certainty and/or cognitive effort, such as measures of voice quality^14,30^.

A final open question concerns the finding that unreliable prosodies were memorized better than reliable ones in Study 3. This result shows that unreliable prosodies increase encoding in short-term memory, potentially through an alerting mechanism, in line with models suggesting that socially salient words should be encoded better because they capture attention^72^. Yet, although this privileged encoding of unreliable prosodies can be beneficial in the short term (e.g., enabling listeners to detect dishonesty), it would be rather detrimental in the long term (e.g., leading to memorizing unreliable information). Thus further research should test the impact of epistemic prosody on long-term retention, and its relation with the short-term, working memory effect that we observed here.

In conclusion, here we uncover a core, language-independent prosodic signature that acts as an attention-grabbing marker of (un)reliability, and on the basis of which various types of social evaluations, such as judgments about the certainty or honesty of a speaker, can be contextually constructed. Our results add to the growing body of evidence suggesting that, contrary to decades of research arguing that humans are highly gullible, dedicated mechanisms actually allow us to detect unreliability in our social partners efficiently^3,6^. Our findings disentangle the contribution of perception, concepts, and (meta)cognition to epistemic vigilance in the context of speech perception, and reveal that it also relies on implicit mechanisms that enable the quick and efficient detection of displays suggestive of unreliability, thereby complementing prevailing ideas advocated in the literature that emphasize the importance of reasoning and explicit aspects^1,6^. These findings also have the potential to lead to several applications, for instance, to investigate metacognition and selective learning in pre-verbal population^5,11^ or to develop light tools in the context of forensic practices^73^. More generally, our findings highlight the critical role played by audition for epistemic vigilance. This has important implications at the numeric age, where information heavily transits in written forms on the internet, and suggests that at least some of the tools enabling epistemic vigilance may not be adaptive anymore in the modern world^74^.

## Methods

### Study 1

#### Participants

Twenty (11 females, mean age = 22.6 years +/− 3.2) French listeners participated in the first study that included two testing sessions separated by 1 week. Sample size was determined a priori based on a recent study using the same methodology^26^. Participants were recruited via the INSEAD pool, and most of them were students, so the sample was not very diverse in terms of socio-economic background (socio-economic background was not systematically collected in this study, but participants were recruited from the same pool of participants as Study 2/4 so we can assume a similar distribution as the one we report below). All participants reported having no hearing impairments, they were native speakers of French. One participant had to be excluded because he did not attend the second session. In addition, three participants did not use the confidence scale reliably (i.e., two participants reported the same rating >75% of the time out of 100 possible values, and one participant used <10% of the scale, oscillating around the midpoint) and were therefore excluded for the analysis concerning confidence judgments (similar results were obtained in the full group regarding most analysis, except for the computation of metacognitive sensitivity since sigmoid curves for high- and low-confidence trials could not be reliably fitted for these outlying participants). Participants signed a consent form and were remunerated for their participation. Ethical approval was obtained, and experimental data were collected at INSEAD/ Sorbonne University Center for Behavioral Science.

#### Stimuli

Sounds were created with CLEESE, a voice transformation toolbox (https://forum.ircam.fr/projects/detail/cleese/) that allows creating random fluctuations around an audio file’s original contour of pitch, loudness, and duration^75^. Original tokens were 10 bi-syllabic pseudo-words obeying phonotactic regularities of French and using equally frequent syllables (*bazin*, *bivan*, *bodou*, *dadon*, *dejon, dobue*, *gibue*, *vagio*, *vevon*, *vizou*) produced by a male and a female French speaker; they therefore contained diverse phonetic contents that are representative of the phonotactic space of French, while remaining novel as they were created by combining phonemes into novel pseudo-word forms that do not correspond to a real word in French. Because this study focused on the perception of social attitudes rather than social traits or personality impressions, we used only two voices (a male and a female speaker). The pitch contour of initial recordings was artificially flattened and normalized for loudness and duration (480 ms). We then transformed the stimuli by randomly manipulating the pitch, loudness, and duration of the pseudo-words independently in four consecutive windows of 120 ms, by sampling five breaking points values on a normal distribution (pitch: SD = 100 cents, clipped at +/−2.2 SD, loudness: SD = 1.7 dB, clipped at 2.2 + /- SD, duration: SD = 1.25 stretching factor, clipped at +/−2.2 SD). These values were chosen so as to cover the range observed in naturally produced utterances^75^ and linearly interpolated between successive time points to ensure a naturally sounding transformation. Out of the 115 participants tested over the four studies, only 3 (including 1 German speaker who was an expert in voice synthesis tested in the third study) spontaneously reported that the voices were not natural exemplars but resynthesized voices during debriefing, stating, for instance, that the voices sounded “robotic.”

#### Procedure

The order of the two experiments (certainty or honesty) was counterbalanced across participants, and the directionality of the responses was inverted across tasks: participants had to judge which of the two voices was the most certain (confident) in one task, and which of the two voices seemed to be lying in the other task, in order to avoid contagion effects. In each session, participants listened to 880 pairs of randomly modulated voices. After hearing the two sounds sequentially, participants were asked to select one of the two stimuli. In the honesty task, participants were asked to select the stimuli in which the speaker seemed to be lying the most, while in the certainty task they were asked to select the stimuli in which the speaker seemed to be the most confident (i.e., certain). Ten percent of the stimuli were repeated twice to allow us to examine choice consistency (i.e., double pass consistency procedure^48,49^). Stimuli were presented in twenty blocks, each containing a different speaker and pseudo-word. The order of presentation of the blocks was pseudo-randomized for each participant so that blocks were grouped per speaker (e.g., the first ten blocks corresponded to the female or male speaker, with order counterbalanced across participants, and order of presentation of the ten blocks containing different pseudo-words randomized for each participant). After making a choice, participants reported their confidence on a scale from 1 to 4. In order to keep the participants motivated in this long task, a fake score (a random number taken from a uniform distribution ranging from 60 to 90%) supposed to reflect performances was displayed at the end of each block and participants were led to believe that financial compensation depended on their performances. The task was coded using Matlab.

#### Reverse correlation analysis

Three reverse correlation kernels were computed for each participant and each task by subtracting the mean pitch/loudness/duration of the spoken words classified as certain (i.e., selected) or honest (i.e., not selected) from the mean pitch/loudness/duration of the spoken words classified as doubtful (i.e., not selected) or lies (i.e., selected). Note that, because the instructions were counterbalanced in our two tasks (i.e., in the certainty task participants had to report which prosody was reliable, while in the honesty task participants had to report which prosody was unreliable), we reverse coded the values for the certainty task in order to allow direct comparisons between the two tasks (i.e., we subtracted the values for unchosen/honest voices from the values for chosen/lying voices). Individual kernels were normalized by dividing them by the sum of their absolute values^26^ and averaged for each task. This analysis was conducted directly on the five original breaking-point values used to transform the original stimuli for duration (the last acoustic transformation to be performed). For pitch and loudness, the original breaking-point values may not exactly correspond to what the listeners heard, because the audio manipulations were executed in a sequential order, so subsequent transformations may have slightly altered the transposition that was intended originally. We thus ran acoustic analysis in 12 consecutive windows to obtain fundamental frequencies and root mean square (RMS) values for each stimulus, in order to remain as close as possible to the pitch and loudness that were actually heard by the participants. The impact of segment and task were assessed for each acoustic dimension by running hierarchical linear mixed regressions including participant as a random factor with the lmerTest package in R^76^.

#### Percentage of agreement

Agreement across tasks was computed for each participant as the correspondence between judgments given in the honesty task and certainty task (e.g., agreement = 1 if for one pair of stimuli the participant perceived the same stimuli to be more certain and less dishonest). Agreement within task was computed for each participant by comparing responses given to repeated pairs of stimuli (e.g., agreement = 1 if for one pair of repeated stimuli the participant selected the same stimulus twice). Note that, although agreement can provide a coarse measure of choice consistency, it is prone to decision biases. To account for these biases, we also used these values to estimate participants’ internal noise in both tasks, following a procedure described below.

#### Confidence

Confidence ratings were *z*-scored and averaged separately for trials where participants agreed or disagreed across the two tasks (Fig. 2a) or for trials where participants agreed or disagreed within the same task (Fig. 2b).

#### Psychometric curves

Computation of summed area under the curve (sAUC). For each trial, the sAUC is the sum of the areas under the curves corresponding to the differences between the normalized (i.e., *z*-scored per participant) dynamic profiles of the first minus second stimuli, for each acoustic dimension (pitch, loudness, and duration). Summing up the values of the AUC obtained for the three acoustic dimensions gives us an estimate of the strength and directionality of the evidence that was available to the participant in each trial. Values for loudness (RMS) were reverse coded, because loudness varied in the opposite direction as compared to pitch and duration (see Fig. 1). Psychometric curves were then constructed for each participant and task by computing the response probability of choosing the first stimulus in four bins depending on sAUC values and fitting sigmoid functions to this data. Sensitivity was computed as the slope of individual psychometric curves. Individual psychometric curves were also constructed for each level of confidence separately (determined by a median split). Metacognitive sensitivity was then computed as the difference in the absolute values of the slopes of the psychometric curves computed for high- versus low-confidence trials^52^. Metacognitive efficiency was then computed by dividing metacognitive sensitivity by the absolute value of sensitivity^77^. Because there is no clear, a priori ground truth in this experiment, absolute values of sensitivity were used to estimate metacognitive sensitivity and efficiency, which allows to specifically estimate how confidence judgments tracked the precision of decisions, over and beyond the idiosyncrasies that were observed at the perceptual level (that are reflected in the polarity of the relationships between decisions and sensory evidence).

#### Internal noise

Following an established procedure^49,50^, we converted the percentages of agreement into values of internal noise using a computational SDT model with response bias and late additive noise. This model assumes that the internal representation of each stimulus, before the addition of internal noise, follows a normal distribution centered on zero. Each representation is then corrupted by internal noise, modeled as a Gaussian noise source with standard deviation *σ*. Thus the only thing that differs between repeated presentations of the same stimulus is the sample of noise, which is drawn randomly from the Gaussian distribution for each repetition. On each trial, the model selects the stimulus of the pair associated with the largest value but is allowed to favor one interval over the other, which is implemented by a response criterion *c* that can differ from zero. Different values of *σ* and *c* lead to different percentages of similar responses given to repeated presentations of the same stimulus (percentage of agreement; referred to as *pc_agree*) and to different probabilities to select the first interval over the second (referred to as *pc_int1*), respectively. For each trial *i*, we can define:

*s*_*i*_: the difference between the representation of the stimulus presented in the second interval subtracted from the representation of the stimulus presented in the first interval; *s*_*i*_ = *s_interval_2* − *s_interval_1*, and follows a Gaussian distribution with mean=0 and SD = 1; it is the same for the two repetitions, because the stimuli are identical.

*σ*_*ir*_: the difference between the noise sample added to the stimulus of the second interval subtracted from that of the first interval; *σ*_*ir*_ = *σ*_*r*_*_interval_2* − *σ*_*r*_*_interval_1*, and follows a Gaussian distribution with mean=0 and SD = *σ*; it is different between the two repetitions *r*_1_ and *r*_2_; we note *σ*_*i*1_ for the first repetition and *σ*_*i*2_ for the second repetition.

For each pair of stimuli and repetition, the SDT model with response bias *c* selects the stimulus presented in interval 1 if (*s*_*i*_ + *σ*_*ir*_) < *c*, and the stimulus presented in interval 2 otherwise. There is agreement between the two repetitions (i.e., *pc_agree* = 1) if ((*s*_*i*_ + *σ*_*i*1_) > *c* and (*s*_*i*_ + *σ*_*i*2_) > *c*) or if ((*s*_*i*_ + *σ*_*i*1_) < *c* and (*s*_*i*_ + *σ*_*i*2_) < *c*). We ran computational simulations based on this formula to assess *pc_agree* and *pc_int1* (averaged over the two repetitions) for different values of *σ* and *c*. A fitting procedure was then used to search for the specific values of *σ* and *c* that minimized the mean-square error between the values of *pc_agree* and *pc_int1* predicted by the above model and the empirical estimates of *pc_agree* and *pc_int1* observed in each task. Critically for the present study, this procedure allows controlling two aspects. First, it allows us to check that the observations made from the percentage of agreement are robust after accounting for interval response bias: the percentage of agreement does not return a transparent measure of consistency in a task where there is no ground truth, in particular because of potential response bias^19^ (e.g., imagine participants who would select response B after every trial: their percentage of agreement would be 100%, but this would be related to a strong decisional bias rather than choice consistency). In comparison, the internal noise values estimated with the SDT model accounting for response bias described above do return a transparent, readily interpretable image of individuals’ consistency. Second, consistency estimates previously reported in the psychophysical literature are most often expressed in terms of internal noise (in units of external noise), because it allows comparing tasks related to different stimuli dimensions and involving different difficulties. Thus converting percentage of agreement values into internal noise estimates also allows us to compare our values to these references. Only values that fell within the interpretable range [0.2; 5] returned by the SDT model^50^ were considered for analyses (which was the case for 18 out of the 19 participants).

### Study 2

#### Participants

A new group of 40 (21 females, mean age = 27 years +/− 4.7 SD) French listeners participated in Study 2. Sample size was determined a priori based on a recent study using a similar methodology^26^. Participants were recruited via the INSEAD pool; 32 out of the 40 participants were students, 4 were employees, and 4 were unemployed, so the sample was not very diverse in terms of socio-economic status. Participant’s family income was distributed as follows: <500 euros (*N* = 1), between 500 and 2000 euros (7), between 2000 and 5000 euros (*N* = 23), >5000 euros (*N* = 6), not reported (*N* = 3). All reported having no hearing impairments and were native speakers of French. These participants also participated in a speech production task whose results will be reported in a different article^18^. Participants signed a consent form and were remunerated for their participation. Ethical approval was obtained, and experimental data were collected at INSEAD/ Sorbonne University Center for Behavioral Science.

#### Stimuli

Stimuli were constructed from the same spoken pseudo-words used in Study 1, but this time specific acoustic manipulations (as opposed to the random manipulations used in the first experiment) were applied to the original stimuli. Precisely, we modified the pitch, loudness, and duration of the 16 original stimuli (8 pseudo-words and 2 speakers) so that they match the four archetypical dynamic filters derived from the reverse correlation study (certain, honest, doubt, lie). These modifications were applied with three different levels of gains, with the maximum level of gain chosen so that the maximum value of the kernel approximates the original value chosen as a maximum for the first study, ensuring that the manipulated voices would still sound natural to the listeners.

#### Procedure

Study 2A. Participants were separated into two subgroups and rated either the certainty of the speaker (*N* = 20) or the dishonesty of the speaker (*N* = 20) on a 7-point Likert scale (Study 2A). Participants heard each stimulus twice, resulting in 384 trials. The order of presentation of the stimuli was randomized in two blocks of 192 stimuli for each participant. The task was coded in Python with the psychopy toolbox^78^.

Study 2B: At the end of Study 2A, participants were asked 12 questions regarding their concepts about lying and doubtful prosodies in a directed debriefing session. These questions were specifically designed to probe participants concepts about how certainty and honesty affect speech prosody for the three acoustic dimensions examined in this study (pitch, duration, and loudness) and concerned static (according to you, someone who is certain/lying will speak with a high/low pitch, high/low volume, fast/slow speech rate?) or dynamic (according to you, someone who is certain/lying will speak with a falling/rising intonation, increasing/decreasing loudness, accelerating/decelerating speech rate?) aspects. The order of the questions was counterbalanced across participants.

#### Analysis

Participants’ ratings were *z*-scored before being averaged for each level of gain in each task. Ratings were also split depending on participants’ responses to conceptual knowledge questions. The percentage of agreement for the six questions regarding concepts about certain and lying prosodies was computed by reverse coding the responses given for lie to allow direct comparisons between the two attitudes. Conceptual distance (Fig. SVII.C) was computed for each participant as the average agreement for responses given to the questions concerning knowledge about certain and lying prosodies. Perceptual distance was computed for each participant as the average of the absolute differences between ratings given for certain/honest versus ratings given for doubtful/lying prosodies in the listening test.

### Study 3

#### Participants

Two separate groups of native English (*N* = 22, 10 females, mean age = 29 +/− 9.55 years) and Spanish (*N* = 21, 12 females, mean age = 33.7 +/− 8.39 years) speakers with no exposure to French were tested on the same certainty task as Study 2. We also tested a third group of 12 speakers of various native languages (4 females, mean age = 26.7 +/− 5.55 years) who had a limited exposure to French (and English) on the same certainty task (instructions were in English): two German speakers (males), two English speakers (1 male), one Russian speaker (male), one Marathi speaker (male), one Spanish speakers (female), one Polish speaker (female), one Japanese speaker (male), one Swedish speaker (male), one Dutch speaker (male), and one Mandarin Chinese speaker (female). The group of multi-language speakers was recruited via the INSEAD pool, English speakers via the pool of University College London (London, United Kingdom), and Spanish speakers via the pool of the Universidad Del Desarrollo (Santiago, Chile); all but 2 of the 22 native English speakers were students, all but 2 of the 21 native Spanish speakers were students, and 5 of the 12 multi-language speakers were students (1 was unemployed, 6 were employees), so the sample was not very diverse in terms of socio-economic status. Participants’ level of comprehension of French was assessed through self-reports and an objective comprehension test in order to assess the impact of language proficiency on their perception of epistemic prosody. Self-reports consisted of three scales on which participants had to report (1) their general level of comprehension of spoken French, (2) their ability to have a basic conversation, and (3) their ability to perceive French intonations, from scales ranging from 0 (nothing at all) to 6 (native). The objective test involved five four-alternative forced choices (https://www.ciep.fr/tcf-tout-public/epreuves-obligatoires-comprehension-orale) allowing us to compute an objective score of comprehension. Participants reported very limited understanding of spoken French (*M* = 0.91 +/− 1 SD), a low ability to have a basic conversation (*M* = 1.08 +/− 1.11 SD), and a relatively low understanding of French intonation (*M* = 1.42 +/− 0.86 SD). Participants signed a consent form and were remunerated for their participation. Ethical approval was obtained at INSEAD/Sorbonne University Center for Behavioral Science. Speakers of the multi-language group were tested at INSEAD or IRCAM (Paris, FR). English speakers were tested at University College London (UK) and Spanish speakers at the Universidad Del Desarrollo (Santiago, Chile).

#### Procedure and stimuli

These were the same as in the certainty task of Study 2, the only difference being that the instructions were translated in their respective native languages for the group of English and Spanish speakers and in English for the group of multi-language speakers.

### Study 4

#### Participants

Participants in Study 3 were the same group of 40 participants as in Study 2.

#### Stimuli

Stimuli were a subset of the sample used for Study 2. There were 64 archetypal stimuli (corresponding to 8 pseudo-words, 4 prosody types, and 2 speakers) with an intermediate level of gain.

#### Procedure

Participants heard 6 pseudo-words separated by 1-s inter-stimulus intervals while fixating a cross in the middle of the screen. They then saw three alternatives appear on the screen: a target pseudo-word, which was present in the stream, and whose position and prosody was fixed, and two distractors that did not appear in the stream. Participants had to indicate which word they thought was present in the stream by pressing an arrow on the keyboard (left, down for middle, or right), before reporting their confidence on a scale from 1 to 4. The archetypal prosody of the target (certain/honest/lie/doubt) and the position of the target in the audio stream (1–6) were randomized with a Latin square for each participant and each stimulus type (8 pseudo-words and 2 speakers), resulting in 384 trials. Distractors were two alternative pseudo-words that were not present in the audio stream and had random prosodies taken from the same pool of stimuli. The spatial position of the target on the response screen (left/middle/right) was randomized at each trial. The task was coded in Python with the psychopy toolbox.

#### Analysis

Outlying values for response times (i.e., response times exceeding the third quartile +1.5 interquartile range) were excluded. This resulted in the exclusion of 4.6% of the trials and did not change the main results concerning accuracy and confidence but revealed an effect of reliability on response times that was not visible before pre-processing. Accuracy, response times, and confidence were averaged for each participant, position in the stream, and prosodic type (reliable: certain/honest versus unreliable: lie/doubt). Values obtained for unreliable prosodies were then subtracted from the values obtained for reliable prosodies.

### Reporting summary

Further information on research design is available in the Nature Research Reporting Summary linked to this article.

## Supplementary information

Peer Review File

Reporting Summary

Supplementary Information

## Data Availability

Additional raw data are available on the Open Science Framework via this link: https://osf.io/upkzy/?view_only=ceb3ba0500d74cf3a3c42d9a31fb0d91. Source data are provided with this paper.

## Source data

Source Data

## Code availability

All analysis scripts are available on the Open Science Framework via this link: https://osf.io/upkzy/?view_only=ceb3ba0500d74cf3a3c42d9a31fb0d91. The C.L.E.E.S.E. software used to modify vocal recordings is available at: https://forum.ircam.fr/projects/detail/cleese/.
