# Publisher Correction: Listeners’ perceptions of the certainty and honesty of a speaker are associated with a common prosodic signature

**DOI:** 10.1038/s41467-021-25958-w

**Published:** 2021-09-27

**Authors:** Louise Goupil, Emmanuel Ponsot, Daniel Richardson, Gabriel Reyes, Jean-Julien Aucouturier

**Affiliations:** 1 STMS UMR 9912 (CNRS/IRCAM/SU), Paris, France; 2 University of East London, London, UK; 3 Laboratoire des Systèmes Perceptifs, Département d’Études Cognitives, École Normale Supérieure, PSL University, CNRS, Paris, France; 4 Hearing Technology - WAVES, Department of Information Technology, Ghent University, Ghent, Belgium; 5 University College London, London, UK; 6 Universidad del Desarrollo, Santiago, Chile; 7 FEMTO-ST (FEMTO-ST UMR 6174, CNRS/UBFC/ENSMM/UTBM, Besançon, France

**Keywords:** Cognitive neuroscience, Human behaviour

Correction to: *Nature Communications* 10.1038/s41467-020-20649-4, published online 8 February 2021.

The original version of the Supplementary Information associated with this Article contained errors in Supplementary Figures 1, 3, 4, 5, 7 and 8 and an error in the figure legend of Supplementary Figure 8. The HTML has been updated to include a corrected version of the Supplementary Information; the original incorrect version of the Supplementary Information file can be found as Supplementary Information associated with this Correction.

## Supplementary information

Correct Supplementary information

Incorrect Supplementary information

