# Supplementary material for: Listeners’ perceptions of the certainty and honesty of a speaker are associated with a common prosodic signature
Source: Nat Commun. 2021 Feb 8;12:861. doi: 10.1038/s41467-020-20649-4 (PMC7870677; doi:10.1038/s41467-020-20649-4)
Supplement: Supplementary file 2 — Reporting Summary [file 41467_2020_20649_MOESM2_ESM.pdf]

nature research

Corresponding author(s): Louise Goupil

Last updated by author(s): Sep 23, 2020

Reporting Summary

Nature Research wishes to improve the reproducibility of the work that we publish. This form provides structure for consistency and transparency in reporting. For further information on Nature Research policies, see our Editorial Policies and the Editorial Policy Checklist.

Statistics

For all statistical analyses, confirm that the following items are present in the figure legend, table legend, main text, or Methods section.

n/a	Confirmed	
☐	☒	The exact sample size (*n*) for each experimental group/condition, given as a discrete number and unit of measurement
☐	☒	A statement on whether measurements were taken from distinct samples or whether the same sample was measured repeatedly
☐	☒	The statistical test(s) used AND whether they are one- or two-sided<br>*Only common tests should be described solely by name; describe more complex techniques in the Methods section.*
☐	☒	A description of all covariates tested
☐	☒	A description of any assumptions or corrections, such as tests of normality and adjustment for multiple comparisons
☐	☒	A full description of the statistical parameters including central tendency (e.g. means) or other basic estimates (e.g. regression coefficient) AND variation (e.g. standard deviation) or associated estimates of uncertainty (e.g. confidence intervals)
☐	☒	For null hypothesis testing, the test statistic (e.g. *F*, *t*, *r*) with confidence intervals, effect sizes, degrees of freedom and *P* value noted<br>*Give P values as exact values whenever suitable.*
☒	☐	For Bayesian analysis, information on the choice of priors and Markov chain Monte Carlo settings
☐	☒	For hierarchical and complex designs, identification of the appropriate level for tests and full reporting of outcomes
☐	☒	Estimates of effect sizes (e.g. Cohen's *d*, Pearson's *r*), indicating how they were calculated

Our web collection on statistics for biologists contains articles on many of the points above.

Software and code

Policy information about availability of computer code

Data collection	Data were collected using the psychotoolbox in Python (v3.7.3).
Data analysis	Data were analyzed in Python (v3.7.3) using the statsmodel, seaborn and matplotlib toolboxes, and statistics were conducted in R (v3.3.1) using the lmerTest package for mixed regressions analysis. Meta-d' was computed in Matlab (R2016a) with the Hmeta toolbox. Vocal recordings were transformed using C.L.E.E.S.E (v1) running in Matlab (R2016a). An open access version of this software has since then been developed in Python, and can be found here: https://forum.ircam.fr/projects/detail/cleese/.

For manuscripts utilizing custom algorithms or software that are central to the research but not yet described in published literature, software must be made available to editors and reviewers. We strongly encourage code deposition in a community repository (e.g. GitHub). See the Nature Research guidelines for submitting code & software for further information.

Data

Policy information about availability of data

All manuscripts must include a data availability statement. This statement should provide the following information, where applicable:

- Accession codes, unique identifiers, or web links for publicly available datasets
- A list of figures that have associated raw data
- A description of any restrictions on data availability

All data and scripts are available on the Open Science Framework: https://osf.io/upkzy/?view_only=ceb3ba0500d74cf3a3c42d9a31fb0d91.

Field-specific reporting

Please select the one below that is the best fit for your research. If you are not sure, read the appropriate sections before making your selection.

☐ Life sciences ☒ Behavioural & social sciences ☐ Ecological, evolutionary & environmental sciences

For a reference copy of the document with all sections, see nature.com/documents/nr-reporting-summary-flat.pdf

Behavioural & social sciences study design

All studies must disclose on these points even when the disclosure is negative.

Study description	The study involves four behavioral experiments where quantitative data were collected. In the first experiment, a reverse correlation procedure was used to derived listeners mental representations about reliable prosodies. In the second experiment, an independent group of listeners rated archetypal stimuli derived from the first experiment, and the relationship between perception of these archetypes, and conceptual knowledge about prosody was assessed. In the third experiment, a separate group of listeners from several languages were tested to assess the cross-linguistic aspect of the findings. In the last experiment, listeners (the same sub-group as in the second experiment) had to memorize words and recall them, to assess the impact of archetypal reliable/unreliable prosodies on verbal working memory.
Research sample	The research sample includes a total of 115 listeners. In the first experiment, we tested twenty (eleven females, mean age = 22.6 years +/- 3.2) French listeners. In the second experiment, we tested forty (21 females, mean age = 27 years +/- 4.7 SD) French listeners. Experiment 3 included a group of 22 native English listeners (10 females, mean age = 29 +/- 9.55), a group of 21 Spanish (12 females, mean age = 33.7 +/- 8.39) listeners with no exposure to French, and a separate multi-language group of 12 listeners of various native languages (4 females, mean age = 26.7 +/- 5.55) who had a limited exposure to French or English. The fourth experiment included the same 40 French listeners who participated in study 2.<br><br>The sample size of 20 participants per experimental group was chosen based on previous studies using a similar reverse correlation and listening tests methodology, that indicated that sufficient power can be achieved with this sample size (Ponsot et al., 2018, PNAS; Ponsot et al., 2018, JASA).<br><br>The sample included participants of various socio-economic and linguistic backgrounds, but the sample was not representative of the general population as a majority of them were students (at the Sorbonne in Paris, UCL in London or UDD in Chile). Study 2 and 4: 32 out of the 40 participants were students, 4 were employees and 4 were unemployed. They were from relatively healthy economic background, with 8 out of 40 participants reporting a household income below the national median. More precisely, participant's family income was distributed as follows: less than 500 euros (N = 1), between 500 and 2000 euros (7), between 2000 and 5000 (N = 23), above 5000 (N=6), not reported (N=3). Study 1: socio-economic backgrounds were not systematically collected for this first experiment, but participants were recruited from the same pool of participants as study 2/4, so we can assume an equivalent socio-economic background in this group. Study 3: all but 2 of the 22 native English speakers were students, and all but 2 of the 21 native Spanish speakers were students. In the multi-language group, 5 of the 12 participants were students (1 was unemployed, 6 were employees).
Sampling strategy	Sample sizes for the three studies were determined a priori based on previous research using similar methodologies (Ponsot et al., 2018). Participants were randomly assigned to experimental groups in study 1 and study 2.
Data collection	Data collection was performed by research assistants or students (Louise Vasa, Gabriel Vogel, Lou Seropian, Mauricio S. Barrientos Alvarez, and Martin A. Castro Yanez, all acknowledged in the paper) who were totally blind regarding the purpose of the study and experimental conditions (stimuli were randomly sampled for each participant). The first author collected the data for the group of English participants tested at UCL. Data were acquired on a computer.
Timing	Data for the first study were acquired between the 11th and 20th of July 2017. Data for the second study were acquired between the 7th and 20th of June 2018 (for the French listeners), between the 10th and 11th of December 2019 for the group of English listeners, between the 8th of November and the 21st of December 2019 for the group of Spanish listeners; and on the 23rd of July for the group of 12 multi-language listeners. Data for the third study were acquired between the 7th and 20th of June 2018.
Data exclusions	Study 1. One participant had to be excluded because he did not return for the second session of the study. Nineteen participants were thus included regarding the main analysis. Three participants did not use the confidence scale (they reported the same rating more than 75% of the time out of 100 possible values, or used less than 50% of the confidence scale, these exclusion criteria were used based on Folke et al., 2016) and had to be excluded from the analysis regarding self-confidence, as stated in the manuscript. Study 2: no data were excluded. Study 3. Outlying values for response times (i.e., response times exceeding the third quartile +1.5 interquartile range) were excluded. This resulted in the exclusion of 4.6 % of the trials, and did not change the main results concerning accuracy and confidence, but revealed an effect of reliability on response times that was masked before pre-processing due to outlying values.
Non-participation	One participant did not return for the second session of experiment 1, and had to be excluded from the sample. Three participants in experiment three did not return the questionaires allowing to assess conceptual knowledge and couldn't be included in this analysis.
Randomization	Participants were randomly assigned to experimental groups. Stimuli were presented in pseudo-randomized or fully randomized orders (specified in the corresponding methods sections).

Reporting for specific materials, systems and methods

We require information from authors about some types of materials, experimental systems and methods used in many studies. Here, indicate whether each material, system or method listed is relevant to your study. If you are not sure if a list item applies to your research, read the appropriate section before selecting a response.

Materials & experimental systems

n/a	Involved in the study	
☒	☐	Antibodies
☒	☐	Eukaryotic cell lines
☒	☐	Palaeontology and archaeology
☒	☐	Animals and other organisms
☐	☒	Human research participants
☒	☐	Clinical data
☒	☐	Dual use research of concern

Methods

n/a	Involved in the study	
☒	☐	ChIP-seq
☒	☐	Flow cytometry
☒	☐	MRI-based neuroimaging

Human research participants

Policy information about studies involving human research participants

Population characteristics	See above
Recruitment	French participants, and multi-language participants exposed to French were recruited via the INSEAD plateform, and only included gender (to balance the number of females/males) and no hearing/visual impairments as an inclusion criteria. Similarly, English listeners and Spanish listeners were recruited via the plateforms of UCL and UDD, with the same inclusion criteria.
Ethics oversight	The INSEAD/ Sorbonne University Center for Behavioral Science approved the protocol for all four studies.

Note that full information on the approval of the study protocol must also be provided in the manuscript.
