# Supplementary material for: Listeners’ perceptions of the certainty and honesty of a speaker are associated with a common prosodic signature
Source: Nat Commun. 2021 Feb 8;12:861. doi: 10.1038/s41467-020-20649-4 (PMC7870677; doi:10.1038/s41467-020-20649-4)
Supplement: Supplementary file 3 — Supplementary Information [file 41467_2020_20649_MOESM3_ESM.pdf]

Supplementary Information

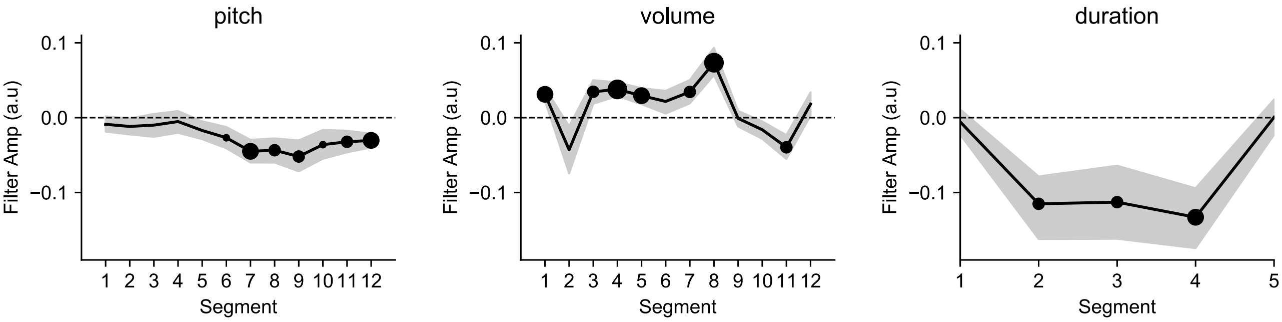

Figure SI. Aggregated reverse correlation kernels (study 1). Normalized kernels derived from the reverse correlation analyses when combining both tasks. Filter amplitudes correspond to the values obtained for each participant, acoustic dimension and segment by subtracting the average (pitch, loudness and duration) values obtained for stimuli judged as certain or honest from the values averaged for the unchosen stimuli, and normalizing these values for each participant by dividing them by the sum of their absolute values. Significant deviations from zero (one-sample t-tests) are indicated at the corresponding segment positions with circles, with increasing sizes corresponding to $p < 0.1$; $p < 0.05$; $p < 0.01$ and $p < 0.001$ (p-values per segment for pitch: 0.4, 0.29, 0.53, 0.75, 0.19, 0.08, 0.006, 0.01, 0.02, 0.07, 0.03, 0.004); loudness: 0.001, 0.18, 0.048, 0.0002, 0.009, 0.14, 0.03, 0.0004, 0.93, 0.18, 0.02, 0.28; duration: 0.75, 0.01, 0.04, 0.003, 0.98). Shaded areas show SEM. Source data are provided as a Source Data file.

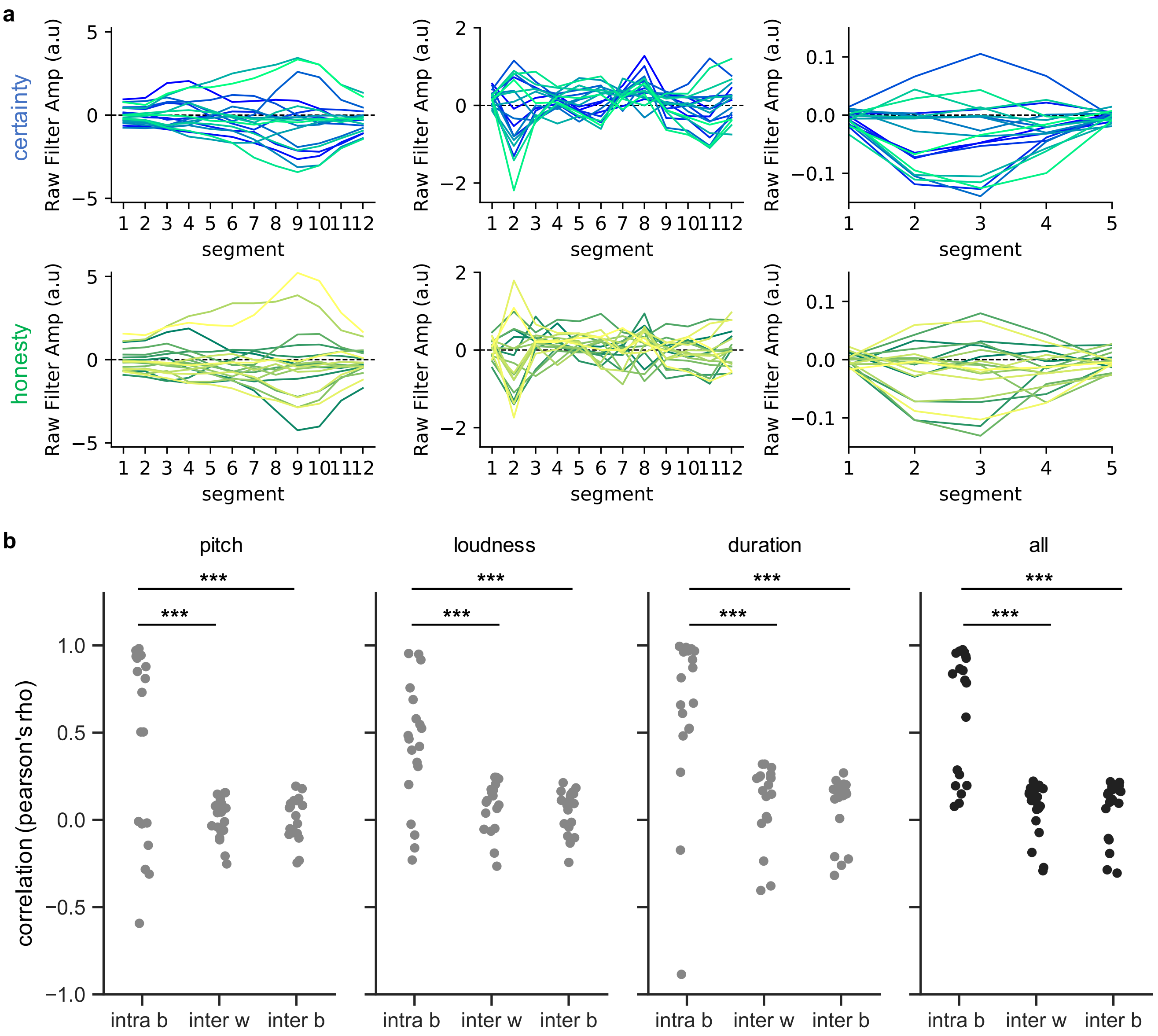

Figure SII. Between-task intra-individual consistency despite inter-individual differences (study 1). A) Individual raw reverse correlation kernels in the two tasks (top, blue: certainty; bottom, green: honesty) across the three acoustic dimensions (pitch / left, loudness / middle, duration / right). Each individual is depicted by a different shade. This figure shows non-negligible inter-individual differences. **B)** Comparison of the correlations between the kernels from the same individuals between both tasks (intra. b) with the correlations between the kernels from different individuals either computed within the same task (inter. w) or between both tasks (inter. b). Whether these analyses are conducted for the different dimensions separately (left panels, grey) or pooled together (right panel, black), they clearly show intra-individual consistency between both tasks, despite the non-negligible inter-individual differences. Dots show individual data; black asterisks represent the significance of two-sided post-hoc Tukey HSD with Bonferroni correction comparing the three types of correlations, with *** representing $p < 0.001$ (pitch: intra b vs. inter b $p = 0.00005$, intra b vs. inter w $p = 0.00005$; all other p-values < 0.00001). Source data are provided as a Source Data file.

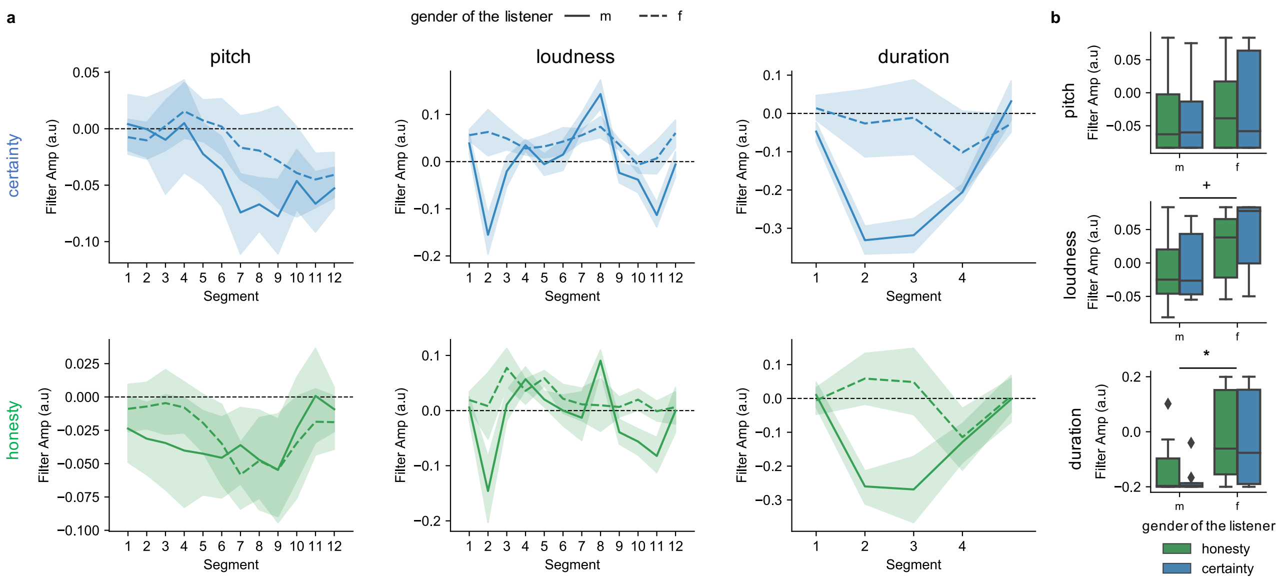

Figure SIII. Reverse correlation results depending on listeners' gender (study 1). A) Dynamic effects. Normalized reverse-correlation kernels in the two tasks (blue: certainty; green: honesty) depending on listeners' gender (males: solid lines; females: dotted lines). Male and female listeners used acoustic dimensions in a slightly different fashion. Female listeners did not rely on duration consistently, as shown by the fact that group-level kernels were flat for this dimension (interaction between segment and gender: F(4,68) = 2.98, p = 0.025 in the certainty task ; F(4,68) = 3.57, p = 0.01 in the honesty task). An inspection of individual data suggests that this was due to idiosyncrasies in using duration rather than female listeners not relying on duration at all: some (N = 4) female listeners tended to judge slower prosodies as more reliable, while other (N = 7) female listeners behaved like male listeners (judging faster prosodies to be more reliable). Regarding loudness, there was also a significant interaction between segment and gender (F(11,187) = 4.7, p < 0.001 in the certainty task ; F(4,68) = 1.85, p = 0.05), reflecting the fact that the filters were less dynamic for female listeners: they perceived louder voices as more certain and honest (i.e., filters were shifted upwards, but flatter). Regarding pitch, there was no interaction between gender and segment for pitch kernels in none of the two tasks (certainty: F(11,187) = 0.5, p = 0.9; honesty: F(11,187) = 0.36, p = 0.97), suggesting that both male and female listeners used intonation similarly. **B) Static effects.** The same data averaged across segment. Box plots show the quartiles of the dataset, with the whiskers extending to show the rest of the distribution, with the exception of outliers (i.e., data points that fall beyond 1.5 times the inter-quartile range) that are shown by diamonds. There was a main effect of gender on the amplitude of the kernels for duration (F(1,17) = 5.6, p = 0.03, represented by *), a marginal effect for loudness (F(1,17) = 3.6, p = 0.076, represented by +), and no effect for pitch (F(1,17) = 0.2, p = 0.66). Strikingly, although there were differences in how female and male listeners used prosodic dimensions to categorize the stimuli, these variations were reflected similarly in both tasks: there were no interactions between gender and task for any of the three dimensions (pitch: F(1,17) = 0.7, p = 0.41; loudness: F(1,17) = 0.18, p = 0.68; duration: F(1,17) = 0.05, p = 0.82). This is in favor of the hypothesis that listeners – despite some idiosyncratic behaviors in how they rely on specific acoustic dimensions - represent certain and honest prosodies similarly. Source data are provided as a Source Data file.

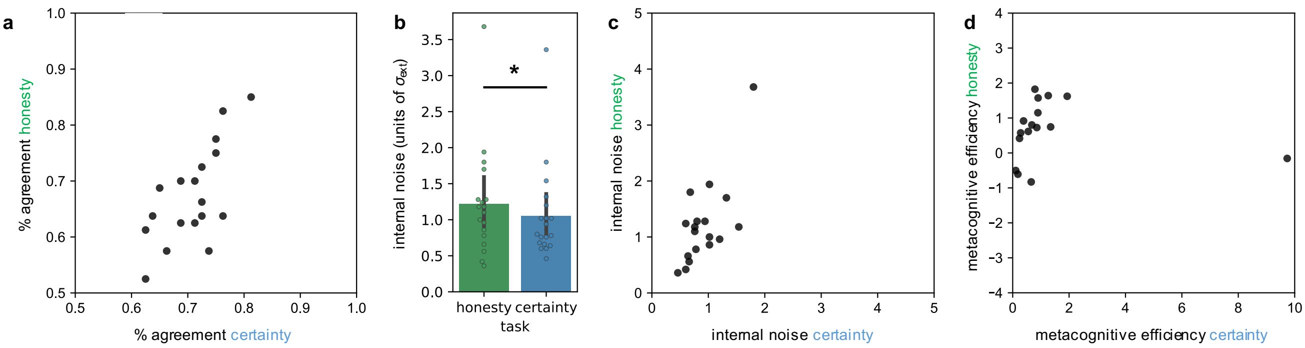

Figure SIV. Percentage of agreement and internal noise within each task (study 1). A) Percentage of agreement between responses given for trials that were presented twice (each dot corresponds to one participant). Agreement was significantly higher for certainty than honesty (t(18) = 2.50, p = 0.022) and was significantly correlated between the two

tasks (Spearman – rho(18) = 0.69, p = 0.001). **B) Internal noise** values inferred in both tasks (N = 18; only one out of nineteen listeners did not fall in the interpretable range, and was thus removed from the analysis; see methods). Bar plots show the mean with the 95% confidence intervals, and dots show individual data. Internal noise values (M±SD: honesty: 1.2 ± 0.76; certainty: 0.92 ±0.35) were within the typical range observed in most low-level psychophysical tasks, and consistent with what was recently observed in a similar high-level cognitive auditory tasks[1]. Internal noise was lower for certainty as compared to honesty (* represents the result of a two-sided paired t-test comparing the two tasks, t(17) = 2.23, p = 0.039, d = 0.25), and **C)** these values were correlated between both tasks (Spearman – rho(17) = 0.55, p = 0.019). **D)** Correlation between metacognitive efficiency computed separately in the certainty and honesty task for each participant. Despite the presence of an outlier (one participant had a really high score in the certainty task, but was at zero in the honesty task), there was a significant correlation between metacognitive efficiency in the two tasks (Spearman's rho(15) = 0.53, p = 0.034). Source data are provided as a Source Data file.

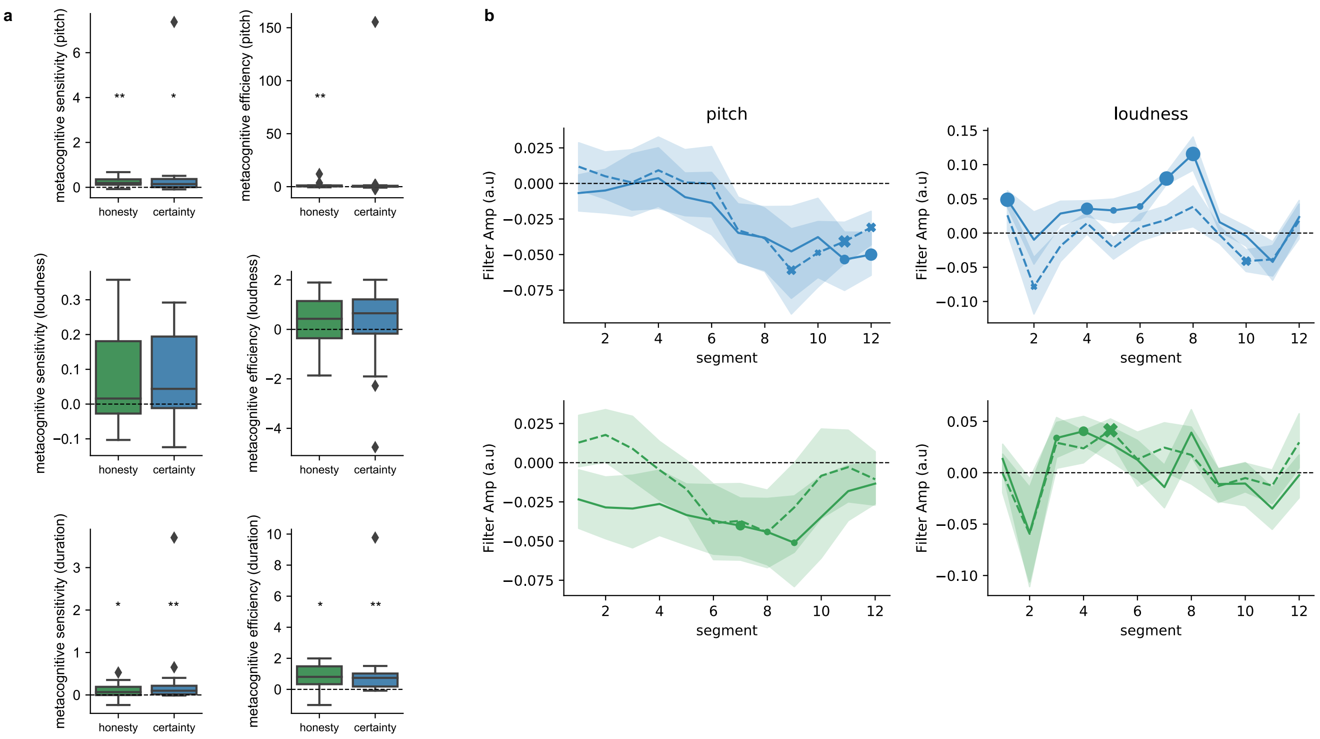

Figure SV. A) Metacognitive sensitivity and efficiency computed separately for pitch, loudness and duration (study 1). Metacognitive sensitivity (computed by subtracting slopes of the psychometric functions constructed from high confidence responses from the slopes constructed from low confidence responses) was significantly above chance in both tasks (blue certainty; green honesty) for pitch (certainty: Z(15) = 21, p = 0.015, d = 0.34; honesty: Z(15) = 7, p = 0.0016, d = 1.13) and duration (certainty: Z(15) = 6, p = 0.0013, d = 0.41; honesty: Z(15) = 26, p = 0.03, d = 0.6), but not significantly so for loudness (certainty: Z(15) = 32, p = 0.06, d = 0.57; honesty: Z(15) = 42, p = 0.18, d = 0.54). Differences between tasks were not significant (pitch: p = 0.5; loudness: p = 1; duration: p = 0.53). Metacognitive efficiency (metacognitive sensitivity divided by sensitivity) was above chance for duration in both tasks (certainty: Z(15) = 6, p = 0.0013, d = 0.53; honesty: Z(15) = 25, p = 0.026, d = 0.72), and for pitch in the honesty task (Z(15) = 10, p = 0.003, d = 0.5), but not in the certainty task (Z(15) = 39, p = 0.13, d = 0.26). It did not significantly differ from chance for loudness (certainty: p > 0.3; honesty: p > 0.4). Differences between tasks were not significant (pitch: p = 0.72; loudness: p = 0.84; duration: p = 0.3). Finally, sensitivity (X2 = 4.505, p > 0.10), metacognitive sensitivity (X2 = 2.9734, p > 0.2) and efficiency (X2 = 2.2607, p > 0.3) did not significantly vary depending on acoustic dimensions in a linear mixed regression. Still, the results suggest that pitch and duration impacted confidence, while loudness did not, which may be due to the fact that this analysis does not account for dynamic effects. Box plots show the quartiles of the distribution, with the whiskers extending to show the rest of the distribution with the exception of outliers (i.e., data points that fall beyond 1.5 times the inter-quartile range) that are shown by diamonds. Black asterisks show the significance of one-sample Wilcoxon signed-ranked tests against zero, with *: p < 0.05; **: p < 0.01. **B) Reverse correlation kernels depending on confidence.** To account for dynamic effects, we also computed reverse correlation kernels separately for high (solid lines) versus low confidence (dotted lines) judgments for pitch and loudness (the two acoustic dimensions that critically varied across time) in each task (blue certainty; green honesty). A linear mixed regression was then conducted to assess the impact of confidence level, segment and task on the kernels. As reported in the main text, there was a main effect of segment for pitch (linear) and loudness (quadratic), no effect of task and no interaction between segment and task. Entering confidence

level in the model revealed a main effect of confidence level for pitch (X2 = 4.33, p = 0.037, t = -2.22, beta = -0.011 +/- 0.005 sem) reflecting the fact that pitch was lower for confident responses overall (there was no interaction between confidence level and segment nor task). For loudness, there was no significant main effect of confidence level (X2 = 2.6, p = 0.1), but a significant interaction between confidence level and task (X2 = 5.7, p = 0.017, t = 2.55, beta = 0.02 +/- 0.008 sem), reflecting the fact that loudness increased with confidence in the certainty but not the honesty task. Significant deviations from chance are indicated at the corresponding segment positions by circles (high confidence) or crosses (low confidence), with increasing sizes corresponding to p < 0.1; p < 0.05; p < 0.01 and p < 0.001. Shaded areas show SEM. Source data and exact p-values for Figure SVb are provided as a Source Data file.

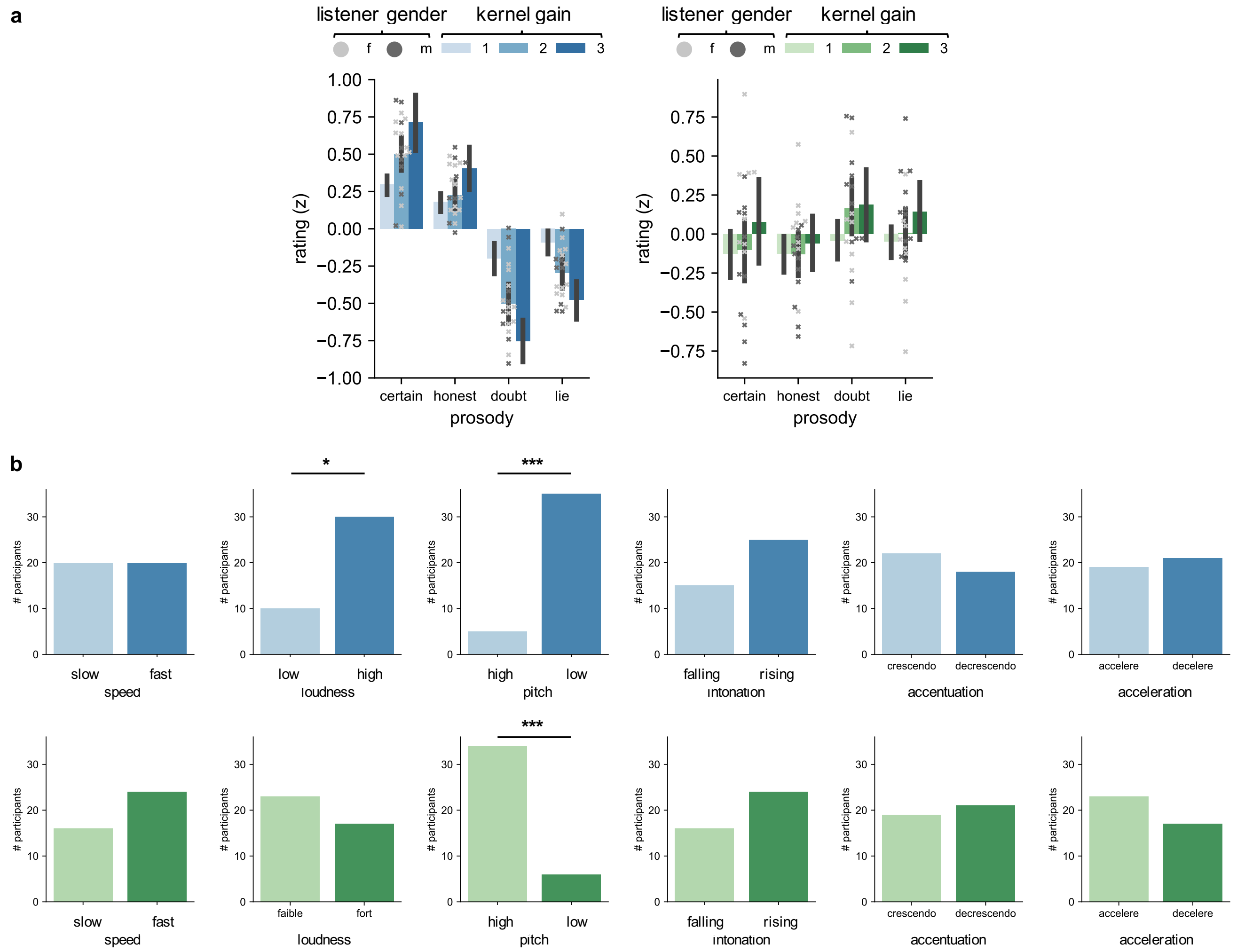

Figure SVI (study 2). A) Group level results depending on gain in the two tasks. Normalized (z-scored) ratings in the certainty (left, blue; N = 20) and honesty (right, green; N = 20) tasks depending on the gain of the acoustic transformations (represented by the three levels of shades). Bars show the mean with 95% confidence intervals, and crosses represent individual data for female (grey) and male participants (black), averaged across all levels of gain. In the confidence task, both honest and confident prosodies were judged as more confident than doubtful and lying prosodies. In the honesty task, greater inter-individual differences were observed. Certain/honest prosodies were perceived as more certain than lying prosodies (post-hoc Tukey HSD with Bonferroni correction, gain 1: p < 0. 00001/0.00001, d = 2.52/1.81; gain 2: p < 0. 00001/0.00001, d = 3.23/2.45; gain 3: p < 0.00001/0.00001, d = 3.3/2.96) and doubtful prosodies (gain 1: p < 0.00001/0.00001, d = 2.59/2; gain 2: p < 0.00001/0.00001, d = 3.61/3.92; gain 3: p < 0.00001/0.00001, d = 3.93/3.71) for each level of gain. In the honesty task, doubtful prosodies were perceived as more dishonest than honest prosodies for intermediate (p = 0.0001, d = 0.83) and high levels of gain (p = 0.003, d = 0.54), and more dishonest than certain prosodies for intermediate levels of gain (p = 0.003, d = 0.65; results were not significant for the lowest gain: doubt vs. certain p = 1; doubt vs. honest p = 1). By contrast, judgments were not significantly different between lying prosodies and honest or certain prosodies at the group level for any level of gains (all p-values > 0.2, and ds < 0.52, all other comparisons were non-significant after Bonferroni correction). Beyond confirming the hypothesis of a common signature, in the certainty task the results also show subtle differences between confidence and honesty for intermediate and high levels of gain: although honest prosodies were judged as more certain than doubtful and lying prosodies for every level of gain (see above), there

were still perceived as less certain than certain prosodies at high ($p < 0.00001$ and $d = 0.84$) and intermediate levels of gain ($p < 0.00001$ and $d = 1.18$). Similarly, although lying prosodies were judged as less certain than honest and certain prosodies for every level of gain (all p-values < 0.00001 and ds > 1.8), there were still judged as more certain than doubtful prosodies for high ($p < 0.00001$ and $d = 0.92$) and intermediate levels of gain ($p = 0.00004$ and $d = 0.79$). For small levels of gain, certain/honest ($p = 0.08$, $d = 0.86$) and lying/doubtful ($p = 0.93$, $d = 0.52$) prosodies were not significantly different. These subtle differences were present only in the certainty task but not in the honesty task, and are most likely due to the fact that there were differences in gain between the archetypes derived from the certainty and honesty tasks (i.e., listeners in study 1 were less precise in the honesty task, leading to kernels with a smaller gain). **B) Conceptual knowledge about epistemic prosody in the group of forty French speakers.** We show the number of participants who provided each of the possible responses for each of the six questions. Chi-squared tests were used to assess whether the frequencies of the responses given to each of the six questions differed from chance level. We show the significant results with a Bonferroni corrected threshold for multiple comparisons, with *: $p < 0.008$; ***: $p < 0.0002$. The only aspects where the distribution differed significantly from chance were the questions about mean pitch for both certainty (X^2=22.5, $p < 0.00001$) and lie (X^2=19.6, $p < 0.00001$), as well as the question about mean loudness for certainty (X^2=10, $p = 0.0015$) but not lie (X^2=0.9, $p = 0.34$). The other contrasts did not differ from chance (certainty: speed $p = 1$, intonation $p = 0.11$, accentuation $p = 0.5$, acceleration $p = 0.75$; honesty: speed $p = 0.2$, intonation $p = 0.2$, accentuation $p = 0.75$, acceleration $p = 0.34$). Source data are provided as a Source Data file.

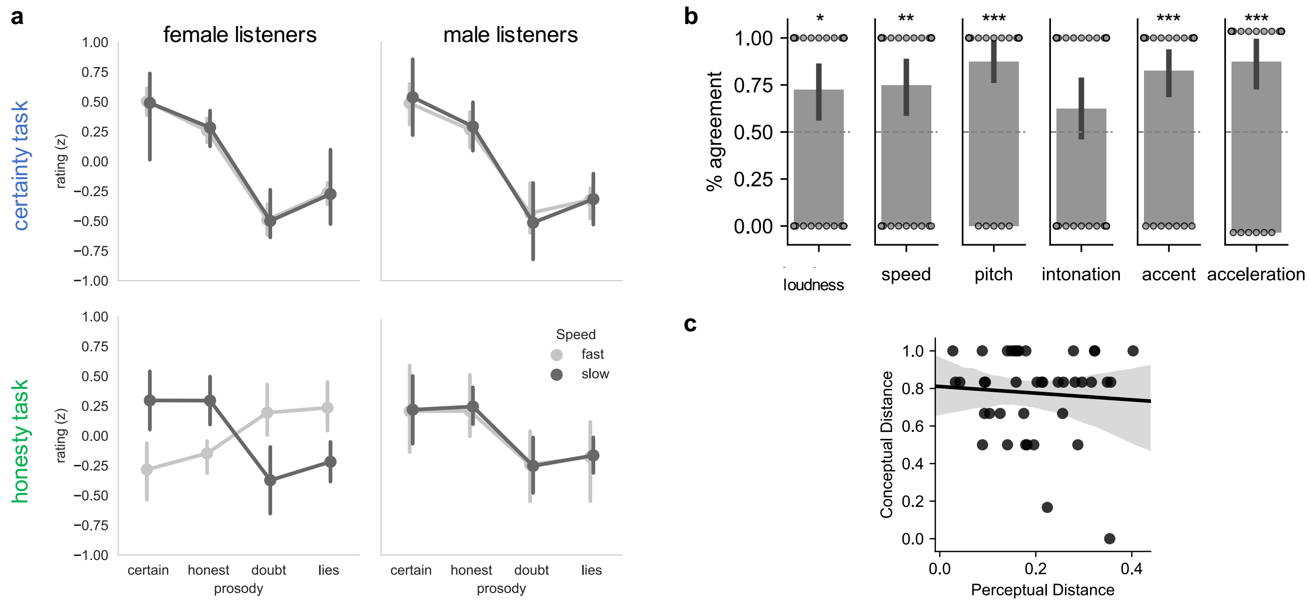

Figure SVII. Relationships between conceptual knowledge and gender (study 2). A) Data show the normalized ratings averaged separately for each prosody, and as a function of listeners' concepts about speed (mean with 95% confidence intervals). In the honesty task, there was a triple interaction between listener's gender, prosody and concepts about speed ($F(3, 48) = 3.16$, $p = 0.03$, $\eta p2 = 0.11$), which reflected the fact that females' reports about the speed of lying archetypes were reflected in their perceptual judgments. Male listeners rated the archetypes in the canonical direction predicted by study 1 regardless of their concepts: they perceived certain/honest prosodies as more certain and more honest (all p-values < 0.001, Bonferroni corrected Tukey HSD). By contrast, female listeners rated the archetypes as predicted in the certainty task regardless of their concepts (i.e., they perceived certain/honest prosodies as more certain, all p-values < 0.001), but most of them performed the honesty task in the opposite direction when they thought that lying prosodies are faster (i.e., they perceived certain prosodies as more dishonest than lying and doubtful prosodies, all p-values < 0.001, and honest prosodies as more dishonest than lying prosodies, p-value < 0.001). Thus, although male listeners perceived epistemic prosodies in a canonic way in both tasks, and regardless of their conceptual knowledge, females' ascriptions of lie in the "poker" context of study 2 depended on their conceptual knowledge about speed. This result, is consistent with the idea that this prosodic signature can be interpreted differently depending on the context, and listeners' identities and concepts. Note that no variations were found concerning the gender of the speaker (all p-values < 0.1, words uttered by the male and the female speaker were judged similarly). Also, note that in the memory experiment (study 4), there were no differences between male and female listeners (i.e., the impact of reliability on accuracy and confidence did not interact with gender, p-values > 0.1). **B)** Percentage of agreement in response to conceptual knowledge questions about epistemic prosody across the two tasks, computed as the percentage of trials in which participants provided the same response to questions about certainty or honesty; * represents two-tailed t-tests against chance level with $p < 0.008$ (Bonferroni corrected threshold for multiple comparisons; $N = 40$ listeners); **: $p < 0.001$; ***: $p < 0.0001$; loudness: $p = 0.003$; speed: $p = 0.0009$; pitch: $p < 0.00001$; intonation: $p = 0.11$; accent: $p < 0.00001$; acceleration: $p < 0.00001$. Bar plots show mean values with 95%

confidence intervals, and dots show individual data points. **C)** There was no correlation between conceptual distance (i.e., the % of agreement for conceptual questions between the two tasks) and perceptual distance (i.e., the average of the absolute difference between ratings given for certain vs. honest or lying vs. doubtful prosodies (Pearson rho(39) = -0.07, p = 0.65). Source data are provided as a Source Data file.

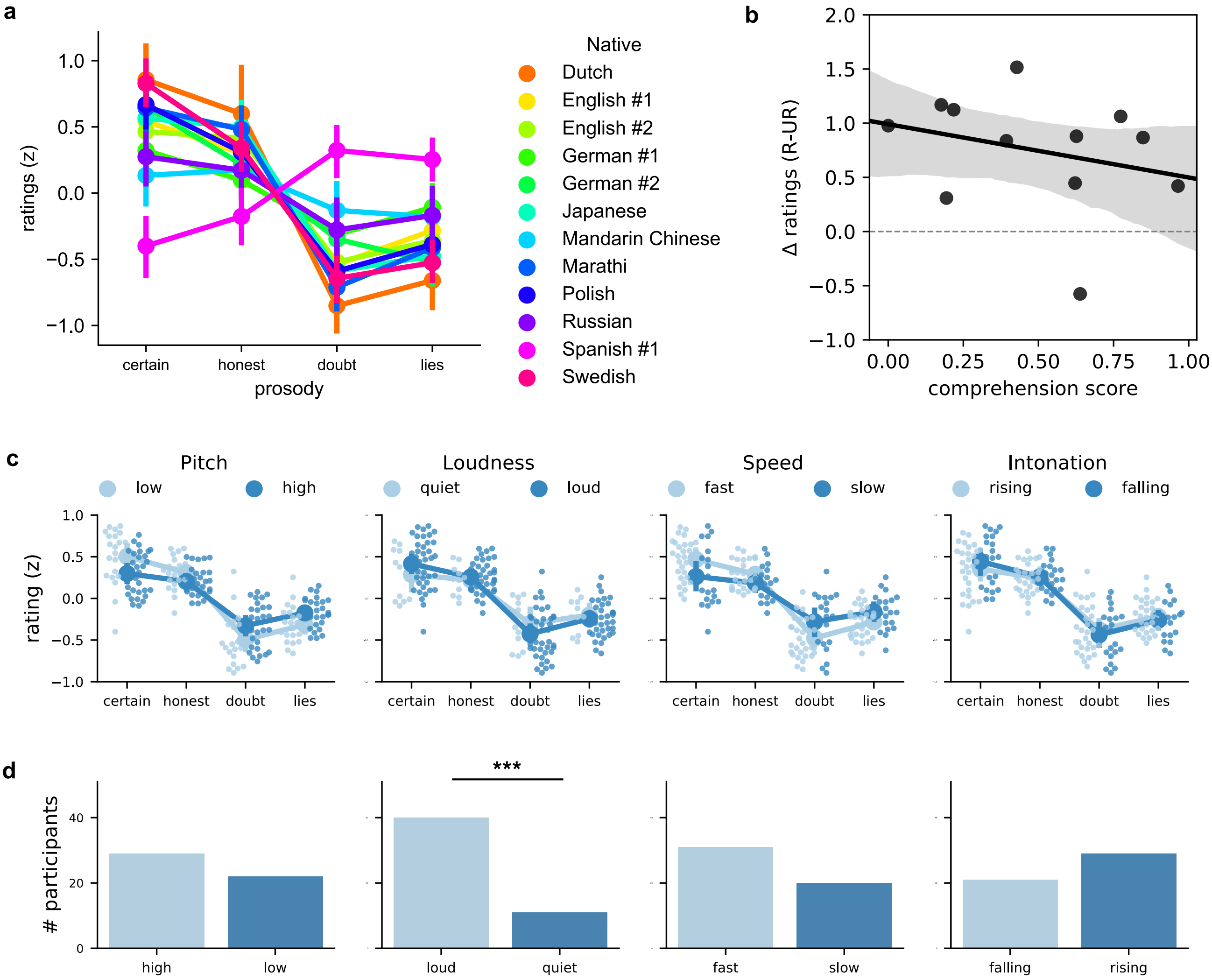

Figure SVIII. Perception of the common signature across language (study 3). A) Social perceptions of certainty depending on listeners' native language for each prosodic archetype in the group of 12 native speakers of German, Dutch, English, Japanese, Mandarin Chinese, Polish, Marathi, Swedish, Spanish and Russian that were familiar with French (and English). Data show the mean ratings for each participant with 95% confidence intervals. The pattern of results was similar to the results obtained with French, English and Spanish speakers (see Figure 4): at the level of the group there was a main effect of prosody on ratings ($F(3,33) = 20$, $p < 0.001$), and listeners perceived certain and honest archetypes as more certain than doubtful and lying archetypes (all post-hoc Bonferroni corrected Tukey HSD comparisons < 0.001). They also perceived certain prosodies to be more certain than honest prosodies ($p = 0.00013$) and lying prosodies to be more certain than doubtful prosodies ($p = 0.04$). At the individual level, all but one female participant (a Spanish speaker) judged the stimuli in the congruent direction. **B)** Ratings as a function of listeners' level of comprehension of spoken French in the multi-language group. Dots show individual data, and the shaded area shows the best fitting regression line with 95% confidence interval. There was no significant correlation between difference scores (ratings for reliable minus unreliable archetypes) and individuals' scores in the objective test of comprehension of spoken French (Pearson rho = -0.27, p = 0.39), confirming that the prosodic signature of reliability that we identified is not language specific. There was no significant relationship between listeners' difference score and their self-reported levels of comprehension of spoken French (rho = -0.49, p > 0.1), and French intonations (rho = 0.07, p > 0.82). There was a negative correlation between their self-reported ability to have a basic conversation and the difference score (rho = -0.59, p = 0.043). Overall, there was no evidence that individual' level of proficiency with the French language positively correlated with their perception of epistemic prosody from the archetypes. **C)** Data represent the mean ratings averaged separately depending on concepts and

prosodic archetypes, with error bars showing the 95% confidence interval. Dots show individual data. Although there was an interaction between prosody-type and concepts about speed ($F(3,135) = 7.1$, $p = 0.0002$) and pitch ($F(3,135) = 4.8$, $p = 0.003$), participants perceived certain and honest archetypes to be more certain than lying and doubtful archetypes regardless of their concepts (all post-hoc Bonferroni corrected Tukey HSD p-values < 0.001). These results are highly consistent with the results observed in the group of French speakers, and confirms that conceptual knowledge does not constrain perception in this task. **D)** Responses to conceptual questions about pitch, loudness speed and intonation in the group of English (N= 19), Spanish (N=20) and multi-language (N=12) speakers (N = 51; 4 participants did not respond to these questions). The bars show the number of participants who provided each of the possible responses for each of the four questions. The only dimension for which participants provided consistent responses was loudness: more participants reported that a certain voice is louder than a doubtful one (40 versus 11 participants, two-sided chisq = 16.5, $p < 0.0001$). For the other acoustic dimensions, participants did not significantly favor one or the other option (pitch: 29 vs. 22, two-sided chisq = 0.96, $p = 0.33$; speed: 31 vs. 20, two-sided chisq = 2.37, $p = 0.12$; intonation 21 vs. 29, two-sided chisq = 1.3, $p = 0.26$; for intonation one participant was excluded because he responded “both”). Source data are provided as a Source Data file.

Study 4.

To further examine how speaker reliability impacted observer’s confidence we also computed an index of metacognitive sensitivity called meta-d’ with the Hmeta-d’ toolbox[2]. This analysis revealed that the decrease in confidence observed in the reliable condition was associated with a marginal decrease in metacognitive sensitivity ($t(39) = 1.94$, $p = 0.059$). However, when correcting for the difference in sensitivity between the two conditions by dividing meta-d’ by d’ (i.e., computing metacognitive efficiency[2]), we found no significant differences between the two conditions ($t(39) = 1. 4$, $p = 0.16$).

References

1. Ponsot, E., Burred, J. J., Belin, P. & Aucouturier, J.-J. Cracking the social code of speech prosody using reverse correlation. *Proc. Natl. Acad. Sci.* 201716090 (2018).
2. Fleming, S. M. HMeta-d: hierarchical Bayesian estimation of metacognitive efficiency from confidence ratings. *Neurosci. Conscious.* **2017**, (2017).
