# Supplementary material for: Listeners’ perceptions of the certainty and honesty of a speaker are associated with a common prosodic signature
Source: Nat Commun. 2021 Feb 8;12:861. doi: 10.1038/s41467-020-20649-4 (PMC7870677; doi:10.1038/s41467-020-20649-4)
Supplement: Supplementary file 1 — Peer Review File [file 41467_2020_20649_MOESM1_ESM.pdf]

Reviewers' comments:

Reviewer #1 (Remarks to the Author):

Goupil and colleagues present a novel study of the role of prosody in implicit inference about speakers' reliability or confidence. Data driven techniques grounded in a reverse correlation approach are used to reveal features of intonation, intensity and speed of speech that affect perceived confidence. Given that most research in human metacognition, particularly in social interactions, has focused on explicit communication, the demonstration of implicit features of communication such as prosody that may play a inter-personal metacognitive function is very interesting indeed. I expect this paper will catalyse a range of future studies examining the impact of such features in collaboration or cooperation.

In general I found the paper to be a pleasure to read and the analyses clear and thorough. The main focus of my comments is on the interpretation of the metacognitive differences between the honesty and confidence conditions.

Major:

- In Study 1, the claim is made that metacognition was significantly better when judging confidence than when judging honesty. If true, this would be very interesting, but I'm less convinced that this is not an artefact of differences in first-order performance. The honesty judgments are significantly worse than the confidence judgments as shown by the shallower overall psychometric function slope in Figure 2D. In addition, the psychometric functions are fit to points clustered around the midpoint in the honesty condition which may make this analysis less sensitive to differences in confidence – as better performance will lead to a wider range of data points, and more reliable estimates of the difference in slope. I appreciate that a more standard performance-controlled measure of metacognition is harder to evaluate here given that the analysis rests on the relation between a continuous variable (AUC) and the participants' decisions. But I wonder whether first-order performance can still be controlled for at the analysis stage – e.g. using a multiple regression or ANCOVA approach when comparing the difference in slope between conditions. At the very least, limitations on the extent to which differences in metacognition can be inferred in the presence of performance differences needs to be acknowledged and discussed.

- Following on from the previous point, I found the claim on p. 12 that deception detection may be better at implicit than explicit levels odd, given that the *implicit* detection was also relatively poor here for the honesty decisions.

- Again on the difference between drivers of first-order and metacognitive decisions – would it be possible to sort the reverse correlation kernels obtained in Figure 1 by observer confidence (self-confidence) level to identify features of the stimuli that are driving each of these effects? It might be that high confidence kernels look like boosted versions of the average kernels. But it seems possible, especially with the observation of metacognitive differences between conditions, that the features that

drive these judgment types may differ.
- Might memory for the words differ for reasons that are not to do with inferred reliability? For instance, if non-vocal sounds were similarly manipulated, might they also be easier/more difficult to remember due to differences in the lower-level auditory features?

- The details of the SDT model used to extract a value of internal noise on p. 11 was not clear. Could more detail/equations be provided in Methods?

Minor:

- I was sometimes confused about the term “confidence”, and whether this referred to either inferred speaker confidence or confidence in the participant’s judgment about this. The authors are presumably aware of this potential confusion too choosing to use “self-confidence” for the latter. But I found this less helpful – the more usual usage of the term confidence in the literature is for observer confidence, so a clarification here is not needed. Instead, how about using “perceived confidence” or similar for the other-directed judgment?

- I found the layout of Figure 2 particularly confusing. Which panels belong with (A) and which with (B)? It would be helpful if some rearranging can be done to make this clear; currently, even with a careful reading of the figure legend, it’s ambiguous.

- Should the y-axis of the lower figure in panel (B) be “% agreement (honesty)” rather than lie?

Reviewer #2 (Remarks to the Author):

Across three studies, the authors investigate whether and to what extent variations in acoustic properties associated with pitch, loudness, and duration (speech rate), influence perceptions of speaker confidence and honesty. Broadly summarized, the data reveal substantial overlap in the acoustic properties perceivers associate with confidence and honesty within each of the three vocal qualities, and across speakers of different languages (French, English, Spanish). Interestingly, although identification of the acoustic markers of confidence and honesty appear to occur relatively automatically (i.e., without careful thought), explicit instructions to identify how variations in vocal qualities are related to confidence and honesty produced largely conflicting results (little consensus among participants).

The novel methodology and myriad statistical analyses were interesting to read and reflect a degree of creativity deserving of recognition. That said, my review predominantly focuses on conceptual aspects as well as the question of whether this research is truly novel, and to what extent it advances current empirical research related to vocal properties and person perception, broadly construed. Because I have attached a copy of the manuscript to this review that includes comments I made while reading, my

comments here will necessarily be abbreviated.

The introduction overlooks a great deal of directly relevant, current research that would not only be very informative to the reader, but in a number of cases answers the questions posed by the authors themselves.

For example, the discussion of prosodic features of speech related to doubt and uncertainty on page 3 could benefit by including research from the person perception and attitudes literatures that describe how variations in pitch, speech rate, intonation, and loudness affect perceptions of doubt/uncertainty, amongst other things.

Secondly (page 3), the question of “whether rising intonation and slower speech rate manifest in the same way for both honesty and doubt” can be addressed via research by Hicks (2011), Nahari (2019), Perez-Rosas (2015), Shaw (2017), and Vrij (2018), to name only a few.

Third (page 4), “whether epistemic prosody can be perceived cross-linguistically” is not unknown as the authors suggest. The authors would do well to consider research by Johnson, Ernde, Scherer, & Klinnert (1986), Mandal (2008), and Pell, Monetta, Paulmann, & Kotz (2009), amongst others.

Fourth (page 4), although certainly it is true that “our understanding of how we communicate honesty and confidence in speech is likely to be both inaccurate and incomplete”, the fact that directly relevant, current research addressing this point (Guyer et al. 2018; Van Zant & Berger, 2019) was not even mentioned suggests that the authors could benefit from having conducted a more careful and thorough literature review.

Fifth (page 7), the pattern of effects observed for perceptions of honesty and confidence across intonation, pitch, loudness, and volume have been found in numerous prior studies. Thus, these findings add no new information regarding how variations in these vocal properties affect perceptions of confidence, honesty, and attitudes that has not already been shown in many studies within both the person perception and attitudes/persuasion literatures. (e.g, Brennan & Williams, 1995; Brown, Giles, & Thakerar, 1985; Guyer, Fabrigar, & Vaughan-Johnston, 2018; Jiang & Pell, 2014; Kimble & Seidel, 1991; London, 1973; Scherer, London, & Wolf, 1973; Smith & Clark, 1993; Van Zant & Berger, 2019, etc.).

Related to studies 1 and 2, it seems like the core take-home message is that the acoustic properties of voice that reflect confidence and honesty (and unconfidence/dishonesty) are independent of language. In other words, regardless of everything that differs between speakers of different languages, we all use highly similar acoustic markers to evaluate the extent to which a speaker is confident and honest. I would encourage you to consider these questions at a more fundamental level. For example, what is it about pitch or loudness that leads people to infer confidence? Do variations in these acoustic properties of voice reflect physical or social power, dominance, importance, weight, substance, larger size, strength, etc? Are these associations evolutionarily driven, and do these patterns also emerge among various species of primates?

Related to your conclusion on page 20 “Overall, words pronounced with unreliable prosodies were memorized better than reliable ones, and participants responded faster in this condition.”
It would seem to be evolutionarily adaptive to be able to identify acoustic markers that signal deception, given the diagnosticity of these cues as they relate to a recipient’s attitudinal and behavioral responses to a source attempting to deceive. So how do you explain these findings in light of a large body of research showing that both laypersons and professionals in careers where lie detection is of paramount importance (e.g, detectives, FBI agents, border control, etc.) perform no better than chance when attempting to detect lies?

I like the question you raised on page 23, linked to study 2: “This is compatible with an inferential model of mental attitude attributions, in which indices can be interpreted differently depending on the context in which they are perceived…” I wonder to what extent contextual factors such as the potential consequences of an error in judging the confidence/honesty of a speaker might moderate the threshold of the acoustic cues used to infer judgements of confidence/honesty. In other words, in contexts where the severity of the consequences for inaccurate evaluations of a source’s confidence/honesty might be particularly high, we might also anticipate that the threshold used by message recipient’s to evaluate perceptions of confidence/honesty may change by becoming more stringent.

I was somewhat surprised/confused by your comment on page 25: “This suggests that our results should apply to real spoken words embedded in discourse, but further studies should be designed to confirm this.” The reason for my surprise/confusion is, as previously noted, the fact that a growing body of research dating back more than 40 years has already extensively examined this, and other closely related questions.

Additionally, you asserted on page 25 that: “In addition, these findings shed light on the perceptual and metacognitive processes underlying such epistemic vigilance, and reveal that it can rely on implicit mechanisms that enable the quick and efficient detection of unreliability…” In my opinion, I’m not sure that any of these studies have included measures that would allow the researchers to systematically evaluate the underlying psychological mechanisms (mediators?) that might contribute to the perceptual and metacognitive processes driving these effects. In my view, this claim is not well supported by the methodology or analyses.

Overall, the paper seems underdeveloped, presents too simplistic a view, and overlooks a considerable body of relevant, current empirical evidence that would contribute in important ways to the reader’s understanding of this phenomena. Simply revising the manuscript in an attempt to address coverage of missing literature would only, in my opinion, further highlight the largely redundant nature of the authors findings. To be fair, the authors do present data that advances existing literature on voice and person perception, though only in a very incremental fashion. In my opinion, the critical flaw in this manuscript is that the key questions that form the basis of these studies have already largely been answered by a considerable body of research in the person perception and attitudes/persuasion literatures over the last 40 years. Thus, I regrettably cannot recommend this manuscript for publication

as I believe it lacks the novelty and significant advances in knowledge that warrant publication in Nature Communications.

Reviewer #3 (Remarks to the Author):

In this ms., the Authors give participants pairs of pseudowords that have been manipulated to vary along a number of dimensions (pitch, volume, etc.). Participants are asked to tell which of the two pseudowords was spoken with the most confidence or the most honesty. The Authors can then extract the features of the pseudowords that best explain participants' decisions. On the whole, I believe this is an interesting ms. I know next to nothing about prosody, so I won't dwell on that, focusing instead on the theoretical framework.

I believe the Authors should more clearly indicate that the cases of confidence and of honesty are completely different. In the case of confidence, speakers have an incentive to communicate their degree of confidence. For instance, speakers might want to communicate that they are not so sure, in order to be blamed less if the information they communicate proves unreliable. As a result, every language has many confidence signals (for the terminology, see Scott-Phillips, 2008).

By contrast, when it comes to honesty, speakers have no incentive to communicate that they are being dishonest (except maybe in very rare occasions). As a result, if something in speech (or gesture) allows listeners to detect that a speaker is dishonest, it is a cue, and not a signal (again, see Scott-Phillips, 2008). Evolutionary theory suggests that there should be no reliable cues to dishonesty, as these cues are obviously costly for senders, and there is no good reason for them to exist. Indeed, as mentioned above, the literature on lie detection suggests that there are no such cues to dishonesty (based on behavior, gestures), and that, as a result, people are consistenly at chance at detecting lies from truth using only such cues (by contrast with diagnostic evidence such as knowing the speaker's incentives to lie, or the content of the speech).

As a result, the Authors appear to conflate signals of confidence and cues to honesty, even though these two things are quite different.

Given this, I would suggest that the best way to interpret the Authors' results is that participants are good at detecting confidence signals (such as volume), and that they then use exactly the same signals when they have to answer to the honesty question. I believe quite a lot of the Authors' data suggest this interpretation is likely correct. As the Authors state "These analyses demonstrate that, despite non-negligible inter-individual differences regarding the shape of these kernels, individual participants each relied on very similar mental prosodic representations to evaluate confidence and honesty." Moreover, for every feature associated both with confidence and with honesty, the association is stronger with confidence. There are no marker of honesty per se. Confidence is higher for confidence than honesty judgments.

One way of testing the hypothesis that people are only processing confidence signals would be to pit the signals the Authors are studying against other information relevant to the speakers' confidence or honesty. My guess would be that the signals the Authors observed would still play a role when the participants evaluate confidence, but that they would essentially disappear if confronted with reliable information that someone is likely to be honest or dishonest (e.g. their incentives).
I believe that the Authors should seriously consider this hypothesis (i.e. that participants are relying on confidence signals to answer the honesty question), and either explicitly endorse it, or then provide some arguments against it.

Minor points:

"Research in linguistics has shown that some languages possess ostensive markers of epistemicity5,6, that speakers can use to deliberately communicate their level of confidence. These markers often consist in dedicated expressions such as "I don't know"7,8, but speakers may also rely on socio-pragmatic means to convey confidence (e.g., doctors may prominently point out that they are doctors)6,9. These markers are not present in every language and culture however5,10"

The last sentence is false: every language has means of encoding both modality and evidentiality. The Authors appear to be confusing the fact that modals or evidentials aren't grammatically mandatory in every language with the fact that modality or evidentiality can't be expressed. For example, English doesn't have evidentials, but it's possible to say in English "I saw" or "Peter told me" (or "I don't know" instead of a modal, in the Authors' own example).

"even young children appear to filter information from unreliable informants"

Given the wealth of evidence on this topic, the 'appear' should be removed.

"Similarly to doubt, speech rate appears to decrease22,23 and pitch to increase24 during lies."

On the whole the literature on lie detection suggests that all such cues are extremely weak and unreliable.

"Thus, the kernels were not affected by whether or not participants performed the other task beforehand (e.g., kernels appeared similarly in the confidence task whether or not participants had performed the honesty task before), which rules out an interpretation in terms of low-level strategies."

I don't understand what these low-level strategies are (they don't seem to have been introduced before)

The Authors should justify their small Ns (maybe by pointing out the number of trials per participant, certainly with some power analysis). Given the high level of inter-individual variance, maybe a higher N

would be desirable.

It would be nice to have effect sizes for all the results, a number of them being borderline significant.

Scott-Phillips, T. C. (2008). Defining biological communication. Journal of Evolutionary Biology, 21(2), 387–395.

Reviewer #4 (Remarks to the Author):

With three perceptual-acoustic studies, this study revealed the prosodic encoding and decoding of the reliability information in speech. The reverse-correlation analysis revealed a systematic change in the pitch, length and intensity cues that is commonly involved in making confidence and dishonest judgments. Such acoustic cues were independent of the listener's explicit knowledge of the link of the reliability attributes and the cues and of the listener's native tongue. Last but not the least, the speaker reliability affected the listener's memory performance. Moreover, the perceptual and memory outcome that were associated with the listener's meta-cognitive ability was also revealed across studies. These findings reveal a unique mechanism that supports the rapid detection and response towards speaker reliability cues in linguistic communication.

Overall this is a well-conducted study with rigorous statistic evidence that provides unique knowledge regarding the human perception of speaker reliability. The perceptual relevance of prosodic cues of one's mental states and how it is linked with listener's metacognitive ability is a highly relevant and widely neglected in the field of human nonverbal communication, given that no previous reports have considered (un)confidence and (dis)honesty simultaneously in a single research. Importantly, the study employed a data-driven approach (relative to a conventionally used elicitation paradigm to enhance the spontaneity of the speech) that explored (in study 1) and manipulated (in study 2) the acoustic features to examine the common prosodic cues that were explicitly (in study 1 and 2) vs. implicitly (in study 3) used by the listener to judge or recall speaker information.

In addition to the above merits, I have some minor comments that I hope to help strengthen the arguments.

1. The use of reverse correlation method is rigorous. I'm just not quite convinced how the perceptual judgment on “randomly” varied speech prosody is justified to identify the typical, representative cues that indicate confidence, and how the manipulation of the cues identified this way could generalize to the overall pattern of confidence. Based on recent study, the neutral prosody (even without any explicit manipulation of cues) can be perceptually judged as confident. The consideration of the current approach in this context would be very helpful.

2. On p6, the acoustic extraction and analysis was based on 12 temporal points in the sample. However, in the method, the author mentioned the stimuli was created by random manipulation of acoustic values on FOUR consecutive windows. I was a bit confused but how was exactly the acoustic cue manipulated as the unfolding of the stimuli. Please clarify a bit here.

3. Fig. 1, a bit clarification regarding what the “filter amp” index would be helpful.

4. The instruction of the prosody task seemed asymmetrical, with the instruction of confidence task focusing on the positive end (“confident”) while that of honesty task focusing on the negative end (“dishonest”). I’m curious how focusing on different ends of the scale of mental attributes could potentially drive the listener’s perception. Some discussion would be needed.

5. On the finding of common code in study 1. Could the similar reverse correlation kernels obtained along acoustic cues for confidence and honesty judgments be due to the same participants who was asked to judge on both attributions in a sequential order? The cues that were reliably significant in predicting the second social attribute in a sequence maybe influenced by that in the first. How could this possibility be ruled out from the findings?

6. While the study used shorter stimuli rather than the longer one (such as pseudo-sentence), how could the finding in the pseudoword be generalized to longer stimuli in terms of the confidence/honesty encoding? The discussion can be meaningful given that the natural, longer speech stimuli may bring more variations in acoustic cues that contain dynamic information (e.g. intonation, accentuation, etc.).

7. On the larger individual difference in perceiving honesty than confidence in speech. The honesty judgment may depend on more contextual factors (did speaker gender or any particular token affect the honesty perception?) and may be supported by a larger variation of acoustic cues. These discussions may need justifications by more analysis.

8. despite that study 2 included language groups other than French as out-group listeners, no language difference was found between French and these out-group listeners. Could the use of pseudo-speech reduce the perceived language and therefore prevent from any in-group advantage effect?

9. On p22 despite that “the listeners use acoustic consequences of cognitive efforts as an index of reliability” is a very interesting point, study 2 still revealed differential interindividual differences between confidence and honest task. How could such account fit into these findings that demonstrate the distinction between the two tasks?

10. On p23 “individuals’ ability to evaluate the inferences about social attitudes from prosody relates to empathic trait” This could be interesting however there is only trend level difference in suggesting such relation between empathy and metacognitive ability. Some discussion in the context of recent findings demonstrating the link between vocal confidence and listener interactive traits would strengthen these arguments more.

11. How could the female advantage in deriving contextual meaning from the unreliable cue be linked in previous literature, given that some recent evidence showing similar advantage of using prosodic cues to infer mental states or resolving mixed cues in female and the increased sensitivity towards social information in these populations (e.g. Jiang & Pell, 2016)?

12. The use of “confidence” for the perceptual task and “self-confidence” (and sometimes also “confidence”) for the metacognitive task is a bit confusing. Please correct whenever possible to avoid ambiguity.

Reviewer #1 (Remarks to the Author):

Goupil and colleagues present a novel study of the role of prosody in implicit inference about speakers' reliability or confidence. Data driven techniques grounded in a reverse correlation approach are used to reveal features of intonation, intensity and speed of speech that affect perceived confidence. Given that most research in human metacognition, particularly in social interactions, has focused on explicit communication, the demonstration of implicit features of communication such as prosody that may play a inter-personal metacognitive function is very interesting indeed. I expect this paper will catalyse a range of future studies examining the impact of such features in collaboration or cooperation.

In general I found the paper to be a pleasure to read and the analyses clear and thorough. The main focus of my comments is on the interpretation of the metacognitive differences between the honesty and confidence conditions.

We thank the reviewer for these positive comments that allowed us to clarify our contribution.

Major:
- In Study 1, the claim is made that metacognition was significantly better when judging confidence than when judging honesty. If true, this would be very interesting, but I'm less convinced that this is not an artefact of differences in first-order performance. The honesty judgments are significantly worse than the confidence judgments as shown by the shallower overall psychometric function slope in Figure 2D. In addition, the psychometric functions are fit to points clustered around the midpoint in the honesty condition which may make this analysis less sensitive to differences in confidence – as better performance will lead to a wider range of data points, and more reliable estimates of the difference in slope. I appreciate that a more standard performance-controlled measure of metacognition is harder to evaluate here given that the analysis rests on the relation between a continuous variable (AUC) and the participants' decisions. But I wonder whether first-order performance can still be controlled for at the analysis stage – e.g. using a multiple regression or ANCOVA approach when comparing the difference in slope between conditions. At the very least, limitations on the extent to which differences in metacognition can be inferred in the presence of performance differences needs to be acknowledged and discussed.

This is an important point and we thank the reviewer for raising it. It is indeed critical to control for first-order differences in sensitivity to assess the specific impact of task on metacognitive sensitivity. As pointed out by the reviewer, this is an unusual situation to look at these questions, because it involves continuous multidimensional evidence, and because the ground truth is not known, contrary to most psychophysical tasks typically employed to measure metacognitive sensitivity (e.g., deGardelle et Mamassian, 2015; Fleming and Lau, 2014…).

Initially, it seemed safe to us to interpret differences in metacognitive sensitivity directly, given that our analysis at the level of dynamic kernels showed no significant differences between the tasks for the three acoustic dimensions, as we detail on p. 9 to 14. This analysis suggested that there were no significant differences at the level of perception in between the

two tasks, although we also observed through the analysis of internal consistency and internal noise that listeners were less consistent in their judgments for honesty.

Following the reviewer's observation that the slope appears steeper for the certainty task than for the honesty task, we tested differences at the level of individual psychometric function slopes, which may indeed be more powerful to capture differences in sensitivity, because it combines all three acoustic features. A statistical test of the difference in slopes between the two tasks (combining high and low confidence responses) indeed revealed a significant difference. From this measure of sensitivity, we could then compute a measure of metacognitive efficiency, by dividing the measure of metacognitive sensitivity (the difference in slopes between high and low confidence trials) by the measure of sensitivity (the global slope). This reveals that, indeed, the differences observed in metacognitive sensitivity are likely due to underlying differences in sensitivity (see p.18 l.417 to p.19 l.440 for a full report, and the updated Figure 2 below). We thank the reviewer for this suggestion.

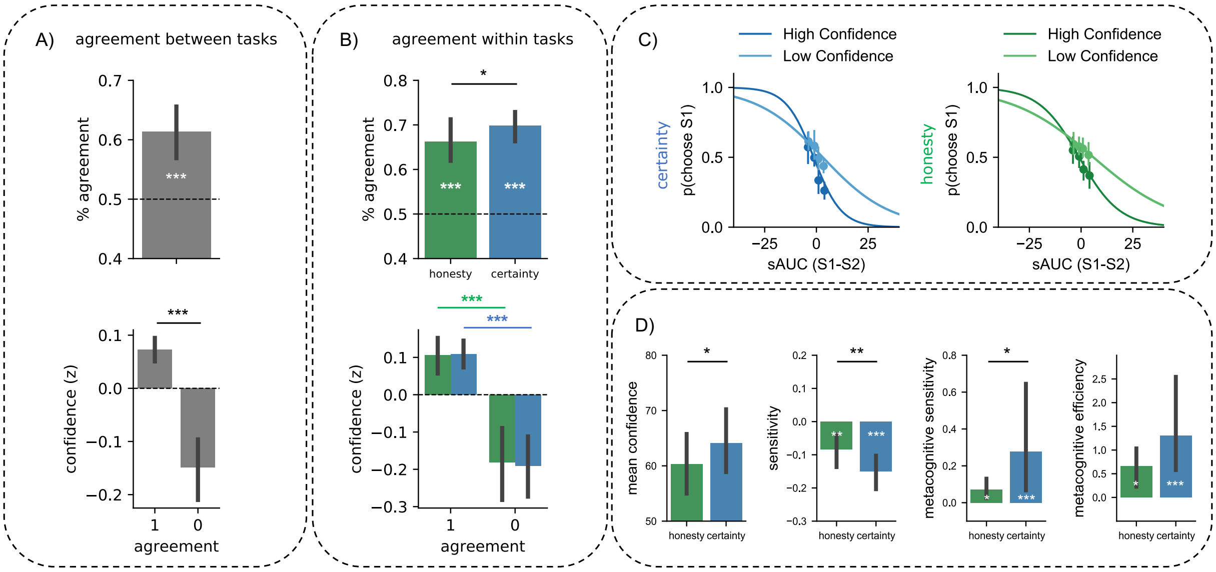

Importantly, previously reported differences between the two tasks regarding stability and precision still hold: decisions are less stable, less precise and participants are less confident in the honesty task than in the certainty task. This is likely because a judgement about the probable honesty of a speaker can hardly be reduced to a perceptual decision, as listeners must also decipher the speaker's intention in showing a particular prosodic display. In order to force listeners to make these judgements at a strictly perceptual level, such contextual/intentional information was not given to the participants in the first study, which we think explains the less precise and less confident responses in the case of honesty. We have clarified this aspect in the manuscript on p.6/7, p.20/21 and p.33/34 (also see responses to reviewers #2 and #3 below).

- Following on from the previous point, I found the claim on p. 12 that deception detection may be better at implicit than explicit levels odd, given that the *implicit* detection was also relatively poor here for the honesty decisions.

We lacked clarity here. What we meant – in accordance with a model proposed by tenBrinke et al., 2016 - is that listeners appear to be more sensitive to cues of deceit than what their

explicit reports suggest. We base this claim on the results we obtained in the fourth study (implicit memory bias), on the dissociation we observed between percepts and concepts in the second study, and on past research showing poor explicit deception detection abilities in laboratory settings.

While sensitivity was lower in the honesty task as compared to the certainty task in the first study, it was still highly significantly above chance when cumulating the three sources of sensory evidence ($M = -0.084 +/- 0.1$ std, $Z = 18$, $p = 0.01$, $d = 0.8$), as we now detail in the manuscript. Although not directly comparable, this effect size is higher than what is typically reported for reports of dishonesty in detection tasks, as we now also clarify on p.34 (l.817): *"In the first study where contextual and decisional factors were neutralized in a forced-choice procedure, listeners' sensitivity to prosodic displays was highly significantly above chance in the honesty task, with a large effect size. Although not directly comparable, this contrasts with the medium to low effect size found in a meta-analysis compiling average levels of accuracy in explicit deception detection tasks (i.e., that reflect both observers' sensitivity and their biases, Bond and dePaulo 2006)."*

- Again on the difference between drivers of first-order and metacognitive decisions – would it be possible to sort the reverse correlation kernels obtained in Figure 1 by observer confidence (self-confidence) level to identify features of the stimuli that are driving each of these effects? It might be that high confidence kernels look like boosted versions of the average kernels. But it seems possible, especially with the observation of metacognitive differences between conditions, that the features that drive these judgment types may differ.

Thank you for this suggestion. We in fact had a look at this particular issue, but decided not to include it in the initial manuscript because it would considerably increase its complexity and length. Yet, we agree that it may interest some readers to have more information about how confidence judgements vary with the different features in this multi-dimensional reverse correlation task. We now include a Figure (SV) detailing these results in the supplementary materials.

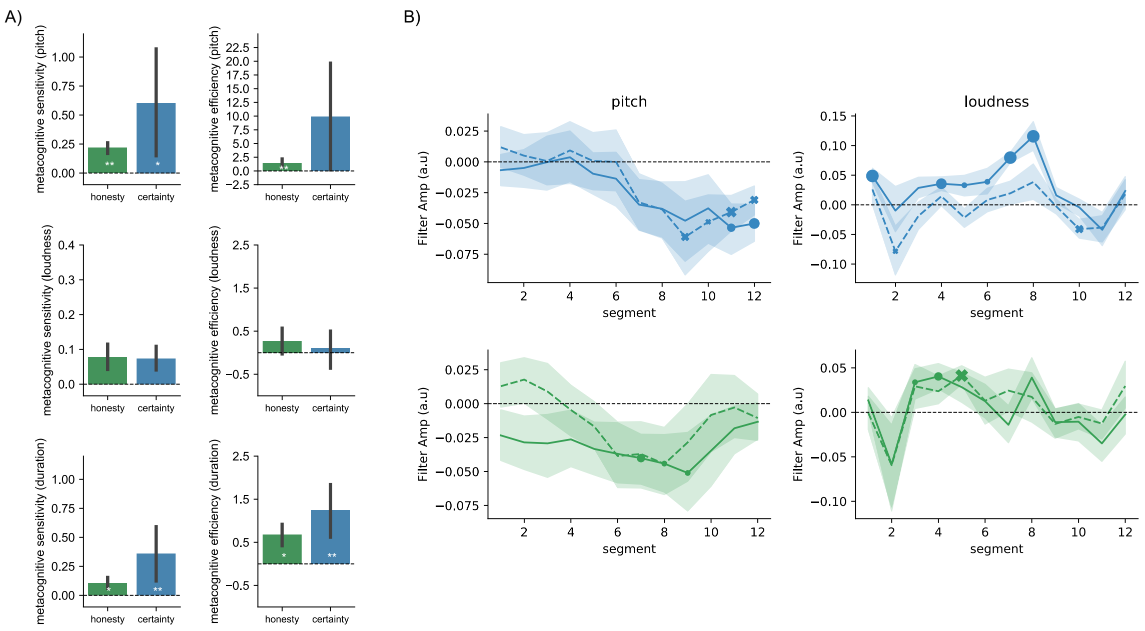

This Figure shows metacognitive sensitivity and efficiency computed separately for each acoustic dimension (A), as well as the reverse correlation kernels computed separately for high and low confidence trials and each acoustic dimension (B). The main conclusion (no differences in metacognitive efficiency between tasks when taking sensitivity into account) holds when zooming in on particular acoustic dimensions. This fine-grained analysis reveals subtle differences between tasks depending on acoustic dimensions, but linear regressions testing the impact of acoustic dimensions on sensitivity (X2 = 4.505, p > 0.10), metacognitive sensitivity (X2 = 2.9734, p > 0.2) and efficiency (X2 = 2.2607, p > 0.3) did not reveal significant differences, so these subtle variations depending on the acoustic dimension – although potentially interesting, are difficult to interpret here. Given this, we kept our original analysis, encompassing all three dimensions, in the manuscript, but now provide the analysis per acoustic dimension shown above in the supplementary materials.

- Might memory for the words differ for reasons that are not to do with inferred reliability? For instance, if non-vocal sounds were similarly manipulated, might they also be easier/more difficult to remember due to differences in the lower-level auditory features?

This point, which also parallels comments made by reviewer #2 and #3, is important. It is true that we cannot assume that listeners attributed honesty or certainty to the speaker in such task, which does not require any elaborated social attribution of that kind. The results show that the prosodic signature can be processed in an automatic fashion – as also discussed with reviewer #4, and as we would reasonably expect if it reflects a specific cognitive regime characterized by more effort, leading to less fluent and powerful speech production that would capture attention because they are rare (i.e.,"pop-out" attentional effect). The reviewer's question whether non-vocal sounds would also yield similar effects is indeed important to address, and could reveal more generic effects. We now clarify this as follows on p.31 (l.723): *"It is unlikely that the effect observed here entails attributions of honesty or certainty to the speaker per se, especially since social attitudes were totally irrelevant in this task. Rather, the results suggest that the prosodic signature of unreliability automatically impacts working*

memory, consistent with the idea that it reflects a rare regime of speech production that "pops-out" as compared to typical (effortless, or neutral) prosody (Jiang & Pell, 2017), thereby attracting attention in an exogenous fashion. Further research testing whether this effect extends to non-speech sounds could establish the level of processing at which this acoustic signature are prioritized, and whether it occupies a dedicated niche in the acoustic landscape, and triggers increased alertness regardless of the type of sound that carries it, as recently shown for roughness(Arnal, Flinker, Kleinschmidt, Giraud, & Poeppel, 2015)".

- The details of the SDT model used to extract a value of internal noise on p. 11 was not clear. Could more detail/equations be provided in Methods?

Absolutely, we now provide a detailed explanation of the model, along with the appropriate references (p.41/42, l.996 to 1022).

Minor:

- I was sometimes confused about the term "confidence", and whether this referred to either inferred speaker confidence or confidence in the participant's judgment about this. The authors are presumably aware of this potential confusion too choosing to use "self-confidence" for the latter. But I found this less helpful – the more usual usage of the term confidence in the literature is for observer confidence, so a clarification here is not needed. Instead, how about using "perceived confidence" or similar for the other-directed judgment?

This is a good point, also made by reviewers #2 and #4. We now refer to these social attitudes as *certainty* (vs doubtful) and *honesty* (vs. lying or dishonest), and we only use the word *confidence* to refer to participants' judgements about their social attributions.

- I found the layout of Figure 2 particularly confusing. Which panels belong with (A) and which with (B)? It would be helpful if some rearranging can be done to make this clear; currently, even with a careful reading of the figure legend, it's ambiguous.
- Should the y-axis of the lower figure in panel (B) be "% agreement (honesty)" rather than lie?

Absolutely, we have now totally reworked this Figure (see above).

Reviewer #2 (Remarks to the Author):

Across three studies, the authors investigate whether and to what extent variations in acoustic properties associated with pitch, loudness, and duration (speech rate), influence perceptions of speaker confidence and honesty. Broadly summarized, the data reveal substantial overlap in the acoustic properties perceivers associate with confidence and honesty within each of the three vocal qualities, and across speakers of different languages (French, English, Spanish). Interestingly, although identification of the acoustic markers of confidence and honesty appear to occur relatively automatically (i.e., without careful thought), explicit instructions to identify how variations in vocal qualities are related to confidence and honesty produced largely conflicting results (little consensus among participants).

The novel methodology and myriad statistical analyses were interesting to read and reflect a degree of creativity deserving of recognition. That said, my review predominantly focuses on conceptual aspects as well as the question of whether this research is truly novel, and to what extent it advances current empirical research related to vocal properties and person perception, broadly construed. Because I have attached a copy of the manuscript to this review that includes comments I made while reading, my comments here will necessarily be abbreviated.

We thank the reviewer for these detailed comments, which give us the opportunity to clarify why we think our results are novel with respect to the existing literature.

The introduction overlooks a great deal of directly relevant, current research that would not only be very informative to the reader, but in a number of cases answers the questions posed by the authors themselves. For example, the discussion of prosodic features of speech related to doubt and uncertainty on page 3 could benefit by including research from the person perception and attitudes literatures that describe how variations in pitch, speech rate, intonation, and loudness affect perceptions of doubt/uncertainty, amongst other things.

We would like to point out that, with the exception of two recent papers published since our study was initiated (Guyer et al. 2018; Van Zant & Berger, 2019; to which we come back below), all of the references provided by the reviewer were in fact already indirectly covered in our initial manuscript through secondary references to e.g. Gussenhoven, 2002; Jiang and Pell, 2017; Roseano et al., 2016; Pon-Barry, 2008; Krahmer, E. & Swerts, 2005; Dijkstra et al., 2006; Fish et al., 2017; Villar et al., 2013, tenBrinke et al., 2016, etc.). Nonetheless, to address this comment, we have now considerably expanded our description of past studies. We describe below how we detail the literature concerning honesty in response to other related comments made by the reviewer. Concerning certainty, we expanded our description of past studies in two main directions.

First, we now clarify in the introduction on p. 3/4 that most studies examining social attributions of certainty have conventionally relied on elicitation paradigms, and describe the typical results obtained with these approaches. In passing, we note several published discrepancies in patterns of pitch and speech rate (e.g., Van Zant & Berger, 2019, found that persuasion was associated with higher pitch, and Jiang and Pell 2017, that doubt was associated with higher pitch), and discuss why these contradictions would occur (l.66 to 98): *"Research in this field typically involves elicitation procedures comprising two phases(Brennan & Williams, 1995; Dijkstra, Krahmer, & Swerts, 2006; Jiang & Pell, 2017; Roseano, González, Borràs-Comes, & Prieto, 2016; Van Zant & Berger, 2019). First, encoders (trained actors(Jiang & Pell, 2016b, 2017), or speakers in a semi-naturalistic setting(Brennan & Williams, 1995; Van Zant & Berger, 2019)) are recorded while expressing utterances with various levels of certainty. In a second phase, acoustic analysis of these recordings is performed, and listeners are asked to recover the degree of certainty expressed by the speakers. Acoustic analyses of these recordings typically reveal that speakers' uncertainty is associated with decreased volume and rising intonation(Brennan & Williams, 1995; Dijkstra et al., 2006; Goupil & Aucouturier, n.d.; Jiang & Pell, 2017; Roseano et al., 2016), and to a lesser extent higher(Jiang & Pell, 2017) (yet also sometimes lower(Van Zant & Berger, 2019)) mean pitch as well as slower(Brennan & Williams, 1995; Jiang & Pell, 2017; Kimble & Seidel, 1991; Van Zant & Berger, 2019) (yet also sometimes faster(Goupil & Aucouturier, n.d.)) speech rate.*

While these studies show that listeners are able to infer speakers' uncertainty from the sound of their voice(Brennan & Williams, 1995; Dijkstra et al., 2006; Jiang & Pell, 2016a, 2017; Kimble & Seidel, 1991; Van Zant & Berger, 2019), the precise perceptual representations used by listeners to perform these judgments remain unclear. First, because these prosodic signatures are typically examined in procedures where speakers deliberately produce them, it is unknown whether, at a fundamental level, they are inherently communicative (i.e., natural or conventional signals)(Dezecache, Mercier, & Scott-Phillips, 2013; Wharton, 2009) as opposed to constituting natural signs(Wharton, 2009), e.g., of cognitive effort(Goupil & Aucouturier, n.d.; Gussenhoven, 2002; Kimble & Seidel, 1991). Second, because they critically depend on how speakers encode the target attitude in the first place, these studies offer no guarantee that what is encoded by the speaker actually corresponds to genuine prosodic signatures of certainty: speakers asked to display certainty may also convey social traits such as dominance (associated with lower pitch) or trustworthiness (associated with higher pitch)(Armstrong, Lee, & Feinberg, 2019; Ponsot, Burred, Belin, & Aucouturier, 2018); these social traits may mediate subsequent ratings of certainty, but the corresponding vocal signatures (e.g., mean pitch) cannot be presumed to be inherently related to certainty. Investigating separately the perception and production of these prosodic signatures is crucial in this regard: although of course they are intimately linked, they do not always rely on the same underlying mechanisms. For instance, mean pitch is not strictly tied to body size in speech production (formant dispersion is), still, listeners use this information to perceive body size and related social traits such as dominance, because of a general perceptual bias linking low pitch with largeness(Armstrong et al., 2019). Third, there are important differences between portrayed and spontaneous prosodic displays(Juslin, Laukka, & Bänziger, 2018): actors' productions may reflect stereotypical rather than veridical expressions, and in addition, they may not be aware of all the prosodic signatures that are naturally produced and used by listeners to perceive honesty and certainty. Finally, because acoustic features typically co-vary in speech production(Klaus R. Scherer, 2006), such paradigms do not allow examining how speech rate, pitch and loudness statically and dynamically impact listeners' perception independently from one other. In short, because these procedures are correlational in nature, they do not inform us about the underlying perceptual, cognitive and metacognitive mechanisms that drive these judgments."

Second, we now discuss the results of Study 1 by comparing them more systematically to previous literature. In particular, our finding that mean pitch is not a stable feature used by listeners to perceive certainty/honesty *"contrasts with previous findings examining prosodic signatures of certainty using elicitation procedures (Jiang & Pell, 2017; Van Zant & Berger, 2019), and with a previous study using a similar methodology and sample size, where lower pitch was found to significantly predict judgments about social dominance (Ponsot et al., 2018). By contrast, this is consistent with recent evidence suggesting that speakers' mean pitch is not necessarily impacted by their certainty in the absence of an audience, while intonation is (Goupil & Aucouturier, n.d.). Taken together, these findings suggest that mean pitch (i.e., a frequency code) is predominantly relevant for judgments concerned with the personal level (e.g., social traits of dominance), while dynamic pitch variations (i.e. intonation) are more relevant at the attitudinal level (e.g., of certainty, relating to effort or production codes)(Gussenhoven, 2002; Ponsot et al., 2018)»* (p.10).

Secondly (page 3), the question of "whether rising intonation and slower speech rate manifest in the same way for both honesty and doubt" can be addressed via research

by Hicks (2011), Nahari (2019), Perez-Rosas (2015), Shaw (2017), and Vrij (2018), to name only a few.

We have reformulated this part of the manuscript to improve clarity. But, we respectfully disagree that this is the case.

First, many of references cited by the reviewer are in fact not concerned with vocal communication (*Shaw, 2017 studying gaze aversion in video footage; Nahari, 2019 studying verbal rather than acoustical content; Hicks, 2011 studying video recordings with no acoustic analysis; Perez-Rosas, 2015 focusing on hand gestures and verbal content)* and thus, in our understanding, provide no element to address our specific research question.

Second, none of these studies, and no study to our knowledge, have considered how listeners attribute (un)certainty and (dis)honesty from natural inputs such as speech simultaneously, in a single research paradigm, and within the same participants, as we do here (a point that was also noted by R4, below). We note that a potential confusion may come from the fact that some studies collect observers' confidence judgements in their detections of deception, addressing a set of completely different research questions (e.g., see some examples in the review by Vrij 2019, or in some of the papers mentioned by the reviewer above). Noticing similarities between the prosodic displays of liars and uncertain speakers by remotely comparing disparate previous findings does not formally prove that there are shared underlying perceptual representations, especially when previous research provides discrepant results as noted above.

Finally, even in the absence of a direct empirical comparison of certainty and honesty, it is also not the case that the point has already been made theoretically that the perception of certainty and honesty are supported by the same core prosodic representation at a perceptual level, one we associate here with cognitive effort. The most articulated version of this theory may be, in our opinion, expressed in a paper by Gussenhoven (2002) cited in the initial version of our manuscript, which suggests a small number of core frequency, effort and production codes underlie the attribution of all natural meaning from intonation, but the paper only loosely associates frequency/pitch with certainty (assertiveness) vs uncertainty (questioning), and makes no mention of honesty/lie detection. Recent reviews (Vrij et al., 2019; tenBrinke et al., 2016) have also suggested that relying on prosodic signatures of uncertainty could support listener's responses in deception detection tasks. However, we maintain that this hypothesized connection between judgements of certainty and honesty has not been articulated and theoretically developed as we do here, and so far, it had not been directly substantiated by empirical data.

Therefore, we contend that, despite noted similarities (as well as unresolved discrepancies) in prosodic displays of certainty and honesty, we make here a totally novel contribution by 1) making the specific hypothesis that both judgements are based on a core prosodic signature at the perceptual level, although they differ at higher-order levels of processing, and 2) formally and empirically testing this hypothesis within a single empirical framework.

This being said, we totally agree with the reviewer regarding the interest of adding a discussion relating our finding to the deception detection literature more generally (i.e., not necessarily concerned with speech prosody). While the initial version of the paper only referred to a few papers in the discussion (tenBrinke et al., 2016; Zuckerman, 1981; Kassin,

2012…), we have significantly elaborated on this for the revised version of the paper (see also the reviewer's comment on lie detection below)

Third (page 4), "whether epistemic prosody can be perceived cross-linguistically" is not unknown as the authors suggest. The authors would do well to consider research by Johnson, Ernde, Scherer, & Klinnert (1986), Mandal (2008), and Pell, Monetta, Paulmann, & Kotz (2009), amongst others.

Again, we have to disagree with the reviewer's conclusion that previous literature had addressed this issue, although this comment highlights the fact that this part of the manuscript needed to be developed. We now dedicate a separate Figure and section to our cross-linguistic validation that should allow clarifying the novel contribution of the study.

The question of the cross-linguistic perception of vocal emotions is of course a classic and much-researched issue (for a recent review, see e.g. Laukka & Elfenbein, 2020), but no such study has formally tested the cross-linguistic perception of certainty and honesty (epistemic prosody) as we do here. For instance, the three studies mentioned here by the reviewer investigate the perception of happy, sad, angry and fearful emotions in various languages, but make no mention of either certainty or honesty. They are also not directly testing perception cross-linguistically, as we do here.

In our own review of the literature, we noted that past studies on prosodic signatures of certainty and honesty had examined only one language and attitude at once (e.g., Brennan & Williams, 1995; DePaulo et al., 2003; Dijkstra et al., 2006; Fish, Rothermich, & Pell, 2017; Guyer, Fabrigar, & Vaughan-Johnston, 2018; Jiang & Pell, 2016b, 2016a, 2017; Kimble & Seidel, 1991; Krahmer & Swerts, 2005; Pon-Barry, 2008; Roseano et al., 2016; Scherer, London, & Wolf, 1973; Smith & Clark, 1993; Spence, Arciuli, & Villar, 2012; Van Zant & Berger, 2019; Villar, Arciuli, & Paterson, 2013…), or compared the expression or perception of confidence or honesty across languages without testing whether these two attitudes can be recognized cross-linguistically (Chen & Gussenhoven, 2003; Spence et al., 2012). Only one study indirectly tested whether a composite attitude of doubt/ incredulity can be perceived across language in Japanese, French and English speakers from speech prosody alone, by relying on an elicitation procedure involving real utterances recorded in the three languages by trained speakers[59]. Findings suggested an in-group advantage in recognizing doubt-incredulity, but no acoustic analysis of the stimuli was provided, and it is unclear what was actually being encoded by the speaker in each language.

Therefore, we contend that we make here an unprecedented contribution by showing that prosodic signatures of reliability (honesty/certainty), learned from a data-driven experimental paradigm, can be perceived across languages. Even from a practical point of view, the generative model of vocal reliability developed here can be applied on words from at least three languages, which also vastly increase its use in future studies, e.g. *"to examine the impact of such features in collaboration or cooperation"* (point made by R1, we also come back to this in response to other comments by the reviewer below).

This being said, we agree that there was a need to develop this aspect of our manuscript, and we now briefly review the literature mentioned above on p.25 (l.595 to 609), separated the cross-linguistic aspect of the former Study 2 into a separate study (Study3), and discussed

how our result relates to research on vocal emotions showing an in-group advantage on p.27 (l.634 to 649).

Fourth (page 4), although certainly it is true that "our understanding of how we communicate honesty and confidence in speech is likely to be both inaccurate and incomplete", the fact that directly relevant, current research addressing this point (Guyer et al. 2018; Van Zant & Berger, 2019) was not even mentioned suggests that the authors could benefit from having conducted a more careful and thorough literature review.

We thank the reviewer for references to these two studies, which were published after the current study was initiated in 2017. Although these papers are only indirectly related to our research question (Van Zant & Berger, 2018: how speakers may deliberately use prosodic displays of confidence for persuasion; Guyer et al. 2018: how a listener's degree of motivation in understanding a message modulates their perception of vocal confidence), both references are indeed very relevant for the theoretical discussion, and we now included them in our manuscript in two directions.

First, we have added a discussion of how speakers can use confidence/honesty displays deliberately, as shown by studies in the persuasion literature: on p.6 *"…whether they are deliberately produced (Jiang & Pell, 2017; Van Zant & Berger, 2019), or automatically shown (Goupil & Aucouturier, n.d.; Kimble & Seidel, 1991), similar displays are observed…",* and again in the discussion, p.32/33: *"Now, speakers can also manipulate these natural signs deliberately during communication: a prosodic display suggestive of reliability (e.g., falling pitch and volume, faster speech rate) can be observed when a cooperative speaker deliberately shows it to signal confidence or to persuade (Jiang & Pell, 2017; Van Zant & Berger, 2019), …".*

Second, we have added a mention to Guyer et al., 2018 in our discussion of context effects: *"Relatedly, a recent study found that listeners' motivation to understand a message does not impact their perception of prosodic signatures per se, but influences how they exploit them to evaluate the message (Guyer et al., 2018)"*.

It should also be noted that Guyer et al. 2018 is one example of studies (Klofstad, Anderson, & Peters, 2012 is another) that do not only rely on acoustic analysis, but directly manipulate the acoustics of the recorded utterance, which constitutes an important improvement compared to purely correlational studies. Such procedures do not alleviate the need for the experimenter to make explicit assumptions about which features should be manipulated, but they constitute an important improvement as opposed to conventional elicitation procedures (also see comments above, and below detailing why).

Fifth (page 7), the pattern of effects observed for perceptions of honesty and confidence across intonation, pitch, loudness, and volume have been found in numerous prior studies. Thus, these findings add no new information regarding how variations in these vocal properties affect perceptions of confidence, honesty, and attitudes that has not already been shown in many studies within both the person perception and attitudes/persuasion literatures. (e.g, Brennan & Williams, 1995; Brown, Giles, & Thakerar, 1985; Guyer, Fabrigar, & Vaughan-Johnston, 2018; Jiang

& Pell, 2014; Kimble & Seidel, 1991; London, 1973; Scherer, London, & Wolf, 1973; Smith & Clark, 1993; Van Zant & Berger, 2019, etc.).

We have to disagree. While we agree that patterns of pitch, intensity and speech rate used in typical displays of confidence and honesty can be coarsely inferred based on previous research (baring a number of important discrepancies cited above, in particular in the case of honesty/deception), we think the above argument ("it adds no information of how these properties affect perceptions") critically misrepresents the implications of this prior literature, as well as the novel nature of our contribution.

The vast majority of work cited above has been the result of so-called "elicitation paradigms", in which actors (or, more rarely, participants in semi-naturalistic settings) are recorded while expressing utterances with various levels of confidence / honesty, and subsequent acoustic analysis is performed to correlate recognition performance data and specific acoustic features. While these paradigms are important to document patterns of vocal production, it is crucial to note that they do not offer direct mechanistic insight into listeners' perceptual representations, and how they may use such representations to make judgements of honesty and certainty. For instance, speakers asked to display confidence, or indirectly led to display confidence for e.g. persuasion, may also (or alternatively) convey social attitudes such as dominance (which previous data-driven studies have shown is signaled by lower pitch) or trustworthiness (signaled by higher pitch, e.g., see Ponsot et al. 2018). These attitudes may mediate subsequent ratings of confidence or honesty, but the corresponding vocal properties (e.g. mean pitch) cannot be presumed to be inherently communicative of epistemic information (and indeed we find here that they're not, in the case of mean pitch). More rarely, alternative procedures relied on acoustic manipulations to test the impact of specific features on persuasion (Klofstad, Anderson, & Peters, 2012; Guyer, Fabrigar, & Vaughan-Johnston, 2018), but again, such procedures heavily rely on experimenters' assumptions about which features should be manipulated manipulate and how, and they do not uncover the precise perceptual representations that support listeners' judgements per se.

In contrast, instead of acoustically analyzing naturalistic speech, here we use listeners' classifications of a large number of randomly manipulated pseudo-words to reconstruct the perceptual representations that underlie their judgments, in a purely data-driven manner. This procedure has three main advantages over previous approaches, as we now detail on p.8/9: *"First, it allows decorrelating perception from production by sampling agnostically from a large feature space rather than focusing on a smaller space sampled by experimenters* (Guyer, Fabrigar, & Vaughan-Johnston, 2018) *or actors (Jiang & Pell, 2017) and, thus, constrained by their own perception. Second, it allows an unconstrained and unbiased test of the hypothesis that attributions of certainty and honesty rely on a common prosodic signature at the perceptual level, by probing listeners' representations for the two attitudes separately before comparing them within the same frame of reference. Third, rather than correlating acoustical features with judgments, this reverse correlation procedure amounts to building a computational model in which observers make perceptual decisions by comparing exemplars to a fixed internal template (or, in the language of Volterra/Wiener analysis, a kernel), which the above procedure learns from decision data (Murray, 2011). This model can then be tested in a causal manner, by acoustically manipulating novel speech stimuli to match participants' kernels, and test their consequence on judgments made by other participants, as we do in Study 2, 3 and 4 below."*

In other words, with the novel data-driven methodology used in present work, and the open-source software CLEESE on which it is based (Burred et al., 2019), we do not provide a mere description of a prosodic signature, but we have built a model allowing to take any word recording in any language and to causally transform its dynamic profile of pitch, intensity and speech rate so to optimize the probability (based on the evidence from 115 participants in 4 languages) that it will be perceived as more certain or more honest. In three successive experiments, we then acoustically manipulate speech stimuli to display this common prosodic signature to "*(1) give mechanistic evidence that it is indeed implicated in both types of judgements when a context is provided (Study 2) and, as can be expected of a prosodic signature originating from physiological reactions associated with cognitive effort rather than stemming from socially learned conventions, show successively (2) that this signature is processed independently from participants' declarative concepts about epistemic prosody (Study 2), (3) that it is perceived cross-linguistically (Study 3) and (4) that it automatically impacts verbal working memory (Study 4)"* (p.6).

We hope to have clarified why this contribution is not a simple 'incremental' improvement over the descriptive approach of the existing literature.

Related to studies 1 and 2, it seems like the core take-home message is that the acoustic properties of voice that reflect confidence and honesty (and unconfidence/dishonesty) are independent of language. In other words, regardless of everything that differs between speakers of different languages, we all use highly similar acoustic markers to evaluate the extent to which a speaker is confident and honest.

Yes: on top of uncovering a core, fine-grained, dynamic prosodic signature supporting social attributions of both honesty and certainty, testing its relationship with conceptual knowledge, and examining how context impacts its processing, an important conclusion of our study is that this prosodic signature is perceived across languages.

I would encourage you to consider these questions at a more fundamental level. For example, what is it about pitch or loudness that leads people to infer confidence? Do variations in these acoustic properties of voice reflect physical or social power, dominance, importance, weight, substance, larger size, strength, etc?

We agree that these broader questions are particularly interesting. In the revised manuscript, we now clarified that the prosodic signatures that we describe are likely to be natural signs of cognitive effort, and thus, to be fundamentally related to specific mental attitudes or states, rather than social traits. By contrast, other aspects, such as mean pitch, may be linked to correlated but distinct social traits (e.g., dominance; see above for details). Distinguishing the acoustic features that specifically convey social attitudes, rather than associated social traits often associated with such attitudes, is in our opinion one of the contributions of the study.

Are these associations evolutionarily driven, and do these patterns also emerge among various species of primates?

Again, we agree that these questions are particularly interesting, but given that our study targets human listeners, this would remain very speculative at this point, and further quantitative research is required to provide a relevant discussion on the basis of empirical

data. Of particular interest is the fact that definitive behavioral markers of subjective confidence are still lacking to assess metacognition in animals (e.g., see discussions in Proust, 2007, 2012, 2019 vs. Carruthers 2009, 2013, 2016, 2019 among others), but whether epistemic prosody may constitute such marker remains an entirely open question at this stage.

Related to your conclusion on page 20 "Overall, words pronounced with unreliable prosodies were memorized better than reliable ones, and participants responded faster in this condition." It would seem to be evolutionarily adaptive to be able to identify acoustic markers that signal deception, given the diagnosticity of these cues as they relate to a recipient's attitudinal and behavioral responses to a source attempting to deceive.

The reviewer is right in his interpretation. This is one of the main question underlying our study, that is stated in the introduction on p.4: *"the highly adaptive function of epistemic vigilance (Mercier, 2020; Sperber et al., 2010) (…) suggest that (…) low-level mechanisms may have evolved to enable the fast and automatic detection of unreliability in social partners, across languages and cultures".*

We now further discuss this aspect on p.37 (l.878 to 886, also see response to R1).

So how do you explain these findings in light of a large body of research showing that both laypersons and professionals in careers where lie detection is of paramount importance (e.g, detectives, FBI agents, border control, etc.) perform no better than chance when attempting to detect lies?

We thank the reviewer for bringing up this point. Our initial manuscript was focused on describing the reliance on a common, language independent, and attention-grabbing prosodic signature supporting both judgements of honesty and certainty at a perceptual level, and we only briefly discussed the fundamental differences that exist between these two different attitudes. Following your comments and those of reviewer#3, we realize that this could lead to the impression that we are ignoring previous literature on the topic, and present a simplistic view where both judgments about certainty and dishonesty reduce to perception. We have now revised the manuscript to bring forward and discuss these aspects in more details.

Actually, research in the deception detection literature shows that observers are poor, but slightly better than chance at detecting liars (e.g., see the meta-analysis by Bond and dePaulo 2006 showing an accuracy of 54%). Two main hypotheses have been put forward to explain observers' low accuracy in overtly reporting liars, and our results are consistent with both, as we now discuss in the manuscript.

First, as research on deception detection suggests, it is not always the case that liars would display these markers (dePaulo et al., 2003; Vrij 2008; Hartwig and Bond 2011; Vrij et al., 2019), because vocal communication is humans is flexible, as we also discuss with reviewer #3 below. We have clarified this in our revised manuscript on p.32/33 (l.771 to 781): *"… speakers can also manipulate these natural signs deliberately during communication: a prosodic display suggestive of reliability (e.g., falling pitch and volume, faster speech rate) can be observed when a cooperative speaker deliberately shows it to signal certainty or to persuade (Jiang & Pell, 2017; Van Zant & Berger, 2019), but it can also be faked by a deceitful speaker in a coercive way (Vrij, Hartwig, & Granhag, 2019). Conversely, cues suggestive of unreliability (e.g., rising and variable pitch, slower speech rate) may be*

involuntarily disclosed by a dishonest speaker (Dezecache et al., 2013; Vrij et al., 2019). An important consequence of this flexibility is that there should be – in practice – no mandatory prosodic marker of dishonesty, as research analyzing liars' speech indeed suggests (DePaulo et al., 2003; Vrij et al., 2019), and thus, that perceiving a prosodic display suggestive of cognitive effort is not strictly conclusive in itself, as it is also crucial to determine whether the display was naturally or deliberately shown by the speaker (Wharton, 2009)."

Second, "*it has also been suggested that there is a dissociation between explicit and implicit (or intuitive) abilities to detect lies (Hartwig and Bond 2011; tenBrinke et al., 2016). While observers may be quite good at picking up relevant cues unconsciously (or intuitively), they may not always use them to overtly report dishonesty, in particular if they assess that "the costs of failing to detect deception (are inferior to) those of signaling distrust » (ten Brinke, Vohs, & Carney, 2016), or if they have been taught to rely on cues that turn out to be unreliable such as gaze aversion (Vrij et al., 2019)"*(p.34, l.808).

Previous studies on the topic (e.g., Hartwig and Bond 2011 or ten Brinke 2016…) did not directly compare measures of concepts and percepts as we do here, so the present results can be considered as providing the first decisive evidence regarding this issue in the context of speech prosody*: "we find that the prosodic signatures that are relevant to make these attributions are extracted automatically (Study 4), and that percepts and concepts about dishonest speech prosody largely dissociate (Study 2B)" (p.35).* We now elaborate on this aspect in the discussion on p.34/35 (l.815 to 840, also see in response to R1 above and R3 below).

I like the question you raised on page 23, linked to study 2: "This is compatible with an inferential model of mental attitude attributions, in which indices can be interpreted differently depending on the context in which they are perceived…" I wonder to what extent contextual factors such as the potential consequences of an error in judging the confidence/honesty of a speaker might moderate the threshold of the acoustic cues used to infer judgements of confidence/honesty. In other words, in contexts where the severity of the consequences for inaccurate evaluations of a source's confidence/honesty might be particularly high, we might also anticipate that the threshold used by message recipient's to evaluate perceptions of confidence/honesty may change by becoming more stringent.

We agree that this is a very interesting venue for future research. Our approach relying on reverse correlation and signal detection theory, which allows disentangling perceptual sensitivity and decisional biases, would allow asking this kind of questions. While the present Study 2 introduces a coarse manipulation of the context, further research manipulating finer aspects, such as the one you suggest here, or the ones reviewed by ten Brinke et al., (2016) or used by Guyer et al., (2018) would be really insightful. The revised manuscript now discusses possibilities for future research on this aspect: *"A promising venue for future research will be to combine our data-driven psychophysical method with specific manipulations of the context known to modulate the cost of exposing deceit (ten Brinke et al., 2016), or the exploitation of prosodic information to evaluate a speaker's message (Guyer et al., 2018), to examine whether - as suggest by Study 2 - these factors impacts decisional biases and contextualized inferences rather than sensitivity to prosodic signatures."*

I was somewhat surprised/confused by your comment on page 25: “This suggests that our results should apply to real spoken words embedded in discourse, but further studies should be designed to confirm this.” The reason for my surprise/confusion is, as previously noted, the fact that a growing body of research dating back more than 40 years has already extensively examined this, and other closely related questions.

Sorry for the confusion. This sentence was simply meant to acknowledge one of the limitations of our study, namely that the fine-grained profiles of pitch, intensity and speech rate uncovered here were derived from judgements of isolated pseudo-words and cannot simply be taken to generalize identically to words embedded in sentences, for which e.g., stress and cues to syntactic structure may also modulate the same acoustic properties. We now restate this point as follows: *“Here, we could not examine how phrasal prosody and word meaning interact with the prosodic signature that we observed: because we were interested in uncovering generic and language independent effects, our procedure involved isolated pseudo-words. Yet, previous research based on naturalistic speech has shown that certainty is mostly reflected in the prosody used to pronounce target words (i.e., words concerned by the epistemic marking, generally at the end of the utterance, Jiang & Pell, 2017; Pon-Barry, 2008), and that social attributions made on isolated words and sentences strongly correlate, and do not depend upon semantic content (Mahrholz, Belin, & McAleer, 2018), which suggests that our findings would generalize to real spoken words embedded in discourse.”*

Additionally, you asserted on page 25 that: “In addition, these findings shed light on the perceptual and metacognitive processes underlying such epistemic vigilance, and reveal that it can rely on implicit mechanisms that enable the quick and efficient detection of unreliability…” In my opinion, I’m not sure that any of these studies have included measures that would allow the researchers to systematically evaluate the underlying psychological mechanisms (mediators?) that might contribute to the perceptual and metacognitive processes driving these effects. In my view, this claim is not well supported by the methodology or analyses.

This sentence could have been misleading: as mentioned above, what we argue here is that our study offers unprecedented mechanistic insight, because it involves a series of hypothesis-driven experiments rather than a correlational, descriptive approach, that allows us to discriminate between the relative contributions of perception, concepts etc... Your comments made us realize that our procedure and approach may not have been clear enough in that respect, and we have now thoroughly modified the introduction and discussion to emphasize and clarify this.

Overall, the paper seems underdeveloped, presents too simplistic a view, and overlooks a considerable body of relevant, current empirical evidence that would contribute in important ways to the reader’s understanding of this phenomena. Simply revising the manuscript in an attempt to address coverage of missing literature would only, in my opinion, further highlight the largely redundant nature of the authors findings. To be fair, the authors do present data that advances existing literature on voice and person perception, though only in a very incremental fashion. In my opinion, the critical flaw in this manuscript is that the key questions that form the basis of these studies have already largely been answered by a considerable body of research in the person perception and attitudes/persuasion literatures over the last

40 years. Thus, I regrettably cannot recommend this manuscript for publication as I believe it lacks the novelty and significant advances in knowledge that warrant publication in Nature Communications.

We thank the reviewer for the constructive discussion. By clarifying the positioning of our manuscript with respect to the existing literature on certainty and deception detection, and the critical contribution of our data-driven procedure over the conventional methodology of elicitation studies, as well as clarifying our theoretical contribution, we hope to have elucidated why our study is not redundant, and how it directly tests important questions that were not answered by previous research, and opens up promising venue for future research.

Reviewer #3

In this ms., the Authors give participants pairs of pseudowords that have been manipulated to vary along a number of dimensions (pitch, volume, etc.). Participants are asked to tell which of the two pseudowords was spoken with the most confidence or the most honesty. The Authors can then extract the features of the pseudowords that best explain participants' decisions. On the whole, I believe this is an interesting ms. I know next to nothing about prosody, so I won't dwell on that, focusing instead on the theoretical framework.

We thank the reviewer for the positive comments that allowed us to clarify our theoretical framework.

I believe the Authors should more clearly indicate that the cases of confidence and of honesty are completely different. In the case of confidence, speakers have an incentive to communicate their degree of confidence. For instance, speakers might want to communicate that they are not so sure, in order to be blamed less if the information they communicate proves unreliable. As a result, every language has many confidence signals (for the terminology, see Scott-Phillips, 2008).

By contrast, when it comes to honesty, speakers have no incentive to communicate that they are being dishonest (except maybe in very rare occasions). As a result, if something in speech (or gesture) allows listeners to detect that a speaker is dishonest, it is a cue, and not a signal (again, see Scott-Phillips, 2008). Evolutionary theory suggests that there should be no reliable cues to dishonesty, as these cues are obviously costly for senders, and there is no good reason for them to exist. Indeed, as mentioned above, the literature on lie detection suggests that there are no such cues to dishonesty (based on behavior, gestures), and that, as a result, people are consistently at chance at detecting lies from truth using only such cues (by contrast with diagnostic evidence such as knowing the speaker's incentives to lie, or the content of the speech).

We agree. While the original manuscript did briefly mention this point ("lying speakers – by definition – do not deliberately signal their unreliability"), and made several related comments in relation with the second study, we have revised the manuscript to provide a clearer discussion of the difference between attributions of certainty and honesty, and the

relationships between the perceptual representations we uncover and how they can be used in different manners to make these two types of judgements.

We now expand on this aspect in the introduction on p.5: *"Genuine communication of certainty occurs when senders are actually willing to share their true commitment to the proposition they express (Joëlle Proust, 2012). Yet, it would also be adaptive to have means to detect speakers' commitment to a proposition when they are not willing to share this information (e.g., when they are trying to deceive). Even though lying speakers – by definition – do not deliberately signal their unreliability, dishonesty may also be detected from prosody if speakers involuntarily manifest signs of cognitive disfluency…"*

and on p.6 to 7 (l.141 to 162): *"… if social attributions of a speaker's certainty and honesty rely on similar perceptual inputs, then they may differ at higher levels of processing to allow listeners to differentially interpret these signatures for one or the other judgement. For social attributions of certainty, speakers' intentions should make little difference in interpreting the prosodic displays, since whether they are deliberately produced (Jiang & Pell, 2017; Van Zant & Berger, 2019), or automatically shown (Goupil & Aucouturier, n.d.; Kimble & Seidel, 1991), similar displays are observed. Crucially however, there is an important asymmetry in the case of dishonesty: on the one hand, signatures of cognitive effort may involuntarily be disclosed by a deceitful speaker, but on the other hand, a display suggestive of little cognitive effort may be deliberately shown to simulate certainty (Van Zant & Berger, 2019; Vrij et al., 2019). Thus, judgements about dishonesty can hardly reduce to perceptual decisions and would necessarily engage additional inferences (e.g., is the situation cooperative or competitive? what is the social cost of signaling dishonesty? etc.) in order to infer speakers' true intentions and interpret the display (Mercier, 2020; ten Brinke et al., 2016; Vrij et al., 2019). Given this, we hypothesized that, while judgments about honesty and certainty may rely on similar perceptual inputs, the type of inferences made upon these inputs would differ and lead to potentially different outcomes for contextualized judgments…"*

We will also come back to this in response to your other comments below.

As a result, the Authors appear to conflate signals of confidence and cues to honesty, even though these two things are quite different.

Given this, I would suggest that the best way to interpret the Authors' results is that participants are good at detecting confidence signals (such as volume), and that they then use exactly the same signals when they have to answer to the honesty question. I believe quite a lot of the Authors' data suggest this interpretation is likely correct. As the Authors state "These analyses demonstrate that, despite non-negligible inter-individual differences regarding the shape of these kernels, individual participants each relied on very similar mental prosodic representations to evaluate confidence and honesty." Moreover, for every feature associated both with confidence and with honesty, the association is stronger with confidence. There are no marker of honesty per se. Confidence is higher for confidence than honesty judgments.

We agree, this aspect deserves further explanation and clarification. Based on the available evidence, our favored interpretation is in fact that both are natural signs (or cues) of cognitive effort. Following Wharton, 2009, we assume that *« natural prosodic inputs fall into two importantly different categories - signals and signs"* where *"natural signs are interpreted by*

inference rather than decoding and are not inherently communicative at all" (p.14). The prosodic signatures we uncover are likely to fall in the latter category, as we now more explicitly argue below and in the manuscript.

We now carefully revised our terminology according to this interpretation. Initially, we stated on p.28 that these *"prosodic signatures (…) constitute indices"*, which we conceived as *"signs whose form has a causal but not physical relationship to the thing it represents"* (Scott-Phillips 2015, p.109), that *"can only be faked with great difficulty as a result of limitations on the organism*" (Green, 2007, p.6). However, since "indices" are typically taken to be strictly inflexible, like in the relationship between formant dispersion and body size, this term is not strictly appropriate here given that speakers can of course manipulate pitch, loudness and speech rate to fake these displays. In addition, we repeatedly used "signal" (verb) throughout, which added to the confusion.

To resolve this terminological imprecision that has deep conceptual consequences, we carefully revised our manuscript to use more theory-neutral terms such as signatures and markers throughout the paper, except in the discussion, where following Wharton, 2009 and Kimble and Seidl, 1991 (a paper showing no audience effect on these markers and referring to "vocal signs" of confidence), we use "natural signs", or following Scott-Phillips 2008 and Dezecache et al., 2013, and as suggested by the reviewer, "cues". We also corrected our use of "signal" (verb) or "signaling", replacing instances such as "common prosodic markers signal" by "convey", "support", … This, we hope, should clarify our framework.

More importantly, beyond this terminological issue, your remark also suggests a lack of clarity regarding our theoretical proposal. The reviewer's proposal is that the prosodic signatures we uncover are – fundamentally - signals in the case of confidence, and cues in the case of (dis)honesty. Although we agree with the latter point, the former assumption can be discussed: it is not clear that these prosodic signatures are necessarily genuine confidence signals, whose function is intrinsically communicative.

We revised the manuscript to discuss why we think that these signatures are likely to be signs rather than signals. First, as we now detail on p.5, because findings focusing on speech production suggest that they are actually acoustic consequences of producing speech with more effort: *'Interestingly, the markers of uncertainty identified in these studies closely resemble the acoustic signatures that have been associated to mental and articulatory effort: the tension and frequency of vocal fold vibrations – and thus pitch and pitch variability – increase with cognitive load (Yap, 2012) and psychological stress (Giddens, Barron, Byrd-Craven, Clark, & Winter, 2013; K. R. Scherer, Grandjean, Johnstone, Klasmeyer, & Bänziger, 2002). Higher effort is also associated with slower and more variable articulation rate (Berthold & Jameson, 1999; Yap, 2012), and with a disruption of the "default" pattern associating higher pitch and volume to the beginning of an utterance, and lower pitch and volume to the end of the utterance, a natural consequence of the decrease in subglottal air pressure during the exhalation phase of breathing (Gussenhoven, 2002; Le, 2012) (relatedly, prosodies intended to be neutral can actually be judged to reflect certainty(Jiang & Pell, 2017). Given the link between uncertainty and cognitive (dis)fluency (Ackerman & Zalmanov, 2012; Proust, 2012), it is probable that the prosodic signatures typically associated with states of uncertainty essentially constitute natural signs of cognitive effort, stemming from physiological constrains on speech production (Gussenhoven, 2002). If such was the case, we might expect that the same core prosodic signature may be used for other social attributions*

related to speaker reliability that are also thought to involve increased cognitive effort, such as lying...'

Second, research testing the communicative function of non-verbal behaviors typically examines whether the presence of an audience affects them (e.g., Kimble and Seidel, 1991; Dezecache et al., 2013; Krishnan-Barman & Hamilton, 2019…). Two studies have found that these prosodic signatures are not dependent on whether there is an audience or not (Goupil & Aucouturier, n.d.; Kimble & Seidel, 1991), suggesting that their function is not primarily communicative.

We now discuss this issue on p.32 and 33: *"An open question concerning this common prosodic signature is whether it is intrinsically communicative (i.e., a genuine signal that has evolved to affect receivers in particular ways, with receivers having evolved mechanisms to be affected by this very signal), or rather, constitute a natural sign or cue (i.e., characteristic of the senders receivers can draw inferences from)(Dezecache et al., 2013; Wharton, 2009). As mentioned above, it is likely that these markers stem from physiological constrains on speech production associated with cognitive (dis)fluency, that is, the amount of cognitive processes and mental effort that have been used by the speaker to produce the utterance (Gussenhoven, 2002; Zuckerman, DePaulo, & Rosenthal, 1981). The perceptual representations that we uncovered here with a data-driven method are very similar to the actual consequences of cognitive load on speech production (Gussenhoven, 2002; Yap, 2012). Research has also shown that speakers produce these prosodic signatures constitutively as a function of their certainty and competence, even in the absence of an audience (Goupil & Aucouturier, n.d.; Kimble & Seidel, 1991), which questions the view that they are fundamentally communicative* (Goupil & Aucouturier, n.d.)*. As such, they are likely to constitute natural signs (or cues) rather than signals (Scott-Phillips, 2015; Wharton, 2009): they can be interpreted by listeners, but their primary function is not to communicate. These displays may thus work like shivers, which are expressed naturally when one is cold, but can also be deliberately shown to ostensibly communicate that one is cold (Wharton, 2009)".*

Because it is probable that, originally, they stem from natural consequences of cognitive effort on speech production, and are present even in the absence of an audience, it seems more appropriate to assume that the signatures uncovered here are natural signs that "*carry information which provides evidence for a certain conclusion"* (Wharton, 2009; or cues) rather than signals (i.e., they did not "*evolve because of those effects*", Maynard and Smith Harper, 2003; Scott-Phillips 2008; Dezecache et al., 2013). They are a consequence of how cognitive effort affects speech production rather than being shaped by a communicative function.

This being said, in effect, and because vocal communication in humans is flexible, speakers can display these signatures in a variety of ways: ostensively, to genuinely signal their confidence to a partner, coercively, to fake confidence, or involuntarily, thus disclosing a cue that they are lying. We have now explicitly specified this on p.33 (l.770 to 780, also see above in response to R2).

To conclude on this point, we would like to emphasize the fact that – at core – the present study is concerned with perception, not production. This is crucial in our opinion, since as we now clarify on p.4, one of the important lessons of research on vocal displays is that perception and production sometimes dissociate: *"Investigating separately the perception and*

production of these prosodic signatures is crucial in this regard: although of course they are intimately linked, they do not always rely on the same underlying mechanisms. For instance, mean pitch is not strictly tied to body size in speech production (formant dispersion is), still, listeners use this information to perceive body size and related social traits such as dominance, because of a general perceptual bias linking low pitch with largeness (Armstrong et al., 2019)".

Ultimately, deciding on whether the prosodic signatures we describe here are cues or signals requires data that address the question of what these prosodic markers actually reflect, and how they are produced. This was not the aim of our study, as we have now clarified, and this is why we largely tried to avoid these debates in the previous version of the manuscript. We thank the reviewer for the opportunity to clarify the manuscript on that matter.

One way of testing the hypothesis that people are only processing confidence signals would be to pit the signals the Authors are studying against other information relevant to the speakers' confidence or honesty. My guess would be that the signals the Authors observed would still play a role when the participants evaluate confidence, but that they would essentially disappear if confronted with reliable information that someone is likely to be honest or dishonest (e.g. their incentives). I believe that the Authors should seriously consider this hypothesis (i.e. that participants are relying on confidence signals to answer the honesty question), and either explicitly endorse it, or then provide some arguments against it.

This is a very good point. One of the aims of Study 2 was precisely to test the role of contextual information, but a lot of discussion and results regarding this aspect was initially included in the supplementary materials for practical reasons (space). We agree that this important however, so we have now completely reworked this section to emphasize this aspect, and present individual results rather than group average, which should illustrate our point more clearly (see new Figure 3 in the paper).

In more details, the crucial difference between Study 1 and 2 is that, while Study 1 specifically targets perception and abolishes the influence of context and decisional biases (using a 2AFC procedure that forces participants to respond based on their sensitivity only, Study 2 involves a context (using a cover story about speaker's incentives) and allows the expression of individuals' decisional biases.

We have clarified this aim of Study 2A on p.19/20: *"To examine the hypothesis that judgments about certainty and honesty rely on a common prosodic signature at a perceptual level, but differ in terms of their reliance on additional, contextual information, we ran a second study…"* in which participants *"had to rate how much they thought that a speaker was lying (N=20, 9 females) or was certain (N=20, 12 females) on a Likert scale ranging from 1 to 7, in tasks where contextual information allowing to infer the speakers' incentives were now accessible. Note that – unlike the forced choice procedure used in Study 1 - such ordinal absolute ratings reflect a mixture of listeners' perceptual sensitivity (how attuned to sensory evidence their choices are), the contribution of other acoustical variables (e.g., voice timbre) and cognitive inferences based on these cues (e.g. speaker identity or gender), as well as individual decisional biases (listeners general tendency to report that someone is certain or honest)"* … *"Crucially, a context was now provided: for certainty, participants*

were told that the spoken words were responses recorded from other participants who previously performed a task with various levels of difficulty, and that they were to judge whether these participants were confident in their response or not. For the honesty task, they were told that their task was to judge whether previous participants engaging in a deceitful poker game were lying (i.e., bluffing) or not. Thus, participants rated the same stimuli in both tasks, but in one task the framing enforced an interpretation in terms of genuine expressions of certainty, while in the other task the framing suggested that the speakers would sometimes be deceitful, and sometimes not. This procedure allows us to examine the impact of individual decisional biases and contextual factors (i.e., information about speakers' potential incentives) on the interpretation of the prosodic signatures derived through our reverse correlation method in the first study, and to test the hypothesis that providing listeners with a context can sway their interpretations of these displays when the speaker is likely to be deceitful, while it does not in the case of certainty."

Crucially, we observed that additional contextual information led individual participants to interpret the displays in different directions in the honesty task, but not to dismiss them altogether as the reviewer suggests would be the case (in contrast, it had no effect in the certainty task, see details on p. 21 and on Figure 3A). The results show that prosodic signatures do play a role when incentives are made explicit, but that individual listeners interpret them either as suggesting that the speaker is dishonest or honest, presumably because they either infer that the speaker deliberately showed the prosodic signatures of confidence in the first case, or involuntarily disclosed them in the latter case.

These results therefore comfort our interpretation that listeners rely for both tasks on a core prosodic signature that originally stem from how effort impacts speech production. Listeners can use this signature to both infer that someone is doubting or that someone is lying, but in the latter case judgements involves more complex inferences about speakers' incentives, and whether the prosodic signatures are produced deliberately or not. This introduces more inter-individual variability in the judgements that these displays afford, due to yet to be explored factors (some effects of gender were observable at the individual level here, but as we now explicitly discuss on p.36, further research dedicated to this issue is clearly needed, see also discussion with R4 below).

Minor points:

"Research in linguistics has shown that some languages possess ostensive markers of epistemicity5,6, that speakers can use to deliberately communicate their level of confidence. These markers often consist in dedicated expressions such as "I don't know"7,8, but speakers may also rely on socio-pragmatic means to convey confidence (e.g., doctors may prominently point out that they are doctors)6,9. These markers are not present in every language and culture however5,10"

The last sentence is false: every language has means of encoding both modality and evidentiality. The Authors appear to be confusing the fact that modals or evidentials aren't grammatically mandatory in every language with the fact that modality or evidentiality can't be expressed. For example, English doesn't have evidentials, but it's possible to say in English "I saw" or "Peter told me" (or "I don't know" instead of a modal, in the Authors' own example).

Sorry for the confusing wording of this sentence. The sentence has been corrected to make a more straightforward point, namely that explicit markers vary from one culture/language to the next, and thus, require cultural learning, while it would be adaptive if lower-level, natural markers existed to infer speakers' reliability beyond cooperative interactions & cultural groups: *"Natural languages typically possess dedicated markers allowing speakers to explicitly and deliberately communicate their level of certainty (Aikhenvald, 2018; de Haan, 2001; Fusaroli et al., 2012). They can consist in dedicated expressions such as "I don't know" (Fusaroli et al., 2012; Juanchich, Gourdon-Kanhukamwe, & Sirota, 2017), rely on more indirect systems of evidentials, whereby speakers point towards the source of their knowledge (Aikhenvald, 2018; de Haan, 2001; González, Roseano, Borràs-Comes, & Prieto, 2017), or on socio-pragmatic means (de Haan, 2001; Roseano et al., 2016). To optimally share their certainty in such explicit ways however, partners need to calibrate the way they communicate their confidence to one another (Bang et al., 2017), and to converge on common linguistic expressions and systems through effortful processes involving conversational alignment and cultural learning (Fusaroli et al., 2012; Goupil & Kouider, 2019; Poulin-Dubois & Brosseau-Liard, 2016)."*

"even young children appear to filter information from unreliable informants" Given the wealth of evidence on this topic, the 'appear' should be removed. Absolutely, this has been corrected.

"Similarly to doubt, speech rate appears to decrease and pitch to increase during lies". On the whole the literature on lie detection suggests that all such cues are extremely weak and unreliable.

This is true, but increasing pitch is one of the only non-verbal cues that are actually quite reliable (see de Paulo 2003; Vrij 2019). We now describe this in more details on p.5/6: *"while lying is typically associated with increased cognitive effort (e.g. holding in mind two possibilities at once, having to build the details of invented situations, etc.)(Vrij et al., 2019; Zuckerman et al., 1981), research examining whether this has stable behavioral consequences has produced mixed results, and whether humans are actually able to exploit these cues to spot liars remains mysterious despite intense scrutiny (ten Brinke et al., 2016; Vrij et al., 2019). If anything, speech prosody is thought to carry more reliable markers of deception as compared to other behaviors such as gaze aversion (Bond & DePaulo, 2006; DePaulo et al., 2003; Villar et al., 2013; Vrij et al., 2019). Similarly to doubt, pitch tends to increase (DePaulo et al., 2003; Villar et al., 2013) during lies, and speech rate to decrease, although this latter relationship is less reliable (Fish et al., 2017; Spence et al., 2012; Vrij et al., 2019)".*

and discuss why these cues may be unreliable on p.33 to 35 (also see response to R2 above).

"Thus, the kernels were not affected by whether or not participants performed the other task beforehand (e.g., kernels appeared similarly in the confidence task whether or not participants had performed the honesty task before), which rules out an interpretation in terms of low-level strategies."
I don't understand what these low-level strategies are (they don't seem to have been introduced before)

Yes, this was unclear, we were concerned about potential carry-over effects from one task to the next. We have now clarified this (also see response to R4 below, point 5).

The Authors should justify their small Ns (maybe by pointing out the number of trials per participant, certainly with some power analysis). Given the high level of inter-individual variance, maybe a higher N would be desirable.

Our *"sample size was determined a priori based on a recent study using the same methodology (Ponsot et al., 2018)"*, as we now precise in the methods on p.38. In total our study involved 115 participants.

It would be nice to have effect sizes for all the results, a number of them being borderline significant.

This has been corrected by providing cohen's d for paired comparisons, partial eta squared for ANOVAs and betas for linear and logistic regressions, as well as chi-squares for likelihood ratio tests model comparisons.

Reviewer #4 (Remarks to the Author):

With three perceptual-acoustic studies, this study revealed the prosodic encoding and decoding of the reliability information in speech. The reverse-correlation analysis revealed a systematic change in the pitch, length and intensity cues that is commonly involved in making confidence and dishonest judgments. Such acoustic cues were independent of the listener's explicit knowledge of the link of the reliability attributes and the cues and of the listener's native tongue. Last but not the least, the speaker reliability affected the listener's memory performance. Moreover, the perceptual and memory outcome that were associated with the listener's meta-cognitive ability was also revealed across studies. These findings reveal a unique mechanism that supports the rapid detection and response towards speaker reliability cues in linguistic communication.

Overall this is a well-conducted study with rigorous statistic evidence that provides unique knowledge regarding the human perception of speaker reliability. The perceptual relevance of prosodic cues of one's mental states and how it is linked with listener's metacognitive ability is a highly relevant and widely neglected in the field of human nonverbal communication, given that no previous reports have considered (un)confidence and (dis)honesty simultaneously in a single research. Importantly, the study employed a data-driven approach (relative to a conventionally used elicitation paradigm to enhance the spontaneity of the speech) that explored (in study 1) and manipulated (in study 2) the acoustic features to examine the common prosodic cues that were explicitly (in study 1 and 2) vs. implicitly (in study 3) used by the listener to judge or recall speaker information.

We thank the reviewer for these positive comments that allowed us to clarify our manuscript.

In addition to the above merits, I have some minor comments that I hope to help strengthen the arguments.

1. The use of reverse correlation method is rigorous. I'm just not quite convinced how the perceptual judgment on "randomly" varied speech prosody is justified to identify the typical, representative cues that indicate confidence, and how the manipulation of the cues identified this way could generalize to the overall pattern of confidence. Based on recent study, the neutral prosody (even without any explicit manipulation of cues) can be perceptually judged as confident. The consideration of the current approach in this context would be very helpful.

This is a good point, which reflects a lack of clarity on our end regarding the contribution of the method. The rationale of using reverse-correlation on randomly varied stimuli is that it allows uncovering the mental representations that underlie listeners attributions of certainty and honesty directly, without relying on actor's productions, and thus, on their potential concepts about what constitutes certain/honest prosodies. This is important given previous research showing differences in portrayed versus spontaneous prosodic displays (Juslin et al., 2018), and the dissociation that we observe here between perception and conceptual knowledge in the second study.

We have now clarified the interest of sampling a large space through the use of random stimuli on p.8 and 9 (l.187 to 213, also see our responses to reviewer #2 above). An important detail is that these values are pseudo-random, and as we detail in the methods, were sampled from normal distributions whose parameters *"were chosen so as to cover the range observed in naturally produced utterances"* (p.39).

Regarding the relationship with neutral prosody, we agree with the reviewer that it is plausible that confident prosody corresponds to more of a "default" mode of speech production (e.g., descending pitch and volume), while the doubtful/dishonest prosody corresponds to rarer instances corresponding to utterances produced with more cognitive effort, which stand out. We clarified throughout the manuscript what we believe these prosodic signatures actually reflect, and specifically mention this aspect on p.5 (l.106, also see above in response to reviewer #3), notably by mentioning previous findings that are directly consistent with this idea: *"prosodies intended to be neutral can actually be judged to reflect certainty (Jiang & Pell, 2017)"*.

2. On p6, the acoustic extraction and analysis was based on 12 temporal points in the sample. However, in the method, the author mentioned the stimuli was created by random manipulation of acoustic values on FOUR consecutive windows. I was a bit confused but how was exactly the acoustic cue manipulated as the unfolding of the stimuli. Please clarify a bit here.

Sorry for the confusion, as we forgot to include this detail in the method section, this has been added on p.40: *"This analysis was conducted on the five original transformations values for duration (the last acoustic transformation to be performed). For pitch and loudness, the original values may not exactly correspond to what the listeners heard because the audio manipulations were executed in a sequential order, and subsequent transformations may have slightly altered the intended transposition. We thus ran acoustic analysis in 12 consecutive windows to obtain fundamental frequencies and RMS values for each stimulus, in order to*

remain as close as possible to the pitch and loudness that were actually heard by the participants".

We realize that this may be confusing for the reader, so we also added this information in Figure 1's caption: *"Kernels were computed for 5 time points for duration (corresponding to the initial values of the audio transformations) and in 12 time points for pitch and duration (corresponding to post-transformation acoustic analysis of the stimuli, see methods)".*

3. Fig. 1, a bit clarification regarding what the "filter amp" index would be helpful.
Thank you for pointing this out, we have added some information in the Figure caption to explain what it corresponds to: *"Filter amplitudes (a.u.) correspond to the values obtained for each participant, task, acoustic dimension and segment by subtracting the average (pitch, loudness and duration) values obtained for stimuli judged as certain/honest from the values averaged for the unchosen stimuli, and normalizing these values for each participant by dividing them by the sum of their absolute values."*

4. The instruction of the prosody task seemed asymmetrical, with the instruction of confidence task focusing on the positive end ("confident") while that of honesty task focusing on the negative end ("dishonest"). I'm curious how focusing on different ends of the scale of mental attributes could potentially drive the listener's perception. Some discussion would be needed.
As we mention in the methods (p.30 l. 729), this asymmetry was required by our design to avoid contagion effects: *"The order of the two experiments (confidence or honesty) was counterbalanced across participants, and the directionality of the responses was inverted across tasks: participants had to judge which of the two voices was the most confident in one task, and which of the two voices seemed to be lying in the other task, in order to avoid contagion effects"*. The fact that no differences were observed depending on task order rules out the possibility that this asymmetry impacted the findings in the first study relying on a forced choice design (also see below).

5. On the finding of common code in study 1. Could the similar reverse correlation kernels obtained along acoustic cues for confidence and honesty judgments be due to the same participants who was asked to judge on both attributions in a sequential order? The cues that were reliably significant in predicting the second social attribute in a sequence maybe influenced by that in the first. How could this possibility be ruled out from the findings?

This is a good point. We can rule this interpretation out because we counterbalanced task order, and checked that task order did not impact the findings, as we briefly mention on p.12, but now clarified as follows: *Importantly, there was no impact of task order on the kernels (i.e., whether the certainty or the honesty task was performed first; all p-values > 0.4), no interaction between task order and segment (all p-values > 0.5), nor task order, segment and task (all p-values > 0.4) for any of the three acoustic dimensions. Thus, the kernels were not affected by whether or not participants performed one or the other task before, which rules out an interpretation in terms of carry-over effects (i.e., participants keeping a strategy developed during the first task to perform the second task).*

6. While the study used shorter stimuli rather than the longer one (such as pseudo-sentence), how could the finding in the pseudoword be generalized to longer stimuli

in terms of the confidence/honesty encoding? The discussion can be meaningful given that the natural, longer speech stimuli may bring more variations in acoustic cues that contain dynamic information (e.g. intonation, accentuation, etc.).

This is an important issue. While reverse-correlation kernels inferred from responses to isolated words cannot simply be assumed to generalize to longer stimuli, as we now discuss on p.36/37 *"Previous research based on naturalistic speech has shown that certainty is mostly reflected in the prosody used to pronounce target words (i.e., words concerned by the epistemic marking, generally at the end of the utterance (Jiang & Pell, 2017)), and that social attributions made on isolated words and sentences strongly correlate, and do not depend upon semantic content (Mahrholz et al., 2018), which suggests that our findings would generalize to real spoken words embedded in discourse. This being said, it would be interesting to use our method, that allows a very fine-grained description of listeners' representations about intonations, to investigate how exactly phrasal and linguistic aspects of prosody interact with markers of reliability in speech".*

7. On the larger individual difference in perceiving honesty than confidence in speech. The honesty judgment may depend on more contextual factors (did speaker gender or any particular token affect the honesty perception?) and may be supported by a larger variation of acoustic cues. These discussions may need justifications by more analysis.

The original manuscript in fact already included this analysis, only in supplementary materials (see Figure SIII for Study 1 and Figure SVII for Study 2). As we now further discuss on p.36/37: *"Interestingly, gender differences were limited to explicit judgments (Study 1 and 2) while at the implicit level there were no differences between males and females (Study 3). In the second study, females were more likely to interpret certain/honest prosodies as faked displays when the context suggested that speakers could be deceitful (i.e., bluffing). This is consistent with research showing that females engage further neural processing upon hearing conflicting signatures to speakers' certainty (Jiang & Pell, 2016b), and documenting gender differences in deception detection (Burgoon, Buller, Blair, & Tilley, 2006). Whether the differences we observed here regarding speech prosody are linked to gender per se, rather than other related factors such as empathic or anxiety traits remains unclear given the strong correlation between these constructs (Greenberg, Warrier, Allison, & Baron-Cohen, 2018), and recent results showing that empathy and anxiety mediate gender differences in the neural processing of prosodic signatures of certainty (Jiang & Pell, 2016a). Studies involving bigger sample sizes and specifically designed to address this issue are needed to further understand how gender and other personal traits, as well as socialization and personal experiences, relate to the interpretation of (un)reliable prosodic displays. For instance, recent research involving twins showed that face impressions of trustworthiness are mostly determined by personal experiences rather than genes or shared environments (Sutherland et al., 2020).*

8. despite that study 2 included language groups other than French as out-group listeners, no language difference was found between French and these out-group listeners. Could the use of pseudo-speech reduce the perceived language and therefore prevent from any in-group advantage effect?

Yes, this is indeed a possibility. Here we wanted to test listener's ability to extract these signatures, so we used pseudo-words, but it is possible that in a study where the same

archetypes would be applied on real words, in-group advantages would emerge. Following the reviewer's suggestion, we now discuss this on p.27: *"Research has shown that many vocal emotions can be perceived across languages, although with substantial in-group advantages (Laukka & Elfenbein, 2020). Here, we find no in-group advantage regarding the perception of reliability in speech, which is consistent with the hypothesis that these prosodic signatures reflect a very basic phenomenon that is weakly influenced by social learning. Yet, we note that in-group advantages in recognizing vocal emotions increase as cultural distance increases (Laukka & Elfenbein, 2020), so future research should aim to test remotely related groups rather than Indo-European native speakers. Of particular interest for future research is whether this pattern of results would hold for native speakers of tonal languages (House, Karlsson, & Svantesson, 2016), and for native speakers of the few languages that do not conform to the default mode of speech production whereby most utterances present falling intonation and volume (e.g., in the few languages where rising intonations are used for statements)(Gussenhoven, 2002). Finally, it may be that in-group advantages are linked to a better familiarity with the utterances carrying the vocal prosodic signatures, as studies finding in-group advantages typically use real words/utterances. An open question is thus whether in-group advantages would emerge in a similar study involving real words/utterances rather than pseudo-words".*

9. On p22 despite that "the listeners use acoustic consequences of cognitive efforts as an index of reliability" is a very interesting point, study 2 still revealed differential interindividual differences between confidence and honest task. How could such account fit into these findings that demonstrate the distinction between the two tasks?

This is indeed an important point that we did not expand upon enough in the initial manuscript, as also pointed out by R3. In a nutshell, we argue that the same perceptual representations are used for both types of judgements, but that rational inferences based on these representations fundamentally differ for both types of social attributions, and crucially depend on context in the case of dishonesty (see in particular p. 8; 20/21 and 32/33).

In more details, the crucial difference between Study 1 and 2 is that, while Study 1 specifically targets perception and abolishes the influence of context and decisional biases (using a 2AFC procedure that forces participants to respond based on their sensitivity only), Study 2 involves additional contextual information (using a cover story about speaker's incentives) and allows the expression of individuals' decisional biases. We observed that, while it had no effect in the certainty task, additional contextual information led individual participants to interpret the displays in different directions in the honesty task (see new Figure 3 with individual results rather than group averages). These results show that individual listeners interpret acoustic consequence of cognitive effort either as suggesting that the speaker is dishonest or honest, presumably based on whether they infer that the speaker deliberately displayed these consequences in the first case, or involuntarily disclosed them in the latter case.

We have thoroughly revised our description of the aim of Study 2 on p.20, as well as the discussion of its results in p. 32/33 (see also responses to R3 above).

10. On p23 "individuals' ability to evaluate the inferences about social attitudes from prosody relates to empathic trait" This could be interesting however there is only

trend level difference in suggesting such relation between empathy and metacognitive ability. Some discussion in the context of recent findings demonstrating the link between vocal confidence and listener interactive traits would strengthen these arguments more.

We agree with your point, and similar comments by R2. This effect is indeed weak, and could be mediated by other factors that we did not control here. In addition, as the discussion with R1 suggests, this may be due to underlying differences in sensitivity as well. Given that this result is marginal, and given the current length of the paper and number of Figures, we have decided to not report this result anymore. Instead, we now discuss the possible contribution of empathy in relation to gender differences by referring to previous studies on the topic (see your point 7 above), and suggest this as a venue for future research.

11. How could the female advantage in deriving contextual meaning from the unreliable cue be linked in previous literature, given that some recent evidence showing similar advantage of using prosodic cues to infer mental states or resolving mixed cues in female and the increased sensitivity towards social information in these populations (e.g. Jiang & Pell, 2016)?

We thank the reviewer for this suggestion, we now discuss this aspect on p.32 (see citation above in response to your point #7). As mentioned above, we do not want to interpret gender differences too much here given that our sample size does not allow considering potential mediation effects by other factors such as anxiety or empathy (especially given previous reports that suggests that it may indeed be the case, for instance by Jiang & Pell, 2016a, as we now discuss), but our study and methodology certainly invites further investigations on that aspect.

12. The use of “confidence” for the perceptual task and “self-confidence” (and sometimes also “confidence”) for the metacognitive task is a bit confusing. Please correct whenever possible to avoid ambiguity.

We agree, and have changed the terminology in the paper: we now use ***confidence*** for listeners own confidence judgements about their social attributions, and ***certainty*** for social attributions about speakers’ levels of confidence (following a point also made by R1).

REFERENCES

Ackerman, R., & Zalmanov, H. (2012). The persistence of the fluency-confidence association in problem solving. *Psychonomic Bulletin and Review*, *19*(6), 1187–1192. https://doi.org/10.3758/s13423-012-0305-z

Aikhenvald, A. (2018). *The Oxford handbook of evidentiality*.

Armstrong, M. M., Lee, A. J., & Feinberg, D. R. (2019). A house of cards: bias in perception of body size mediates the relationship between voice pitch and perceptions of dominance. *Animal Behaviour*, *147*, 43–51. https://doi.org/10.1016/j.anbehav.2018.11.005

Arnal, L. H., Flinker, A., Kleinschmidt, A., Giraud, A. L., & Poeppel, D. (2015). Human Screams Occupy a Privileged Niche in the Communication Soundscape. *Current Biology*, *25*, 2051–2056.

Bang, D., Aitchison, L., Moran, R., Castañón, S. H., Rafiee, B., Mahmoodi, A., …

Summerfield, C. (2017). Confidence matching in group decision-making. *Nature Human Behaviour*, *1*(0117), 1–7.
Berthold, A., & Jameson, A. (1999). Interpreting symptoms of cognitive load in speech input. In *Lecture Notes in Computer Science (including subseries Lecture Notes in Artificial Intelligence and Lecture Notes in Bioinformatics)* (Vol. 407, pp. 235–244). Springer Verlag. https://doi.org/10.1007/978-3-7091-2490-1_23
Bond, C. F., & DePaulo, B. M. (2006). Accuracy of deception judgments. *Personality and Social Psychology Review*. https://doi.org/10.1207/s15327957pspr1003_2
Brennan, S. E., & Williams, M. (1995). The Feeling of Another′s Knowing: Prosody and Filled Pauses as Cues to Listeners about the Metacognitive States of Speakers. *Journal of Memory and Language*, *34*(3), 383–398.
Burgoon, J. K., Buller, D. B., Blair, J. P., & Tilley, P. (2006). Sex Differences in Presenting and Detecting Deceptive Messages. In K. Dindia & D. J. Canary (Eds.), *Sex differences and similarities in communication* (pp. 263–280). Lawrence Erlbaum Associates Publishers. Retrieved from https://psycnet.apa.org/record/2006-03342-014
Chen, A., & Gussenhoven, C. (2003). Language-dependence in the signalling of attitude in speech. *Proceedings of Workshop on the Subtle Expressivity of Emotion at CHI 2003 Conference on Human and Computer Interaction*.
de Haan, F. (2001). The Relation Between Modality and Evidentiality. *Linguistische Berichte*.
DePaulo, B. M., Malone, B. E., Lindsay, J. J., Muhlenbruck, L., Charlton, K., & Cooper, H. (2003). Cues to deception. *Psychological Bulletin*. American Psychological Association Inc. https://doi.org/10.1037/0033-2909.129.1.74
Dezecache, G., Mercier, H., & Scott-Phillips, T. C. (2013). An evolutionary approach to emotional communication. *Journal of Pragmatics*, *59*(B), 221–233.
Dijkstra, C., Krahmer, E., & Swerts, M. (2006). Manipulating Uncertainty: the contribution of different audiovisual prosodic cues to the perception of confidence. In *Speech Prosody*.
Fish, K., Rothermich, K., & Pell, M. D. (2017). The sound of (in)sincerity. *Journal of Pragmatics*, *121*, 147–161. https://doi.org/10.1016/j.pragma.2017.10.008
Fusaroli, R., Bahrami, B., Olsen, K., Roepstorff, A., Rees, G., Frith, C., & Tylén, K. (2012). Coming to Terms. *Psychological Science*, *23*(8), 931–939.
Giddens, C. L., Barron, K. W., Byrd-Craven, J., Clark, K. F., & Winter, A. S. (2013). Vocal indices of stress: A review. *Journal of Voice*. https://doi.org/10.1016/j.jvoice.2012.12.010
González, M., Roseano, P., Borràs-Comes, J., & Prieto, P. (2017). Epistemic and evidential marking in discourse: Effects of register and debatability. *Lingua*, *186–187*, 68–87. https://doi.org/10.1016/J.LINGUA.2014.11.008
Goupil, L., & Aucouturier, J.-J. (n.d.). Event-related prosody reveals distinct acoustic manifestations of accuracy and confidence in speech. https://doi.org/10.31234/OSF.IO/TUYNP
Goupil, L., & Kouider, S. (2019). Developing a Reflective Mind: From Core Metacognition to Explicit Self-Reflection. *Current Directions in Psychological Science*, *8*(4). https://doi.org/10.1177/0963721419848672
Greenberg, D. M., Warrier, V., Allison, C., & Baron-Cohen, S. (2018). Testing the Empathizing–Systemizing theory of sex differences and the Extreme Male Brain theory of autism in half a million people. *Proceedings of the National Academy of Sciences*, 201811032.
Gussenhoven, C. (2002). Intonation and interpretation: Phonetics and Phonology. *Speech Prosody 2002: Proceedings of the First International Conference on Speech Prosody*, 47–57.
Guyer, J. J., Fabrigar, L. R., & Vaughan-Johnston, T. I. (2018). Speech Rate, Intonation, and

Pitch: Investigating the Bias and Cue Effects of Vocal Confidence on Persuasion. *Personality and Social Psychology Bulletin*, *45*(3), 389–405. https://doi.org/10.1177/0146167218787805

House, D., Karlsson, A., & Svantesson, J.-O. (2016). When epistemic meaning overrides the constraints of lexical tone : a case from Kammu. Retrieved from https://lup.lub.lu.se/search/publication/959ad9f0-91df-4d19-98ae-370b4adff0a2

Jiang, X., & Pell, M. D. (2016a). Neural responses towards a speaker's feeling of (un)knowing. *Neuropsychologia*, *81*, 79–93. https://doi.org/10.1016/j.neuropsychologia.2015.12.008

Jiang, X., & Pell, M. D. (2016b). The feeling of another's knowing: How "Mixed Messages" in speech are reconciled. *Journal of Experimental Psychology: Human Perception and Performance*, *42*(9), 1412–1428. https://doi.org/10.1037/xhp0000240

Jiang, X., & Pell, M. D. (2017). The sound of confidence and doubt. *Speech Communication*, *88*, 106–126.

Juanchich, M., Gourdon-Kanhukamwe, A., & Sirota, M. (2017). I am uncertain" vs "it is uncertain". How linguistic markers of the uncertainty source affect uncertainty communication. *Judgment and Decision Making*.

Juslin, P. N., Laukka, P., & Bänziger, T. (2018). The Mirror to Our Soul? Comparisons of Spontaneous and Posed Vocal Expression of Emotion. *Journal of Nonverbal Behavior*. https://doi.org/10.1007/s10919-017-0268-x

Kimble, C. E., & Seidel, S. D. (1991). Vocal signs of confidence. *Journal of Nonverbal Behavior*, *15*(2), 99–105. https://doi.org/10.1007/BF00998265

Krahmer, E., & Swerts, M. (2005). How Children and Adults Produce and Perceive Uncertainty in Audiovisual Speech. *Language and Speech*, *48*(1), 29–53. https://doi.org/10.1177/00238309050480010201

Krishnan-Barman, S., & Hamilton, A. F. de C. (2019). Adults imitate to send a social signal. *Cognition*, *187*, 150–155. https://doi.org/10.1016/J.COGNITION.2019.03.007

Laukka, P., & Elfenbein, H. A. (2020). Cross-Cultural Emotion Recognition and In-Group Advantage in Vocal Expression: A Meta-Analysis. *Emotion Review*, 175407391989729. https://doi.org/10.1177/1754073919897295

Le, P. N. (2012). *The Use of Spectral Information in the Development of Novel Techniques for SpeechBased Cognitive Load Classification*.

Mahrholz, G., Belin, P., & McAleer, P. (2018). Judgements of a speaker's personality are correlated across differing content and stimulus type. *PLoS ONE*, *13*(10). https://doi.org/10.1371/journal.pone.0204991

Mercier, H. (2020). *Not born yesterday*. Princeton University Press.

Murray, R. F. (2011). Classification images: A review. *Journal of Vision*. https://doi.org/10.1167/11.5.1

Pon-Barry, H. (2008). Prosodic Manifestations of Confidence and Uncertainty in Spoken Language. In *Interspeech* (pp. 74–77).

Ponsot, E., Burred, J. J., Belin, P., & Aucouturier, J.-J. (2018). Cracking the social code of speech prosody using reverse correlation. *Proceedings of the National Academy of Sciences*, 201716090.

Poulin-Dubois, D., & Brosseau-Liard, P. (2016). The Developmental Origins of Selective Social Learning. *Current Directions in Psychological Science*, *25*(1), 60–64. https://doi.org/10.1177/0963721415613962

Proust, Joelle. (2012). Metacognition and mindreading: one or two functions? In M. J. Beran, J. L. Brandl, J. Perner, & J. Proust (Eds.), *Foundations of Metacognition* (pp. 234–251). Oxford, UK: Oxford University Press.

Proust, Joëlle. (2012). Conversational metacognition. In *Embodied Communication in*

Humans and Machines. https://doi.org/10.1093/acprof:oso/9780199231751.003.0015
Roseano, P., González, M., Borràs-Comes, J., & Prieto, P. (2016). Communicating Epistemic Stance: How Speech and Gesture Patterns Reflect Epistemicity and Evidentiality. *Discourse Processes*.
Scherer, K. R., Grandjean, D., Johnstone, T., Klasmeyer, G., & Bänziger, T. (2002). Acoustic correlates of task load and stress. In *7th International Conference on Spoken Language Processing, ICSLP 2002*.
Scherer, Klaus R. (2006). The Affective and Pragmatic Coding of Prosody (pp. 13–14). Springer, Berlin, Heidelberg.
Scherer, Klaus R., London, H., & Wolf, J. J. (1973). The voice of confidence: Paralinguistic cues and audience evaluation. *Journal of Research in Personality*, *7*(1), 31–44. https://doi.org/10.1016/0092-6566(73)90030-5
Scott-Phillips, T. C. (2015). *Speaking our minds : why human communication is different, and how language evolved to make it special*. Palgrave Macmillan.
Smith, V. L., & Clark, H. H. (1993). On the Course of Answering Questions. *Journal of Memory and Language*, *32*(1), 25–38.
Spence, K., Arciuli, J., & Villar, G. (2012). The role of pitch and speech rate as markers of deception in Italian speech.
Sperber, D., Clément, F., Heintz, C., Mascaro, O., Mercier, H., Origgi, G., & Wilson, D. (2010). Epistemic vigilance. *Mind and Language*, *25*(4), 359–393.
Sutherland, C. A. M., Burton, N. S., Wilmer, J. B., Blokland, G. A. M., Germine, L., Palermo, R., … Rhodes, G. (2020). Individual differences in trust evaluations are shaped mostly by environments, not genes. *Proceedings of the National Academy of Sciences*, 201920131.
ten Brinke, L., Vohs, K. D., & Carney, D. R. (2016). Can Ordinary People Detect Deception After All? *Trends in Cognitive Sciences*, *20*(8), 579–588. https://doi.org/10.1016/J.TICS.2016.05.012
Van Zant, A. B., & Berger, J. (2019). How the Voice Persuades. *Journal of Personality and Social Psychology*, *118*(4), 661–682.
Villar, G., Arciuli, J., & Paterson, H. (2013). Vocal Pitch Production during Lying: Beliefs about Deception Matter. *Psychiatry, Psychology and Law*, *20*(1), 123–132. https://doi.org/10.1080/13218719.2011.633320
Vrij, A., Hartwig, M., & Granhag, P. A. (2019). Reading Lies: Nonverbal Communication and Deception. *Annual Review of Psychology*, *70*(1), 295–317. https://doi.org/10.1146/annurev-psych-010418-103135
Wharton, T. (2009). *Pragmatics and Non-Verbal Communication*. Cambridge: Cambridge University Press.
Yap, T. F. (2012). *Speech Production Under Cognitive Load: Effects and Classification*.
Zuckerman, M., DePaulo, B. M., & Rosenthal, R. (1981). Verbal and Nonverbal Communication of Deception. *Advances in Experimental Social Psychology*, *14*, 1–59. https://doi.org/10.1016/S0065-2601(08)60369-X

<b>REVIEWER COMMENTS</B>

Reviewer #1 (Remarks to the Author):

I thank the authors for their comprehensive response to my comments. I have only minor queries remaining in relation to their additions to the manuscript:

- I was confused about how the (useful) metacognitive efficiency metric was calculated. The first-order sensitivity (slopes) are negative, but the efficiency metric is positive. I presume that this is because the absolute value was taken of the slope averaged over confidence levels, or similar, before computing the ratio? This is not clear in Methods on p. 41 which only mentions “absolute sensitivity” without defining what this means.
- I was also still a little confused about the SDT model. Can equations be given? I presume that “Note that only this noise source differs between repeated presentations of the same stimulus” isn’t exactly right: presumably the noise source (ie the Gaussian and associated sigma parameter) is maintained, but a new sample of noise is drawn from this distribution each time the same stimulus is presented (while the initial sample, drawn from the zero-mean Gaussian, stays fixed). Writing out the equations would help avoid any confusion here.

Reviewer #2 (Remarks to the Author):

It is clear that the authors have invested a considerable amount of time into revising their original contribution, which has now been substantially improved in all respects. I applaud the thoroughness of the revisions, and appreciate the clarifications on numerous issues raised by each reviewer. I believe the article now makes a greater and clearer contribution and is certainly deserving of publication.
joshua guyer

Reviewer #3 (Remarks to the Author):

The Authors have done a very impressive job responding to the Reviewers' comments, and I recommend that the ms. be accepted.

Reviewer #4 (Remarks to the Author):

In general, I was satisfied with the revisions made by the authors, which successfully addressed my comments in the my last review. I only have some minor points suggested for discussion.

1. P31: On the hypothesis "according to which this prosodic signature carries “natural” rather than

socially learned, “conventional” meaning": Assuming the listeners were highly socially sensitive and had pragmatic knowledge towards the prosody strategies of "reliability", I'm not sure if it is clear from the experiments whether the listeners relied on the socially-learned rule or the natural prosodic signatures to infer the speaker reliability when they were asked to judge pseudowords? if so, to what extent? Some further explanations or discussions would be very helpful.

2. On the application of reverse-correlation to the perceptual data based on the resynthesized stimuli: Given that the classification was based on the listener perceptual data and the nature of the stimuli was based on the resynthesis of speech, it can be interesting to know in future if the speaker (the sender) and the listener (receiver) would agree on the use of the set of prosodic cues towards reliability shown in the current experiments. Moreover, study 1 can also be extended by looking at a wider range of acoustic cues manipulated to see whether the shared prosodic representations of social attributes can be sufficiently robust and are independent of the selected sets of acoustic cues (e.g. speech rate, pitch and intensity).

RESPONSE TO REVIEWERS

Reviewer #1 (Remarks to the Author):

I thank the authors for their comprehensive response to my comments. I have only minor queries remaining in relation to their additions to the manuscript:

Thank you very much, these are very good points and we have followed these suggestions to improve the clarity of our method section.

- I was confused about how the (useful) metacognitive efficiency metric was calculated. The first-order sensitivity (slopes) are negative, but the efficiency metric is positive. I presume that this is because the absolute value was taken of the slope averaged over confidence levels, or similar, before computing the ratio? This is not clear in Methods on p. 41 which only mentions "absolute sensitivity" without defining what this means.

This interpretation is correct yes. To clarify this, we have reformulated this section as follows: *"Metacognitive sensitivity was then computed as the difference in the absolute values of the slopes of the psychometric curves computed for high confidence versus low confidence trials[52]. Metacognitive efficiency was then computed by dividing metacognitive sensitivity by the absolute value of sensitivity[78]. Because there is no clear, a priori ground truth in this experiment, absolute values of sensitivity were used to estimate metacognitive sensitivity and efficiency, which allows to specifically estimate how confidence judgements tracked the precision of decisions, over and beyond the idiosyncrasies that were observed at the perceptual level (that are reflected in the polarity of the relationship between decisions and sensory evidence).".*

- I was also still a little confused about the SDT model. Can equations be given? I presume that "Note that only this noise source differs between repeated presentations of the same stimulus" isn't exactly right: presumably the noise source (ie the Gaussian and associated sigma parameter) is maintained, but a new sample of noise is drawn from this distribution each time the same stimulus is presented (while the initial sample, drawn from the zero-mean Gaussian, stays fixed). Writing out the equations would help avoid any confusion here.

Absolutely. To clarify, we now provide the equations as suggested by the reviewer, and this section now reads as follows: *"Following an established procedure[49,50], we converted the percentages of agreement into values of internal noise using a computational signal-detection theory (SDT) model with response bias and late additive noise. This model assumes that the internal representation of each stimulus, before the addition of internal noise, follows a normal distribution centered on zero. Each representation is then corrupted by internal noise, modeled as a Gaussian noise source with standard deviation σ. Thus, the only thing that differs between repeated presentations of the same stimulus is the sample of noise, that is drawn randomly from the Gaussian distribution for each repetition. On each trial, the model selects the stimulus of the pair associated with the largest value, but is allowed to favor one interval over the other, which is implemented by a response criterion c that can differ from zero. Different values of σ and c lead to different percentages of similar responses given to repeated presentations of the same stimulus (percent agreement; referred to as pc_agree) and to different probabilities to select the first interval over the second (referred to as pc_int1) respectively.*

For each trial i, we can define:

s_i: the difference between the representation of the stimulus presented in the second interval subtracted from the representation of the stimulus presented in the first interval; s_i = s_interval_2 – s_interval_1, and follows a gaussian distribution with mean=0 and SD=1; it is the same for the two repetitions, because the stimuli are identical.

σ_{ir}: the difference between the noise sample added to the stimulus of the second interval subtracted from that of the first interval; σ_{ir} = σ_r_interval_2 – σ_r_interval_1, and follows a gaussian distribution with mean=0 and SD=σ; it is different between the two repetitions r1 and r2; we note σ_{i1} for the first repetition and σ_{i2} for the second repetition.

The SDT model with response bias c selects the stimulus presented in interval 1 if $(s_i + \sigma_i) < c$, and the one of interval 2 otherwise. There is agreement between the two repetitions (i.e., pc_agree = 1) if $((s_i + \sigma_{i1}) > c$ and $(s_i + \sigma_{i2}) > c)$ or if $((s_i + \sigma_{i1}) < c$ and $(s_i + \sigma_{i2}) < c)$. We ran computational simulations based on this formula to assess pc_agree and pc_int1 (averaged over the two repetitions) for different values of σ and c. A fitting procedure was then used to search for the specific values of σ and c that minimized the mean-square error between the values of pc_agree and pc_int1 predicted by the above model and the empirical estimates of pc_agree and pc_int1 observed in each task".

In addition, we have now included a Data and code availability statement, which should clarify the fact that all of the data and analysis scripts associated with this experiment are available on the Open Science Framework. Also, note that more details on our procedure to estimate internal noise can be found in the two following references, that are cited in the paper[3,4].

Reviewer #2 (Remarks to the Author):

It is clear that the authors have invested a considerable amount of time into revising their original contribution, which has now been substantially improved in all respects. I applaud the thoroughness of the revisions, and appreciate the clarifications on numerous issues raised by each reviewer. I believe the article now makes a greater and clearer contribution and is certainly deserving of publication.
joshua guyer

Thank you very much for your detailed assessment of our work, which greatly helped us to improve our manuscript.

Reviewer #3 (Remarks to the Author):

The Authors have done a very impressive job responding to the Reviewers' comments, and I recommend that the ms. be accepted.

Thank you very much for your detailed assessment of our work, which greatly helped us to refine our theoretical framework.

Reviewer #4 (Remarks to the Author):

In general, I was satisfied with the revisions made by the authors, which successfully addressed my comments in the last review. I only have some minor points suggested for discussion.

Thank you, these are really good suggestions, and we have now modified the discussion of the paper to include these aspects, that were certainly overlooked in the previous revision.

1. P31: On the hypothesis "according to which this prosodic signature carries “natural” rather than socially learned, “conventional” meaning": Assuming the listeners were highly socially sensitive and had pragmatic knowledge towards the prosody strategies of "reliability", I'm not sure if it is clear from the experiments whether the listeners relied on the socially-learned rule or the natural prosodic signatures to infer the speaker reliability when they were asked to judge pseudowords? if so, to what extent? Some further explanations or discussions would be very helpful.

This remark suggests that what we meant by “natural” vs. “conventional” may not have been very transparent. By “natural”, we mean “natural meaning” in the sense introduced by Paul Grice [5] with his example of “dark clouds mean rain”: a behavior naturally means X when such behavior is typically associated with X (here the prosodic signature is typically associated with unreliability). Thus, we are **not** employing “natural” by opposition with “artificial”, and indeed, our stimuli are all but artificial. By “conventional”, we mean a set of arbitrary rules (i.e., “conventions”) shared by a group of people, that have to be culturally learned, precisely because they are arbitrary, like words (i.e., unlike natural meaning, there is no form-function relationship between the conventional signal and what it actually means; see Clark, 1996; Lewis, 1969; or more recently, Scott-Phillips, 2015; Wharton, 2009).

The reviewer is right: the meaning of the prosodic signature we identify may still be socially learned in the sense that its meaning may be learned by observing conspecifics and registering associations (either explicitly or implicitly) between this specific prosodic signature and speakers’ reliability. Alternatively, they may be part of a more ancient system shaped by evolutionary pressures. We cannot conclude on this aspect here, but our findings that this prosodic signature of reliability is perceived across several languages, and is independent of conceptual knowledge, do suggest that it is not a (social or cultural) “convention”. As we argue in the paper, the available evidence instead suggests that this prosodic signature conveys “natural” meaning, in that it highly resembles the actual acoustic consequences of cognitive effort on speech production.

To clarify this, we now avoid the term “socially learned” throughout the paper, as we agree that this may create a confusion, and use “culturally learned”, “cultural learning” or “language dependent” instead.

We also introduce more clearly what we mean by “natural” on l.78, when the terms first appears: *“throughout the paper, we use « natural » by opposition with « conventional » to refer to meaning that relies on intrinsic and recurrent associations, rather than arbitrary, culturally learned conventions[5,6]”*, and specify how this relates to our predictions further on l.134-142: *“…we then acoustically manipulate speech stimuli to display this common prosodic signature. This allows us to: (1) provide mechanistic evidence that this prosodic signature is indeed implicated in both types of judgments in a contextualized situation (Study 2A), and to show that (2) it is processed independently from participants’ concepts about epistemic prosody (Study 2B), (3) it is perceived cross-linguistically (Study 3) and (4) it*

automatically impacts verbal working memory (Study 4), as would be expected of a core prosodic signature originating from physiological reactions associated with cognitive effort (i.e., of a natural sign) as opposed to a culturally learned convention".

Finally, we have included a sentence discussing the two possibilities mentioned above regarding how listeners may learn what this prosodic signature "means": "*An important open question for future research will be to understand the origin of this mechanism. It is possible that listeners learn what this prosodic signature naturally means by observing social partners, and registering associations between specific prosodic manifestations and speakers' reliability, attested independently via reasoning, or by observing other signs of cognitive effort (e.g., facial expressions, gestures…). Alternatively, this signature may be part of a more ancient system shaped through evolutionary pressures. Future research involving pre-verbal infants and non-human primates could shed light on this issue. For instance, recent research suggests that toddlers already have biases to focus on similar prosodic markers in child-directed speech, and that this may be associated with better learning[64]."*

2. On the application of reverse-correlation to the perceptual data based on the resynthesized stimuli: Given that the classification was based on the listener perceptual data and the nature of the stimuli was based on the resynthesis of speech, it can be interesting to know in future if the speaker (the sender) and the listener (receiver) would agree on the use of the set of prosodic cues towards reliability shown in the current experiments.

Thank you for this suggestion. We added a sentence to suggest this as a venue for future research on l.847: "*…given that our method allows a very fine description of the perceptual representations used by listeners to detect unreliability, it will be interesting in the future to directly compare these perceptual representations with speech produced in various naturalistic conditions, to precisely examine the extent to which the perception and production of unreliable speech overlap, and assess how accurate and sensitive to the context listeners are*".

Moreover, study 1 can also be extended by looking at a wider range of acoustic cues manipulated to see whether the shared prosodic representations of social attributes can be sufficiently robust and are independent of the selected sets of acoustic cues (e.g. speech rate, pitch and intensity).

Absolutely, thank you for this suggestion, we now mention this on l.921: *"Another potential extension of this study would be to examine other acoustic features that have previously been associated with certainty and/or cognitive effort, such as measures of voice quality[10,11]."*

REFERENCES

1. de Gardelle, V. & Mamassian, P. Weighting Mean and Variability during Confidence Judgments. *PLoS One* **10**, e0120870 (2015).
2. Fleming, S. M. & Lau, H. C. How to measure metacognition. *Front. Hum. Neurosci.* **8**, 1–9 (2014).
3. Burgess, A. E. & Colborne, B. Visual signal detection IV Observer inconsistency. *J. Opt. Soc. Am. A* (1988).

4. Neri, P. How inherently noisy is human sensory processing? *Psychon. Bull. Rev.* (2010).
5. Grice, H. P. *Meaning*. *Philosophical Review* (Philosophical Review, 1957).
6. Lewis, D. *Convention*. (Harvard University Press, 1969).
7. Clark, H. H. *Using language*. (Cambridge University Press, 1996). doi:10.1017/CBO9780511620539
8. Wharton, T. *Pragmatics and Non-Verbal Communication*. (Cambridge University Press, 2009).
9. Scott-Phillips, T. C. *Speaking our minds : why human communication is different, and how language evolved to make it special*. (Palgrave Macmillan, 2015).
10. Jiang, X. & Pell, M. D. The sound of confidence and doubt. *Speech Commun.* **88**, 106–126 (2017).
11. Yap, T. F. Speech Production Under Cognitive Load: Effects and Classification. (2012).

REVIEWERS' COMMENTS

Reviewer #1 (Remarks to the Author):

Thank you for the clarifications in response to my remaining comments. I have nothing further to add and congratulate the authors on an excellent paper.
